# Genomic characterization of chronic lymphocytic leukemia (CLL) in radiation-exposed Chornobyl cleanup workers

**DOI:** 10.1186/s12940-018-0387-9

**Published:** 2018-05-02

**Authors:** Juhi Ojha, Iryna Dyagil, Stuart C. Finch, Robert F. Reiss, Adam J. de Smith, Semira Gonseth, Mi Zhou, Helen M. Hansen, Amy L. Sherborne, Jean Nakamura, Paige M. Bracci, Nataliya Gudzenko, Maureen Hatch, Nataliya Babkina, Mark P. Little, Vadim V. Chumak, Kyle M. Walsh, Dimitry Bazyka, Joseph L. Wiemels, Lydia B. Zablotska

**Affiliations:** 1 School of Medicine, University of California, San Francisco, San Francisco, CA USA; 2 National Research Center for Radiation Medicine, Kyiv, Ukraine; 3 Rutgers-Robert Wood Johnson Medical School, New Brunswick, NJ USA; 4 Department of Pathology and Cell Biology, and Department of Medicine, College of Physicians and Surgeons, Columbia University, New York, NY USA; 5 School of Public Health, University of California, Berkeley, Berkeley, CA USA; 6 Division of Cancer Epidemiology and Genetics, National Cancer Institute, National Institutes of Health, Department of Health and Human Services, Bethesda, MD USA

**Keywords:** Ionizing radiation, Chronic lymphocytic leukemia, Mutation, Telomere, Chernobyl, Chornobyl

## Abstract

**Background:**

Chronic lymphocytic leukemia (CLL) was the predominant leukemia in a recent study of Chornobyl cleanup workers from Ukraine exposed to radiation (UR-CLL). Radiation risks of CLL significantly increased with increasing bone marrow radiation doses. Current analysis aimed to clarify whether the increased risks were due to radiation or to genetic mutations in the Ukrainian population.

**Methods:**

A detailed characterization of the genomic landscape was performed in a unique sample of 16 UR-CLL patients and age- and sex-matched unexposed general population Ukrainian-CLL (UN-CLL) and Western-CLL (W-CLL) patients (*n* = 28 and 100, respectively).

**Results:**

Mutations in telomere-maintenance pathway genes *POT1* and *ATM* were more frequent in UR-CLL compared to UN-CLL and W-CLL (both *p* < 0.05). No significant enrichment in copy-number abnormalities at del13q14, del11q, del17p or trisomy12 was identified in UR-CLL compared to other groups. Type of work performed in the Chornobyl zone, age at exposure and at diagnosis, calendar time, and Rai stage were significant predictors of total genetic lesions (all *p* < 0.05). Tumor telomere length was significantly longer in UR-CLL than in UN-CLL (*p* = 0.009) and was associated with the *POT1* mutation and survival.

**Conclusions:**

No significant enrichment in copy-number abnormalities at CLL-associated genes was identified in UR-CLL compared to other groups. The novel associations between radiation exposure, telomere maintenance and CLL prognosis identified in this unique case series provide suggestive, though limited data and merit further investigation.

**Electronic supplementary material:**

The online version of this article (10.1186/s12940-018-0387-9) contains supplementary material, which is available to authorized users.

## Background

Chronic lymphocytic leukemia (CLL) is the predominant type of leukemia among males in Western populations (30–40%) [1] and in Ukraine (> 50% in those 44 years and older) [2]. Validated CLL risk factors include male sex, longer telomere length, and several inherited genetic polymorphisms [3]. The clinical course of the disease is very heterogeneous, ranging from indolent to aggressive and rapidly progressive. Advances in microarray and sequencing technologies have identified genetic biomarkers of CLL as recurrent copy number abnormalities (CNAs), including del13q14, del11q22–23, del17p, trisomy-12, and frequent point mutations in *SF3B1*, *NOTCH1*, *BIRC3* and other genes [4]. Clinical characterization of these mutations has identified del17p, *TP53,* and *BIRC3* as markers of high-risk CLL, and *NOTCH1* and/or *SF3B1* as markers of intermediate risk [4].

It has been known, since the early 1950’s from the Hiroshima and Nagasaki atomic bomb (A-bomb) survivors’ study, that radiation exposure may induce most types of leukemia [5]. However, it generally has been accepted that radiation does not induce CLL. Until recently, the majority of epidemiological studies of occupational, environmental, or therapeutic exposure to radiation reported no excess risk of CLL [5]. Evidence suggests that mortality-based studies could underestimate, possibly substantially, CLL occurrence due to its benign clinical course, thus incidence studies are needed to characterize risks of low-dose radiation exposures [6]. Contrary to previous findings, recent incidence studies from our group [7, 8], other groups studying occupationally exposed radiation workers [9, 10], as well as the most recent update of the A-bomb incidence follow-up study [11], albeit based on a small number (12) of cases, reported significantly increased radiation risks of CLL.

In our recent study of the 1986 Chornobyl (Chernobyl) nuclear accident, CLL was the predominant leukemia in Ukrainian cleanup workers [7]. We reported significantly increased risks of CLL with increasing bone marrow radiation doses, which could not be explained by differences in lifestyle or environmental exposures [12]. Similar but statistically non-significant findings were observed for Chornobyl cleanup workers from Belarus, Russia and the Baltic countries [10]. Risk of CLL in Russian Chornobyl cleanup workers was not elevated [13], but questions have been raised about this analysis based on the official reported doses and the Chornobyl Registry-based leukemia diagnoses. Overall, there is now an emerging consensus on the role of ionizing radiation (IR) exposure in the etiology of CLL, but the magnitude of risks remains unknown. Further studies with large sample size of incident cases are warranted to understand the effect of IR on CLL.

One of the main hypothesized mechanisms underlying radiation-associated CLL is the absorption of energy from IR by genetic material leading to genomic instability [14]. Although studies have reported deregulated gene expression in CLL specimens obtained in the post-Chornobyl period [15], genetic characterization of Chornobyl-associated CLL has not been previously performed. Chornobyl cleanup workers present a unique opportunity to study the relationship between IR exposure and the genomic landscape of CLL after radiation. To better understand the genetic architecture of radiation-associated CLL, we performed comparative genomic analyses of Ukrainian Chornobyl cleanup workers exposed to IR (UR-CLL) with Ukrainian non-irradiated patients (UN-CLL) and Western patients (W-CLL). Although the UR-CLL sample is small, this is a unique series of confirmed CLL cases among Chornobyl cleanup workers with individual radiation bone marrow doses, confounder data and biological specimens. To our knowledge, this is the first such study of CLL cases after confirmed radiation exposures.

## Methods

### Patient recruitment

Cases of CLL among Chornobyl cleanup workers were obtained from the Ukrainian-American Study of Leukemia and Related Disorders among Chornobyl Cleanup Workers from Ukraine [7]. Data on patient characteristics and estimated doses were imported from the original study [7]. Briefly, we previously conducted a case-control study nested in a cohort of 110,645 male Ukrainian workers who were 20–60 years of age during cleanup activities in 1986–1990 after the Chornobyl nuclear power plant accident and who were registered in the Chornobyl State Registry of Ukraine (SRU) before 1992 and resided in Kyiv City, or in any one of five study areas. CLL cases diagnosed in the cohort during 20 years of follow-up (1986–2006) were pathologically confirmed by the International Hematology Panel consisting of five hematologists/ hematopathologists [16]. Bone marrow aspirates/ biopsy slides and/or peripheral blood smears were available for 70% of 79 confirmed CLL cases (UR-CLL) with estimated bone marrow doses. Only 16 cases had sufficient DNA for targeted next-generation sequencing (≥100 ng).

Study samples for unexposed Ukrainian CLL (UN-CLL) cases were from the patients treated at the National Research Center for Radiation Medicine (NRCRM) in Kyiv, Ukraine during 2002–2014. CLL diagnoses were confirmed by flow cytometry. From 119 available samples, we randomly selected males of comparable age and matched them to Chornobyl CLL cases in a ratio of 2:1. The final sample included 28 samples with sufficient DNA for targeted sequencing. An additional set of Western CLL (W-CLL) patients included 100 non-overlapping patients from the Dana Farber Cancer Institute (DFCI). To compare frequency of gene mutations in Ukrainian and Western CLL cases, exome sequencing data from DFCI patients was downloaded from dbGAP (phs000435.v2.p1).

### Patient characteristics

The following variables were examined for UR-CLL: the latent period (interval of time in years between the date of first exposure and the date of diagnosis of CLL)^1^; type of work performed in the 30-km Chornobyl zone (early responders, military personnel, professional nuclear power workers, other); calendar year of CLL diagnosis; time since first exposure; age at first exposure; age at diagnosis; average frequency of visits to the doctor prior to CLL diagnosis (zero, once every 2 years, more than once every 2 years)^2^; and smoking and alcohol consumption.

### DNA extraction and targeted sequencing

Blood smears for UN-CLL and UR-CLL cases were verified by study hematologists. Smears were scraped with scalpels (Miltex Inc., York, PA, USA) and DNA was extracted using QIAGEN QIAamp DNA Mini Kits (QIAGEN Inc., Valencia, CA, USA) as per manufacturer’s instructions. The purity of the slides was ascertained by determining the % of blasts. The average normal cell contamination was estimated at 15% for UR-CLL and UN-CLL slides. DNA concentration and quality were estimated by picoGreen mitochondrial DNA stain (Life Technologies, Carlsbad, CA, USA, Cat # P11495). CLL cases – 16 UR-CLL and 28 UN-CLL – were selected for deep-sequencing of cancer-relevant genes to discover novel driver mutations. All UR-CLL and UN-CLL specimens were confirmed to be pre-therapy to eliminate any therapy-induced effect.

For targeted deep sequencing (TDS), SeqCap EZ Exome Probes v3.0 (Roche Sequencing Solutions, Madison, WI, USA) were used to capture exonic regions of 538 genes included in the “UCSF500 Cancer Gene Panel” (Additional file 1: Table S1). Paired-end libraries were generated as per KAPA DNA Library Kits (Kapa Biosystems, Wilmington, MA, USA). DNA samples were barcoded and pooled for multiplexed sequencing on the Illumina HiSeq 2500 platform, which was run in Rapid run mode to obtain 130 million reads per lane. Sixteen samples were multiplexed with Illumina indexes and run per lane. On average, 80% of reads were retained after duplicate filtering, less than 3% were unmappable, and the remaining 17% of the reads were mapped to off-target regions. About 70% of the reads were mapped to target and near target areas of the bait.

Raw primary sequence data (.bam files) for W-CLL cases were re-aligned and mutations were called within the same genomic-capture regions as for the UCSF500 Cancer Gene Panel.

For data analysis, initial alignment of paired-end sequencing reads to the human reference genome (UCSC version hg19) was performed using the Burrows-Wheeler Aligner (BWA version 0.7.10-r789), with reads sorted by position and converted to compressed BAM format using SAM tools. Likely PCR and optical duplicate read pairs were marked using Picard (version 1.97(1504)). Insertion and deletion (INDEL) realignment and recalibration were carried out using the Genome Analysis Toolkit (GATK) (http://www.broadinstitute.org/gsa/wiki/index.php/The_Genome_Analysis_Toolkit). Single nucleotide variants (SNVs) and small sequence INDELs were called in each sample using the GATK command “Unified Genotyper”, with variant calls stored in VCF files.

Variant calls with total read depth less than 10X were excluded from further analysis for the lack of confidence in true variant calling. In the absence of matched normal sample from each tumor, to exclude likely germline polymorphisms we filtered out variants present in dbSNP or with a minor allele frequency > 0.01% in the Exome Aggregation Consortium (ExAC) Database. Coding variants predicted to affect protein sequence (e.g., nonsynonymous, stop gain, splicing) were analyzed further. To predict deleterious effects of variants, we used Polyphen, SIFT and Combined Annotation Dependent Depletion (CADD) tool version 1.3 (http://cadd.gs.washington.edu/score), which integrates information from multiple functional annotation tools into a single score. Finally, nonsynonymous variants of significant interest were visually inspected using the Integrated Genomics Viewer (http://software.broadinstitute.org/software/igv/).

### Copy number analysis

CNAs were analyzed using CNVkit [17] and off-target reads from the target area with capture probes. CNVkit was run with default parameters and female genome as reference. A threshold of 0.3 was applied to identify the signals for amplification and deletions of the genomic segments.

### Mutation and CNA prevalence calculation

Mutation and CNA prevalence was calculated as percent of cases harboring nonsynonymous mutations or copy number changes in a specific gene within a particular sample.

### Telomere length estimation

The telomere length was estimated by using off-target reads mapping to TTAGGG telomeric repeats, which has been shown to correlate with Southern blot measurements of the mean length of terminal restriction fragments (mTRFs) [18].

### NMF signature analysis

A non-negative matrix factorization (NMF) [19] was used to identify a radiation-associated mutational signature in exposed and unexposed Ukrainian CLL patients.

### Bone marrow dose estimation for exposed CLL cases (UR-CLL)

A time-and-motion method of retrospective dose reconstruction in cleanup workers, known as RADRUE, was developed for the study of cleanup workers from Ukraine [20, 21] and for a similar study conducted in Belarus, Russia, and Baltic countries [10] by an international group of scientists including experts from Belarus, France, Russia, the United States, and Ukraine. The method used combined data on work history from dosimetric questionnaires with field radioactivity measurements to estimate individual bone marrow doses for all study subjects. In-person interviews were conducted by trained interviewers and included questions concerning locations of work and residence while in the 30-km exclusion zone around the Chornobyl nuclear power plant, types of work, transportation routes, and corresponding dates. For deceased CLL cases, proxy interviews were conducted with next-of kin for demographic and medical information and with co-workers for work histories in the 30-km exclusion zone [21]. Additional validation studies have shown that bone marrow radiation dose estimates based on information from proxies were comparable to those based on direct interviews [22].

### Statistical methods

All analyses relied on cumulative radiation doses derived as the sums of the arithmetic means of the annual 1986–1990 bone marrow doses estimated by generating 10,000 realizations of dose predictions from the RADRUE method as described above.

Univariate tests of normally distributed continuous variables were performed using one-way analysis of variance (ANOVA), and on non-normally distributed variables using a Wilcoxon– Mann–Whitney test. Additional multivariate analyses were conducted using Poisson regression for genetic lesions, total number of genetic lesions and mutations and telomere length, and using logistic regression for *POT1* mutations, and included categorical as well as continuous predictors.

All *p*-values presented were two-sided. The best fitting models were chosen by using the likelihood ratio test and Akaike Information Criterion. All analyses were conducted using the SAS 9.4 software (SAS Institute, Cary, NC, USA).

## Results

This analysis is based on the 16 cases who had sufficient DNA for targeted next-generation sequencing drawn from the parent study of 79 CLL cases from the Ukrainian-American Study of Leukemia and Related Disorders among Chornobyl Cleanup Workers from Ukraine [7]. Selected cases did not differ from all cases in terms of age at first exposure, age at CLL diagnosis or radiation dose (all *p* > 0.1, not shown).

UR-CLL and UN-CLL samples were sequenced to ~450X depth and compared to W-CLL. Total numbers of nonsynonymous point mutations across the 538 cancer-relevant genes were comparable in UR-CLL (range 2–12, median 8), UN-CLL (range 2–12, median 8), and W-CLL (range 2–11, median 8) samples. CADD Phred scores indicated no statistically significant difference in deleteriousness of detected mutations across the three groups.

We further analyzed the correlation of genetic lesions (total number of mutations + CNAs) with several clinical variables (Table 1) and bone marrow radiation dose (median: 40.56 milligray (mGy); range: 0.24–1536.24 mGy). The type of work performed in the 30-km Chornobyl zone, age at first exposure, age at diagnosis, calendar time and Rai stage of CLL were identified as significant predictors of genetic lesions (all *p* < 0.05), together explaining 20% of their variability (combined pseudoR^2^ = 0.20). Adjusting for all other factors, Chornobyl CLL patients who had more advanced stage at diagnosis (Rai stage ≥2) had a two-fold higher predicted number of total lesions compared to those diagnosed at a less advanced stage.

**Table 1:** Characteristics of Ukrainian CLL cases

Variable	Exposed (UR-CLL)^a^	Unexposed (UN-CLL)^b^
Cases, N	16	28
Age at diagnosis, median (range), years	61 (49–78)	58 (41–76)
Sex	Male	Male
Diagnosis year, range	1986–2006	2002–2014
Blood count, median (range), 10 × 9 per mL	13 (2–55)	54 (16–122)
Rai stage, n (%)		NaN^c^
0	1 (6)	
1	3 (19)	
2	9 (56)	
3	3 (19)	
Smoking history, n (%)		NaN^c^
never or former	9 (56)	
less than 20 cigarettes/day	5 (31)	
20 or more cigarettes/day	2 (13)	
Alcohol consumption		NaN^c^
never	4 (25)	
no more than 2–3 times a month	7 (44)	
once a week or more	5 (31)	
Bone marrow radiation dose, median (range), milligray (mGy)	40.56 (0.24–1536.24)	NA^d^
*POT1* mutation, n (%)	4 (25)	0 (0)
Telomere length, median (range), kilobytes (kb)	1.58 (0.12–9.06)	0.30 (0.09–2.47)
Survival after diagnosis, median (range), years	4 (1–18)	NaN^c^

Based on 15 cases who have died by the end of follow-up

^a^CLL cases in Chornobyl cleanup workers from Ukraine exposed to ionizing gamma-ray radiation

^b^Sex- and age-matched CLL cases from the general population of Ukraine unexposed to ionizing radiation

^c^NaN = Not Available

^d^NA = Not Applicable

**Fig. 1:**
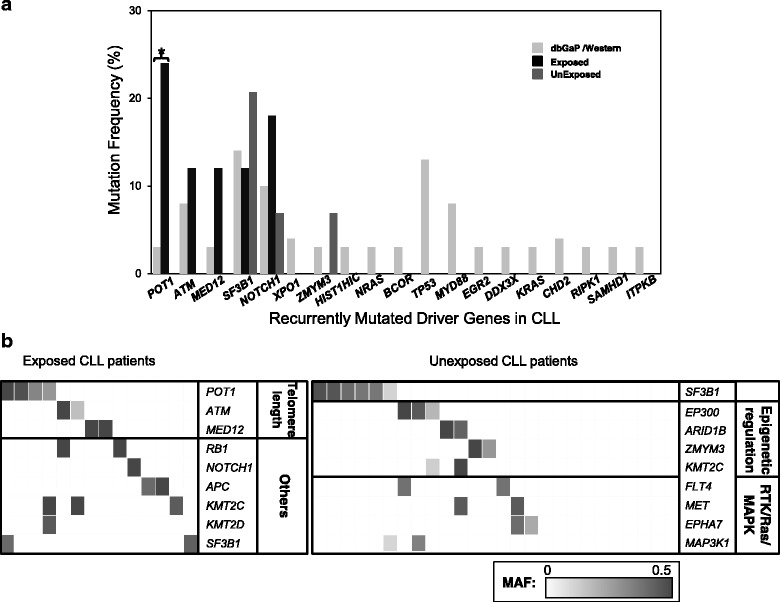
Genomic landscape of CLL cases. (**a**) Prevalence of driver mutations, as defined in Landau et al. [23] in Western-CLL (dbGaP/Western; W-CLL cases from previous sequencing studies, light gray bars), Chornobyl cleanup workers with CLL (Exposed; UR-CLL, black bars) and unexposed Ukrainian CLL cases (UnExpsed; UN-CLL, gray bars). The asterisks identify significant comparisons (*p* < 0.05); (**b**) Tiling plot of predicted damaging mutations in exposed (UR-CLL) and unexposed (UN-CLL) Ukrainian patients. Horizontal rows depict mutations in a particular gene and functional category, and vertical columns represent individual patients (exposed and unexposed). Patients without mutations were excluded from the figure. Mutations are shaded according to their mutant allele fraction (MAF)

We compared the prevalence of putative CLL driver mutations across three samples, and *NOTCH1* was the most frequently mutated gene overall and was mutated at similar frequencies across the three samples (Fig. 1a). *POT1* was the most frequently mutated gene in UR-CLL (25%), followed by *NOTCH1, RB1 (*19% each), *ATM, APC, MED12, SF3B1*, and *KMT2C* (13% each) (Fig. 1a and Additional file 1: Table S2). The most common CNAs identified in UR-CLL were del13q14 and del11q (12% each) (Additional file 1: Table S3). No statistically significant differences were identified in frequencies of CNAs between UR-CLL, UN-CLL and W-CLL.

**Fig. 2:**
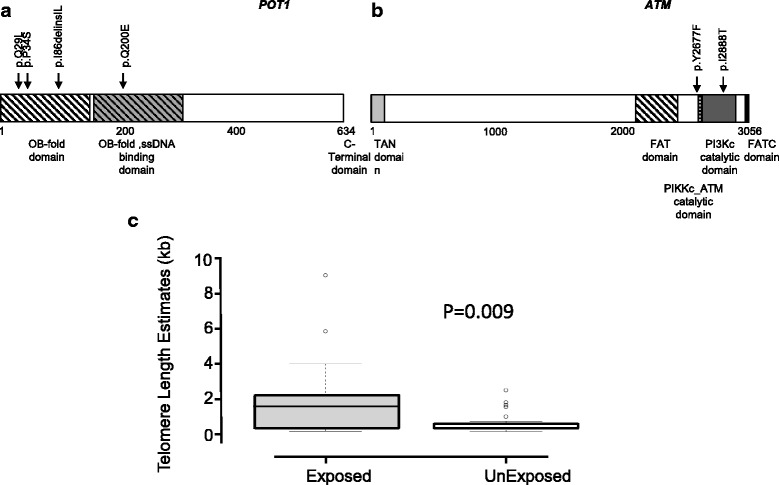
Telomere length comparison and associated mutations (**a**) Location and type of mutations associated with telomere maintenance pathway genes *POT1* and (**b**) *ATM*. (**c**) Boxplot of estimated telomere length between exposed (UC-CLL) and unexposed (UN-CLL) Ukrainian samples. Fifty percent of the data is within the box, 75% within the whiskers, and outliers are noted by circles. A black line within the box depicts the median telomere length for the group

To further delineate genomic differences between radiation-associated CLL and idiopathic CLL, an NMF approach [19] was applied. UN-CLL tumors were enriched for mutations in genes with roles in epigenetic regulation (*EP300*, *ARID1B*, *ZMYM3*, *KMT2C*) and the Ras/MAPK signaling pathway (*FLT4*, *MET*, *EPHA7*, *MAP3K1*), consistent with previous studies (Fig. 1b) [23]. We searched for a potential radiation-associated mutational signature in UR-CLL, but a specific pattern of preferred nucleotide substitutions could not be resolved. Next, we performed pathway analyses and identified mutations in telomere-maintenance pathway genes to be enriched in UR-CLL. We observed a significantly higher frequency of *POT1* mutations in UR-CLL compared to both the UN-CLL and W-CLL cases ( *p* = 0.03 and 0.009, respectively). Further, recurrent mutations were found in *ATM, RB1*, and *MED12* in UR-CLL (Fig. 1b). All 4 *POT1* mutations detected in UR-CLL were localized to OB-fold domains 1 & 2 (Fig. 2a), while recurrent *ATM* mutations were localized to other functional domains (Fig. 2b).

Since *POT1* haploin sufficiency has been reported to cause telomere elongation in CLL and glioma [24], we estimated telomere length via telseq [18] and identified a significantly longer age-adjusted mean telomere length in UR-CLL tumors compared with UN-CLL (Table 1 and *p* = 0.009 in Fig. 2c).

We further analyzed telomere length correlation with other covariates and identified lifestyle factors such as alcohol consumption, smoking and type of cleanup work performed in the Chornobyl zone as significant predictors of tumor telomere length in UR-CLL (combined R^2^ = 0.63). In a combined analysis of telomere length among UR-CLL and UN-CLL groups, age was not a predictor of tumor telomere length (*p* = 0.17) whereas exposure to radiation was a significant and strong predictor (1.59 unit increase in length due to radiation (95% confidence interval: 0.64, 2.55, *p* < 0.01)). In total, IR explained 20% of the variability in telomere length. As *POT1* and *MED12* mutations have been previously associated with poor prognosis in CLL patients [24, 25], we tested the trend of median survival of the 4 *POT1*-mutated UR-CLL cases. Results were not significantly different from the other UR-CLL cases, but a trend was observed indicating better prognosis of *POT1* mutation bearing-cases (median 5.3 years vs. 3.6 years, *POT1*-mutated vs. other UR-CLL cases, *p* = 0.74).

## Discussion

This study was conducted to understand the effect of IR on CLL development and progression in a series of high-quality DNA specimens with corresponding radiation dosimetry and clinical information. Radiation exposure arose from a ‘natural experiment’ (i.e., an individual’s exposure status was determined by outside forces but resembled random assignment). To our knowledge, this is the first study of genetic characteristics of CLL specimens in relation to individual radiation doses. Unfortunately, we could not perform this study on a larger sample set, and therefore the findings described here should be treated as a valuable case-series with unique preliminary and valuable data for guiding future research hypotheses.

Absence of statistically significant differences in frequencies of CNAs between UR-CLL, UN-CLL, and W-CLL indicated a similar genomic architecture in radiation-exposed compared with unexposed cases, and in Ukrainian and Western cases.

Sherborne et al. identified mutation signature significantly associated with high-dose (total dose of 30 Gy) IR in multiple IR-induced malignancies [19]. We used their non-negative matrix factorization technique to identify IR-associated mutation signatures above genetic background in UR-CLL. Specific pattern of preferred nucleotide substitutions, associated with IR exposure could not be resolved in mutations accumulated and identified in UR-CLL. This suggests that protracted exposure to low-dose radiation did not appear to induce widespread genomic changes via a specific mutational mechanism. However, our study might be underpowered to detect such a signature due to an insufficient number of mutations observed per case or the relatively low bone marrow doses estimated for UR-CLL patients. Low bone marrow doses of UR-CLL might also explain the lack of expected genomic instability.

We identified enrichment of mutations in genes with roles in epigenetic regulation (*EP300*, *ARID1B*, *ZMYM3*, *KMT2C*) and the Ras/MAPK signaling pathway (*FLT4*, *MET*, *EPHA7*, *MAP3K1*), consistent with previous studies [23].

Although we did not identify a radiation-associated mutation signature in UR-CLL cases, our pathway analyses did reveal an enrichment of mutations in telomere-maintenance genes. *POT1*, *ATM*, and *RB1* all have reported roles in telomere maintenance [26]. The most frequently mutated gene in UR-CLL was *POT1* (*Protection of Telomeres 1*), and it was mutated at significantly higher frequency than in UN-CLL or W-CLL. *POT1* is one of six members of shelterin, a protein complex that binds telomeres. Additional shelterin complex proteins are encoded by *TERF1*, *TERF2*, *TINF2*, *TERF2IP*, and *ACD*. Three shelterin subunit proteins, including POT1, directly bind to the telomeric hexanucleotide repeats [27]. Previous studies have shown that localization of mutant *POT1* protein to the telomere causes dominant-negative telomere lengthening and telomere uncapping, leading to unprotected telomere ends and chromosomal fusions in CLL tumors [24]. Interestingly, all 4 *POT1* mutations detected in UR-CLL lie in OB-fold domains 1 & 2, which interacts with telomeric DNA and provides specific binding to various ligands [28]. These results suggest that we have identified functional *POT1* mutations likely to be involved in telomere uncapping and telomere lengthening. Similarly, recurrent *ATM* mutations that we identified were also localized in functional domains. Although no direct association of *MED12* has been identified with telomere biology, *MED12* mutations in association with *TERT* promoter mutations and increased telomere length have been reported in different tumors [29].

Previous studies suggest that radiation exposure is associated with telomere attrition [30], especially in males. However, studies of Chornobyl cleanup workers reported associations between longer telomere length and increased cancer diagnoses [31]. This is particularly compelling for CLL, as recent studies have established longer telomere length in healthy lymphocytes as a risk factor for future development of CLL [32, 33]. Our observation that radiation exposure was associated with longer telomere length in UR-CLL tumors suggests that pre-malignant B-cells in radiation-exposed men may be under strong selective pressure to circumvent growth arrest caused by telomere attrition. Somatic cells can undergo a host of different genetic and epigenetic mechanisms to lengthen telomeres, including reactivation of telomerase or alternative lengthening of telomeres (ALT). The observed frequency of *POT1* mutations in UR-CLL (25%) is much higher than in recent studies of radiation-unexposed CLL patients (3.5% to 9% [24]). Our observation that UR-CLL patients were likelier to harbor mutations in telomere-maintenance genes fits this proposed model.

Although survival of UR-CLL patients with *POT1* mutation was longer compared to other UR-CLL patients, this finding was based on a small number of cases and should be examined in future studies. Follow-up of all 79 CLL cases diagnosed in the Ukrainian-American Study of Leukemia and Related Disorders among Chornobyl Cleanup Workers from Ukraine indicated median overall survival of 4.8 years (range 0.5–19.5, 5-year survival rate of 46.2%) [8]. This is substantially lower than survival of U.S. CLL patients from the SEER database (5-year survival rate of 83.2%) [34].

Although the incidence of CLL is substantially higher in Ukraine than in Western countries, we observed a great deal of similarity between the somatic genomes of UN-CLL cases and W-CLL cases. Both groups had frequent mutations in *NOTCH1*, had similar CNA profiles, and were enriched for mutations in genes with roles in epigenetic regulation and the Ras/MAPK signaling pathway. These data suggest that there is a similar somatic genomic architecture in non-irradiated Ukrainian CLL patients and in Western CLL patients. Therefore, the differences in incidence rates of CLL across these groups may be attributable to other factors, such as the frequency of heritable genetic variants or the prevalence of other environmental risk factors.

## Conclusions

We conducted a comprehensive three-way comparison of UR-CLL, UN-CLL and W-CLL to understand the relationship of IR with CLL etiology. Ukrainian CLL patients with no history of radiation exposures had similar somatic genomic architecture to Western CLL patients. Our analysis of CLL patients exposed to IR due to clean up work after the Chornobyl accident suggests that dose to the bone marrow is correlated with an increase in burden of driver lesions in a dose-dependent manner. Further, longer telomere length in tumors and mutations in telomere-maintenance genes indicate a potential role for telomere biology in the genesis of radiation-associated CLL. No other mutations were found in genes clinically associated with chemorefractoriness or affecting survival. Future analyses of larger patient sets in radiation-associated cancer types can help bolster these findings. To our knowledge, this is the first study to perform an in-depth genomic characterization of CLL in Chornobyl cleanup workers, highlighting a potentially important role for telomere biology in leukemogenesis.

## Additional file

Additional file 1: **Table S1.** List of target genes sequenced by targeted deep sequencing. **Table S2.** Mutations in Exposed and Unexposed Cases. **Table S3.** Copy Number Aberrations (CNA) in Exposed and Unexposed Cases. (DOCX 311 kb)

## Abbreviations

A-bomb: Atomic bomb
ALT: Alternative lengthening of telomeres
ANOVA: One-way analysis of variance
BWA: Burrows-Wheeler Aligner
CADD: Combined annotation dependent depletion
CLL: Chronic lymphocytic leukemia
CNA: Copy number abnormalities
ExAC: Exome aggregation consortium
GATK: Genome analysis toolkit
INDEL: Insertion and deletion
IR: Ionizing radiation
mTRF: Mean terminal restriction fragments
NMF: Non-negative matrix factorization
POT1: Protection of Telomeres 1
SNVs: Single nucleotide variants
TDS: Targeted deep sequencing
UN-CLL: Ukrainian non-irradiated patients with chronic lymphocytic leukemia
UR-CLL: Ukrainian Chornobyl Cleanup Workers with chronic lymphocytic leukemia
W- CLL: Western patients with chronic lymphocytic leukemia

## Footnotes

1 The date of diagnosis for each individual is the first date recorded when the absolute number of lymphocytes in the peripheral blood exceeded 5000/μL.

2 All cleanup workers registered in the State Chornobyl Registry were eligible for an annual health examination, which included a differential blood count and consultation with a physician. These examinations usually were conducted at a regional health facility.
