# Supplementary material for: Genomic characterization of chronic lymphocytic leukemia (CLL) in radiation-exposed Chornobyl cleanup workers
Source: Environ Health. 2018 May 2;17:43. doi: 10.1186/s12940-018-0387-9 (PMC5930419; doi:10.1186/s12940-018-0387-9)
Supplement: Supplementary file 1 — Table S1. List of target genes sequenced by targeted deep sequencing. Table S2. Mutations in Exposed and Unexposed Cases. Table S3. Copy Number Aberrations (CNA) in Exposed and Unexposed Cases. (DOCX 311 kb) [file 12940_2018_387_MOESM1_ESM.docx]

**Additional files**

**Genomic characterization of chronic lymphocytic leukemia (CLL) in radiation-exposed Chornobyl cleanup workers.**

**by Ojha J, Dyagil I, Finch SC, Reiss RF, deSmith AJ, Gonseth S, Zhou M, Hansen HM, Sherborne AL, Nakamura J, Bracci PM, Gudzenko N, Hatch M, Babkina N, Little MP, Chumak VV, Walsh KM, Bazyka D, Wiemels JL, Zablotska LB, 2017.**

| **Table S1: List of target genes sequenced by targeted deep sequencing** |
| --- |
| ABCB1 |
| ABL1 |
| ABL2 |
| ACVR1 |
| ACVR1B |
| ACVR2A |
| AJUBA |
| AKT1 |
| AKT2 |
| AKT3 |
| ALDH7A1 |
| ALK |
| APC |
| APOBEC3G |
| AR |
| ARAF |
| ARFRP1 |
| ARHGAP35 |
| ARID1A |
| ARID1B |
| ARID2 |
| ARID5B |
| ASH2L |
| ASXL1 |
| ASXL2 |
| ATF1 |
| ATM |
| ATR |
| ATRX |
| ATXN1 |
| AURKA |
| AURKB |
| AXIN1 |
| AXIN2 |
| AXL |
| BAP1 |
| BARD1 |
| BCL2 |
| BCL2A1 |
| BCL2L12 |
| BCL2L2 |
| BCL6 |
| BCL10 |
| BCL11B |
| BCOR |
| BCORL1 |
| BIRC3 |
| BLM |
| BRAF |
| BRCA1 |
| BRCA2 |
| BRD4 |
| BRIP1 |
| BTK |
| B4GALT3 |
| CALR |
| CARD11 |
| CBFB |
| CBL |
| CCND1 |
| CCND2 |
| CCND3 |
| CCNE1 |
| CD163L1 |
| CD79A |
| CD79B |
| CD274 |
| CDC42 |
| CDC73 |
| CDH1 |
| CDH2 |
| CDH4 |
| CDH5 |
| CDH20 |
| CDK12 |
| CDK4 |
| CDK6 |
| CDK8 |
| CDKN1A |
| CDKN1B |
| CDKN2A |
| CDKN2B |
| CDKN2C |
| CEBPA |
| CHD4 |
| CHEK1 |
| CHEK2 |
| CIC |
| CKS1B |
| CLDN18 |
| COL1A1 |
| COL2A1 |
| CREBBP |
| CRIPAK |
| CRKL |
| CRLF2 |
| CSF1R |
| CSF3R |
| CTCF |
| CTNNA1 |
| CTNNB1 |
| CUL3 |
| CUX1 |
| CXCR4 |
| CYLD |
| CYP2C8 |
| CYP2D6 |
| CYP3A4 |
| CYP3A5 |
| DAXX |
| DCC |
| DDIT3 |
| DDR2 |
| DDX3X |
| DICER1 |
| DNMT3A |
| DOT1L |
| DPYD |
| DUSP2 |
| DUSP4 |
| DUSP6 |
| DYNC1I1 |
| EBF1 |
| EDNRB |
| EGFR |
| EGR3 |
| EIFA2 |
| EIF1AX |
| ELF3 |
| EMSY  (C11orf30) |
| EP300 |
| EPCAM |
| EPHA3 |
| EPHA5 |
| EPHA6 |
| EPHA7 |
| EPHB1 |
| EPHB4 |
| EPHB6 |
| EPPK1 |
| ERBB2 |
| ERBB3 |
| ERBB4 |
| ERCC2 |
| ERCC6L |
| ERG |
| ESPL1 |
| ESR1 |
| ESR2 |
| ETS1 |
| ETV6 |
| EWSR1 |
| EZH1 |
| EZH2 |
| FAM123B (WTX) |
| FAM46C |
| FAM58A |
| FANCA |
| FANCC |
| FANCD2 |
| FANCE |
| FANCF |
| FANCG |
| FANCL |
| FBXO43 |
| FBXW7 |
| FGF10 |
| FGF14 |
| FGF19 |
| FGF23 |
| FGF3 |
| FGF4 |
| FGF6 |
| FGFR1 |
| FGFR2 |
| FGFR3 |
| FGFR4 |
| FH |
| FHIT |
| FKBP9 |
| FLCN |
| FLT1 |
| FLT3 |
| FLT4 |
| FOXA1 |
| FOXA2 |
| FOXL2 |
| FOXO1 |
| FOXO3 |
| FOXP1 |
| FOXP4 |
| FSIP1 |
| FUBP1 |
| FUS |
| FYN |
| GAB2 |
| GAK |
| GATA1 |
| GATA2 |
| GATA3 |
| GID4  (C17orf39) |
| GIGYF2 |
| GIPC3 |
| GLI1 |
| GLI2 |
| GNA11 |
| GNA13 |
| GNAQ |
| GNAS |
| GPR124 |
| GPSM1 |
| GRIN2A |
| GRM3 |
| GSK3A |
| GSK3B |
| GSTP1 |
| GTF2I |
| GUCY1A2 |
| H3F3A |
| H3F3B |
| H3F3C |
| HDAC9 |
| HGF |
| HIF1A |
| HIST1H1C |
| HIST1H3B |
| HLAA |
| HNF1A |
| HOXA3 |
| HOXB13 |
| HRAS |
| HSPA2 |
| HSPA5 |
| HSP90AA1 |
| ICK |
| ID3 |
| IDH1 |
| IDH2 |
| IGF1R |
| IGF2R |
| IKBKE |
| IKZF1 |
| IKZF2 |
| IKZF3 |
| IL36A |
| IL7R |
| INHBA |
| INSR |
| IRF4 |
| IRS2 |
| JAK1 |
| JAK2 |
| JAK3 |
| JAZF1 |
| JUN |
| KAT6A (MYST3) |
| KDM5A |
| KDM5C |
| KDM6A |
| KDR |
| KEAP1 |
| KIT |
| KLF4 |
| KLHL6 |
| KMT2A |
| KMT2B |
| KMT2C |
| KMT2D |
| KRAS |
| LARS |
| LEF1 |
| LIFR |
| LRP1B |
| LRP6 |
| LRRK2 |
| LTK |
| MAF |
| MAFB |
| MALAT1 |
| MAP2K1 |
| MAP2K2 |
| MAP2K4 |
| MAP3K1 |
| MAP3K2 |
| MAP3K5 |
| MAP3K7 |
| MAP3K9 |
| MAPK1 |
| MAPK8IP1 |
| MCL1 |
| MCTP1 |
| MDM2 |
| MDM4 |
| MECOM |
| MED12 |
| MEF2B |
| MEN1 |
| MET |
| MGA |
| MIR142 |
| MITF |
| MLH1 |
| MPL |
| MRAS |
| MRE11A |
| MSH2 |
| MSH6 |
| MTOR |
| MUTYH |
| MYB |
| MYBL1 |
| MYC |
| MYCL |
| MYCN |
| MYD88 |
| MYH9 |
| NAV3 |
| NBN |
| NCOR1 |
| NF1 |
| NF2 |
| NFE2L3 |
| NFE2L2 |
| NFKBIA |
| NFKBIE |
| NIPBL |
| NKX2-­‐1 |
| NOTCH1 |
| NOTCH2 |
| NOTCH3 |
| NOTCH4 |
| NPM1 |
| NRAS |
| NSD1 |
| NT5C2 |
| NTRK1 |
| NTRK2 |
| NTRK3 |
| NUP93 |
| NUP98 |
| NUTM1 |
| OR5L1 |
| PAK3 |
| PALB2 |
| PAX3 |
| PAX5 |
| PAX8 |
| PBRM1 |
| PCBP1 |
| PDCD1LG2 |
| PDGFB |
| PDGFRA |
| PDGFRB |
| PDK1 |
| PDPK1 |
| PDS5B |
| PHF6 |
| PHLPP2 |
| PIK3CA |
| PIK3CG |
| PIK3R1 |
| PIK3R2 |
| PIM1 |
| PKHD1 |
| PLCG1 |
| PLCH2 |
| PMS2 |
| POLD1 |
| POLE |
| POLQ |
| POT1 |
| POU3F2 |
| PPM1D |
| PPP2R1A |
| PPP6C |
| PRDM1 |
| PRKACA |
| PRKAR1A |
| PRKCA |
| PRKDC |
| PRSS1 |
| PRX |
| PTCH1 |
| PTCH2 |
| PTEN |
| PTK2B |
| PTPN1 |
| PTPN11 |
| PTPRD |
| PTPRK |
| PTPRT |
| PYDC2 |
| RAC1 |
| RAC2 |
| RAD21 |
| RAD50 |
| RAD51 |
| RAD51C |
| RAD51D |
| RAF1 |
| RARA |
| RASA1 |
| RB1 |
| RBM10 |
| REL |
| RELA |
| RET |
| RHEB |
| RHOA |
| RHOT1 |
| RICTOR |
| RIT1 |
| RNF43 |
| ROS1 |
| RPL5 |
| RPL22 |
| RPTOR |
| RRAS |
| RRAS2 |
| RUNX1 |
| RUNX1T1 |
| SDHA |
| SDHB |
| SDHC |
| SDHD |
| SETBP1 |
| SETD2 |
| SF3B1 |
| SGK1 |
| SH2B3 |
| SHH |
| SHOC2 |
| SIN3A |
| SLITRK6 |
| SMAD2 |
| SMAD3 |
| SMAD4 |
| SMARCA2 |
| SMARCA4 |
| SMARCB1 |
| SMC1A |
| SMC3 |
| SMG7 |
| SMO |
| SNX31 |
| SOCS1 |
| SOS1 |
| SOS2 |
| SOX9 |
| SOX10 |
| SOX17 |
| SOX2 |
| SPEN |
| SPOP |
| SPRED1 |
| SPRY1 |
| SPRY2 |
| SPRY4 |
| SPTA1 |
| SRC |
| SRSF2 |
| SS18 |
| STAG1 |
| STAG2 |
| STAT3 |
| STAT4 |
| STAT5A |
| STAT5B |
| STAT6 |
| STK11 |
| STK19 |
| SUFU |
| SUZ12 |
| TACC1 |
| TADA1 |
| TADA2B |
| TAF1 |
| TAS2R60 |
| TBL1XR1 |
| TBX3 |
| TBX22 |
| TCF3 |
| TCF4 |
| TCF7L2 |
| TCL1A |
| TERT |
| TET2 |
| TFE3 |
| TFEB |
| TGFBR2 |
| TLR4 |
| TNC |
| TNFAIP3 |
| TNFRSF14 |
| TNKS |
| TNKS2 |
| TOP1 |
| TMPRSS2 |
| TP53 |
| TP63 |
| TPMT |
| TRAF7 |
| TRPM1 |
| TRPM3 |
| TRRAP |
| TSC1 |
| TSC2 |
| TSHR |
| TSHZ2 |
| TSHZ3 |
| TSPY1 |
| U2AF1 |
| UGT1A1 |
| UGT1A7 |
| USP6 |
| USP7 |
| USP9X |
| VAT1L |
| VEZF1 |
| VHL |
| WAPAL |
| WHSC1 |
| WISP3 |
| WRN |
| WT1 |
| XPO1 |
| XRCC2 |
| XRCC3 |
| XRCC5 |
| XRCC6 |
| YAP1 |
| YWHAE |
| ZFHX3 |
| ZMYM3 |
| ZNF217 |
| ZNF668 |
| ZNF703 |
| ZRSR2 |

**Table S2: Mutations in Exposed and Unexposed Cases.**

| **Patient ID#** |  | **Chr** | **Start** | **End** | **Ref** | **Alt** | **Function** | **Gene** | **Transcript ID** | **Nucleotide Change** | **AAChange** |
| --- | --- | --- | --- | --- | --- | --- | --- | --- | --- | --- | --- |
| E01 | CGP-251 | chr1 | 23885852 | 23885852 | C | G | synonymous SNV | ID3 | NM_002167 | c.G66C | p.L22L |
| E01 | CGP-251 | chr13 | 49033845 | 49033845 | G | C | nonsynonymous SNV | RB1 | NM_000321 | c.G1982C | p.R661P |
| E01 | CGP-251 | chr15 | 41803521 | 41803521 | G | A | nonsynonymous SNV | LTK | NM_002344 | c.C838T | p.R280W |
| E01 | CGP-251 | chr3 | 47058584 | 47058584 | C | T | synonymous SNV | SETD2 | NM_014159 | c.G7694A | p.X2565X |
| E01 | CGP-251 | chr6 | 128403602 | 128403602 | T | C | nonsynonymous SNV | PTPRK | NM_001291983 | c.A1370G | p.N457S |
| E02 | CGP-252 | chr12 | 6711608 | 6711608 | C | T | synonymous SNV | CHD4 | NM_001297553 | c.G156A | p.K52K |
| E02 | CGP-252 | chr16 | 89858464 | 89858464 | G | A | synonymous SNV | FANCA | NM_000135 | c.C1096T | p.L366L |
| E02 | CGP-252 | chr22 | 37637661 | 37637661 | T | C | nonsynonymous SNV | RAC2 | NM_002872 | c.A73G | p.T25A |
| E02 | CGP-252 | chr4 | 153249510 | 153249510 | C | A | nonsynonymous SNV | FBXW7 | NM_001013415 | c.G914T | p.G305V |
| E02 | CGP-252 | chr7 | 124503471 | 124503471 | T | A | nonsynonymous SNV | POT1 | NM_001042594 | c.A86T | p.Q29L |
| E02 | CGP-252 | chr8 | 144946503 | 144946503 | C | T | nonsynonymous SNV | EPPK1 | NM_031308 | c.G919A | p.E307K |
| E02 | CGP-252 | chr8 | 27277435 | 27277435 | G | A | synonymous SNV | PTK2B | NM_173175 | c.G228A | p.L76L |
| E03 | CGP-253 | chr16 | 2114425 | 2114425 | G | A | synonymous SNV | TSC2 | NM_000548 | c.G1596A | p.E532E |
| E03 | CGP-253 | chr16 | 9857069 | 9857069 | A | T | synonymous SNV | GRIN2A | NM_001134407 | c.T4332A | p.V1444V |
| E03 | CGP-253 | chr17 | 38504619 | 38504619 | C | T | nonsynonymous SNV | RARA | NM_001024809 | c.C215T | p.S72L |
| E03 | CGP-253 | chr21 | 44514658 | 44514658 | G | A | synonymous SNV | U2AF1 | NM_001025203 | c.C498T | p.G166G |
| E03 | CGP-253 | chr22 | 41574341 | 41574352 | ACCAGTTCCAGC | - | nonframeshift deletion | EP300 | NM_001429 | c.6626_6637del | p.2209_2213del |
| E03 | CGP-253 | chr22 | 29678532 | 29678532 | C | G | synonymous SNV | EWSR1 | NM_001163285 | c.C567G | p.S189S |
| E03 | CGP-253 | chr3 | 138664891 | 138664893 | GCT | - | nonframeshift deletion | FOXL2 | NM_023067 | c.672_674del | p.224_225del |
| E03 | CGP-253 | chr7 | 151856098 | 151856098 | T | A | synonymous SNV | KMT2C | NM_170606 | c.A11520T | p.T3840T |
| E03 | CGP-253 | chrX | 70338701 | 70338701 | G | A | nonsynonymous SNV | MED12 | NM_005120 | c.G97A | p.E33K |
| E04 | CGP-254 | chr12 | 49445445 | 49445445 | G | A | nonsynonymous SNV | KMT2D | NM_003482 | c.C2021T | p.P674L |
| E04 | CGP-254 | chr13 | 32912964 | 32912964 | T | C | nonsynonymous SNV | BRCA2 | NM_000059 | c.T4472C | p.L1491P |
| E04 | CGP-254 | chr16 | 71715748 | 71715748 | C | T | nonsynonymous SNV | PHLPP2 | NM_001289003 | c.G796A | p.E266K |
| E04 | CGP-254 | chr17 | 17131410 | 17131410 | G | A | synonymous SNV | FLCN | NM_144606 | c.C42T | p.H14H |
| E04 | CGP-254 | chr2 | 136872487 | 136872488 | AT | - | frameshift deletion | CXCR4 | NM_001008540 | c.1022_1023del | p.H341fs |
| E04 | CGP-254 | chr20 | 51871266 | 51871266 | G | A | synonymous SNV | TSHZ2 | NM_001193421 | c.G1260A | p.P420P |
| E04 | CGP-254 | chr3 | 10085546 | 10085546 | A | G | nonsynonymous SNV | FANCD2 | NM_001018115 | c.A1132G | p.K378E |
| E04 | CGP-254 | chr3 | 70008503 | 70008503 | G | A | nonsynonymous SNV | MITF | NM_000248 | c.G790A | p.A264T |
| E04 | CGP-254 | chr6 | 160490922 | 160490922 | C | T | synonymous SNV | IGF2R | NM_000876 | c.C4275T | p.A1425A |
| E05 | CGP-255 | chr11 | 108205715 | 108205715 | A | T | nonsynonymous SNV | ATM | NM_000051 | c.A8030T | p.Y2677F |
| E05 | CGP-255 | chr16 | 72993397 | 72993397 | C | T | synonymous SNV | ZFHX3 | NM_006885 | c.G648A | p.P216P |
| E05 | CGP-255 | chr19 | 36211454 | 36211454 | C | T | nonsynonymous SNV | KMT2B | NM_014727 | c.C1205T | p.P402L |
| E05 | CGP-255 | chr19 | 45867776 | 45867776 | G | A | synonymous SNV | ERCC2 | NM_001130867 | c.C552T | p.S184S |
| E05 | CGP-255 | chr2 | 25973004 | 25973004 | A | G | nonsynonymous SNV | ASXL2 | NM_018263 | c.T1421C | p.L474P |
| E05 | CGP-255 | chr22 | 36692984 | 36692984 | G | T | nonsynonymous SNV | MYH9 | NM_002473 | c.C3177A | p.D1059E |
| E05 | CGP-255 | chr5 | 56168735 | 56168735 | G | A | nonsynonymous SNV | MAP3K1 | NM_005921 | c.G1589A | p.G530E |
| E05 | CGP-255 | chr7 | 87179567 | 87179567 | C | T | nonsynonymous SNV | ABCB1 | NM_000927 | c.G1270A | p.A424T |
| E05 | CGP-255 | chr7 | 151884840 | 151884840 | T | G | nonsynonymous SNV | KMT2C | NM_170606 | c.A4753C | p.T1585P |
| E05 | CGP-255 | chr9 | 21974674 | 21974674 | C | G | synonymous SNV | CDKN2A | NM_058197 | c.G153C | p.V51V |
| E06 | CGP-256 | chr14 | 99641196 | 99641196 | C | G | synonymous SNV | BCL11B | NM_001282238 | c.G1761C | p.A587A |
| E06 | CGP-256 | chr16 | 72991478 | 72991478 | G | A | nonsynonymous SNV | ZFHX3 | NM_006885 | c.C2567T | p.P856L |
| E06 | CGP-256 | chr17 | 40447807 | 40447807 | C | T | synonymous SNV | STAT5A | NM_001288719 | c.C456T | p.I152I |
| E06 | CGP-256 | chr2 | 121743855 | 121743855 | T | C | nonsynonymous SNV | GLI2 | NM_005270 | c.T1958C | p.L653P |
| E06 | CGP-256 | chr5 | 38960561 | 38960561 | T | C | nonsynonymous SNV | RICTOR | NM_001285439 | c.A1790G | p.K597R |
| E06 | CGP-256 | chr9 | 21974674 | 21974674 | C | G | synonymous SNV | CDKN2A | NM_058197 | c.G153C | p.V51V |
| E08 | CGP-446 | chr1 | 11188183 | 11188183 | C | T | nonsynonymous SNV | MTOR | NM_004958 | c.G5911A | p.A1971T |
| E08 | CGP-446 | chr11 | 108218084 | 108218084 | T | C | nonsynonymous SNV | ATM | NM_000051 | c.T8663C | p.I2888T |
| E08 | CGP-446 | chr13 | 80911389 | 80911389 | C | T | nonsynonymous SNV | SPRY2 | NM_005842 | c.G452A | p.R151Q |
| E08 | CGP-446 | chr13 | 49039188 | 49039189 | TA | - | frameshift deletion | RB1 | NM_000321 | c.2266_2267del | p.Y756fs |
| E08 | CGP-446 | chr17 | 63530081 | 63530081 | C | T | nonsynonymous SNV | AXIN2 | NM_004655 | c.G2354A | p.S785N |
| E08 | CGP-446 | chr2 | 61145720 | 61145720 | A | G | nonsynonymous SNV | REL | NM_001291746 | c.A832G | p.R278G |
| E08 | CGP-446 | chr2 | 61719472 | 61719472 | C | T | nonsynonymous SNV | XPO1 | NM_003400 | c.G1711A | p.E571K |
| E08 | CGP-446 | chr2 | 141294278 | 141294278 | G | C | nonsynonymous SNV | LRP1B | NM_018557 | c.C7514G | p.T2505S |
| E08 | CGP-446 | chr5 | 1294976 | 1294976 | G | A | synonymous SNV | TERT | NM_001193376 | c.C129T | p.D43D |
| E08 | CGP-446 | chr8 | 27289846 | 27289846 | G | A | nonsynonymous SNV | PTK2B | NM_173175 | c.G955A | p.V319I |
| E08 | CGP-446 | chr8 | 41791244 | 41791244 | G | A | synonymous SNV | KAT6A | NM_006766 | c.C4494T | p.S1498S |
| E10 | CGP-447 | chr1 | 166829552 | 166829552 | G | A | nonsynonymous SNV | TADA1 | NM_053053 | c.C563T | p.T188M |
| E10 | CGP-447 | chr15 | 67358522 | 67358522 | G | A | synonymous SNV | SMAD3 | NM_005902 | c.G30A | p.P10P |
| E10 | CGP-447 | chr17 | 7577568 | 7577568 | C | T | nonsynonymous SNV | TP53 | NM_001126115 | c.G317A | p.C106Y |
| E10 | CGP-447 | chr2 | 198266489 | 198266489 | C | T | nonsynonymous SNV | SF3B1 | NM_012433 | c.G2347A | p.E783K |
| E10 | CGP-447 | chr3 | 37070316 | 37070316 | A | G | nonsynonymous SNV | MLH1 | NM_001167619 | c.A728G | p.D243G |
| E10 | CGP-447 | chr6 | 44233437 | 44233437 | C | T | nonsynonymous SNV | NFKBIE | NM_004556 | c.G64A | p.G22R |
| E10 | CGP-447 | chr7 | 124487011 | 124487011 | G | C | nonsynonymous SNV | POT1 | NM_001042594 | c.C598G | p.Q200E |
| E10 | CGP-447 | chr9 | 133759774 | 133759774 | C | T | synonymous SNV | ABL1 | NM_005157 | c.C2097T | p.A699A |
| E10 | CGP-447 | chrX | 129146595 | 129146595 | G | C | nonsynonymous SNV | BCORL1 | NM_021946 | c.G128C | p.C43S |
| E10 | CGP-447 | chrX | 41202564 | 41202565 | TA | - | frameshift deletion | DDX3X | NM_001193417 | c.591_592del | p.I197fs |
| E13 | CGP-448 | chr11 | 102207494 | 102207494 | A | G | nonsynonymous SNV | BIRC3 | NM_001165 | c.A1583G | p.Q528R |
| E13 | CGP-448 | chr12 | 111856077 | 111856077 | G | T | nonsynonymous SNV | SH2B3 | NM_005475 | c.G128T | p.R43L |
| E13 | CGP-448 | chr4 | 1389573 | 1389573 | G | C | nonsynonymous SNV | CRIPAK | NM_175918 | c.G1274C | p.G425A |
| E14 | CGP-449 | chr12 | 58144515 | 58144515 | G | A | nonsynonymous SNV | CDK4 | NM_000075 | c.C556T | p.L186F |
| E14 | CGP-449 | chr12 | 49415566 | 49415566 | G | C | nonsynonymous SNV | KMT2D | NM_003482 | c.C16611G | p.N5537K |
| E14 | CGP-449 | chr17 | 70120088 | 70120088 | G | A | nonsynonymous SNV | SOX9 | NM_000346 | c.G1090A | p.A364T |
| E14 | CGP-449 | chr17 | 78938086 | 78938086 | G | A | nonsynonymous SNV | RPTOR | NM_001163034 | c.G3490A | p.D1164N |
| E14 | CGP-449 | chr19 | 33792919 | 33792919 | C | T | synonymous SNV | CEBPA | NM_001285829 | c.G45A | p.A15A |
| E14 | CGP-449 | chr5 | 149499633 | 149499633 | G | - | stopgain | PDGFRB | NM_002609 | c.2640delC | p.Y880X |
| E14 | CGP-449 | chr6 | 37138626 | 37138626 | T | A | nonsynonymous SNV | PIM1 | NM_001243186 | c.T433A | p.S145T |
| E14 | CGP-449 | chr6 | 138200203 | 138200203 | A | T | stopgain | TNFAIP3 | NM_001270507 | c.A1621T | p.K541X |
| E14 | CGP-449 | chr7 | 124532344 | 124532344 | G | A | nonsynonymous SNV | POT1 | NM_015450 | c.C100T | p.P34S |
| E14 | CGP-449 | chr7 | 151860169 | 151860169 | G | A | nonsynonymous SNV | KMT2C | NM_170606 | c.C10493T | p.T3498I |
| E16 | CGP-451 | chr16 | 2627491 | 2627491 | G | A | nonsynonymous SNV | PDPK1 | NM_031268 | c.G394A | p.A132T |
| E16 | CGP-451 | chr17 | 37947776 | 37947776 | A | C | nonsynonymous SNV | IKZF3 | NM_001257408 | c.T383G | p.L128R |
| E16 | CGP-451 | chr2 | 198267491 | 198267491 | C | A | nonsynonymous SNV | SF3B1 | NM_012433 | c.G1866T | p.E622D |
| E16 | CGP-451 | chr2 | 233633259 | 233633259 | G | A | nonsynonymous SNV | KCNJ13 | NM_001172417 | c.C485T | p.T162M |
| E16 | CGP-451 | chr3 | 176744213 | 176744213 | A | C | nonsynonymous SNV | TBL1XR1 | NM_024665 | c.T1466G | p.V489G |
| E16 | CGP-451 | chr5 | 94620236 | 94620236 | G | A | nonsynonymous SNV | MCTP1 | NM_024717 | c.C44T | p.A15V |
| E18 | CGP-452 | chr16 | 2114425 | 2114425 | G | A | synonymous SNV | TSC2 | NM_000548 | c.G1596A | p.E532E |
| E18 | CGP-452 | chr21 | 44514658 | 44514658 | G | A | synonymous SNV | U2AF1 | NM_001025203 | c.C498T | p.G166G |
| E18 | CGP-452 | chr22 | 29678532 | 29678532 | C | G | synonymous SNV | EWSR1 | NM_001163285 | c.C567G | p.S189S |
| E18 | CGP-452 | chr7 | 151856098 | 151856098 | T | A | synonymous SNV | KMT2C | NM_170606 | c.A11520T | p.T3840T |
| E18 | CGP-452 | chrX | 70338701 | 70338701 | G | A | nonsynonymous SNV | MED12 | NM_005120 | c.G97A | p.E33K |
| E19 | CGP-453 | chr13 | 41134420 | 41134420 | G | A | nonsynonymous SNV | FOXO1 | NM_002015 | c.C1208T | p.P403L |
| E19 | CGP-453 | chr17 | 33434141 | 33434141 | C | T | nonsynonymous SNV | RAD51D | NM_001142571 | c.G406A | p.V136I |
| E19 | CGP-453 | chr22 | 42046885 | 42046885 | G | A | synonymous SNV | XRCC6 | NM_001288978 | c.G969A | p.S323S |
| E19 | CGP-453 | chr3 | 38182025 | 38182025 | G | T | nonsynonymous SNV | MYD88 | NM_001172568 | c.G514T | p.V172F |
| E19 | CGP-453 | chr7 | 124503692 | 124503692 | - | AGG | nonframeshift insertion | POT1 | NM_015450 | c.257_258insCCT | p.I86delinsIL |
| E20 | CGP-454 | chr16 | 23647289 | 23647289 | G | A | nonsynonymous SNV | PALB2 | NM_024675 | c.C578T | p.T193I |
| E20 | CGP-454 | chr2 | 39249849 | 39249849 | C | T | nonsynonymous SNV | SOS1 | NM_005633 | c.G1720A | p.V574I |
| E20 | CGP-454 | chr4 | 124323501 | 124323501 | - | CTG | nonframeshift insertion | SPRY1 | NM_001258039 | c.755_756insCTG | p.H252delinsHC |
| E20 | CGP-454 | chr5 | 37060949 | 37060949 | A | G | synonymous SNV | NIPBL | NM_015384 | c.A7689G | p.K2563K |
| E20 | CGP-454 | chr5 | 138163236 | 138163236 | C | T | synonymous SNV | CTNNA1 | NM_001290309 | c.C582T | p.S194S |
| E20 | CGP-454 | chr9 | 139412642 | 139412642 | G | C | nonsynonymous SNV | NOTCH1 | NM_017617 | c.C1202G | p.P401R |
| E20 | CGP-454 | chr9 | 139390649 | 139390650 | AG | - | frameshift deletion | NOTCH1 | NM_017617 | c.7541_7542del | p.P2514fs |
| E24 | CGP-455 | chr12 | 49432550 | 49432550 | G | A | synonymous SNV | KMT2D | NM_003482 | c.C8589T | p.G2863G |
| E24 | CGP-455 | chr19 | 36209187 | 36209187 | C | A | synonymous SNV | KMT2B | NM_014727 | c.C267A | p.V89V |
| E24 | CGP-455 | chr21 | 36164468 | 36164468 | G | C | synonymous SNV | RUNX1 | NM_001001890 | c.C1326G | p.S442S |
| E24 | CGP-455 | chr9 | 139390649 | 139390650 | AG | - | frameshift deletion | NOTCH1 | NM_017617 | c.7541_7542del | p.P2514fs |
| E25 | CGP-456 | chr12 | 7521563 | 7521563 | - | GAGA | . | . |  |  |  |
| E25 | CGP-456 | chr19 | 17941001 | 17941001 | C | G | nonsynonymous SNV | JAK3 | NM_000215 | c.G3123C | p.E1041D |
| E25 | CGP-456 | chr19 | 15285103 | 15285103 | C | A | synonymous SNV | NOTCH3 | NM_000435 | c.G4512T | p.V1504V |
| E25 | CGP-456 | chr20 | 31022982 | 31022982 | T | - | stopgain | ASXL1 | NM_015338 | c.2467delT | p.L823X |
| E25 | CGP-456 | chr5 | 112157678 | 112157678 | G | A | nonsynonymous SNV | APC | NM_001127511 | c.G1344A | p.M448I |
| E29 | CGP-457 | chr13 | 28608250 | 28608250 | T | C | synonymous SNV | FLT3 | NM_004119 | c.A1806G | p.K602K |
| E29 | CGP-457 | chr14 | 23777174 | 23777174 | T | C | synonymous SNV | BCL2L2 | NM_001199839 | c.T198C | p.A66A |
| E29 | CGP-457 | chr16 | 72828257 | 72828257 | G | A | nonsynonymous SNV | ZFHX3 | NM_001164766 | c.C5582T | p.P1861L |
| E29 | CGP-457 | chr17 | 48277121 | 48277121 | G | T | nonsynonymous SNV | COL1A1 | NM_000088 | c.C291A | p.D97E |
| E29 | CGP-457 | chr18 | 59221658 | 59221658 | G | T | synonymous SNV | CDH20 | NM_031891 | c.G2136T | p.V712V |
| E29 | CGP-457 | chr18 | 59221790 | 59221790 | G | T | synonymous SNV | CDH20 | NM_031891 | c.G2268T | p.V756V |
| E29 | CGP-457 | chr19 | 15288391 | 15288391 | C | A | nonsynonymous SNV | NOTCH3 | NM_000435 | c.G4348T | p.A1450S |
| E29 | CGP-457 | chr19 | 15299826 | 15299826 | C | A | nonsynonymous SNV | NOTCH3 | NM_000435 | c.G1352T | p.G451V |
| E29 | CGP-457 | chr19 | 40902215 | 40902215 | C | T | nonsynonymous SNV | PRX | NM_181882 | c.G2044A | p.E682K |
| E29 | CGP-457 | chr3 | 96533835 | 96533835 | A | T | nonsynonymous SNV | EPHA6 | NM_001080448 | c.A368T | p.H123L |
| E29 | CGP-457 | chr4 | 1962801 | 1962801 | G | A | nonsynonymous SNV | WHSC1 | NM_001042424 | c.G3295A | p.E1099K |
| E29 | CGP-457 | chr5 | 112090711 | 112090711 | T | G | nonsynonymous SNV | APC | NM_000038 | c.T124G | p.S42A |
| U02 | CGP-258 | chr1 | 16261470 | 16261470 | C | T | nonsynonymous SNV | SPEN | NM_015001 | c.C8735T | p.P2912L |
| U02 | CGP-258 | chr19 | 1619835 | 1619835 | G | C | nonsynonymous SNV | TCF3 | NM_001136139 | c.C1111G | p.R371G |
| U02 | CGP-409 | chr20 | 51871137 | 51871137 | A | G | synonymous SNV | TSHZ2 | NM_001193421 | c.A1131G | p.L377L |
| U02 | CGP-258 | chr3 | 128199914 | 128199914 | C | A | nonsynonymous SNV | GATA2 | NM_001145662 | c.G1349T | p.S450I |
| U02 | CGP-258 | chr3 | 183273187 | 183273187 | G | C | nonsynonymous SNV | KLHL6 | NM_130446 | c.C255G | p.C85W |
| U02 | CGP-409 | chr3 | 183209717 | 183209718 | AG | - | frameshift deletion | KLHL6 | NM_130446 | c.1863_1864del | p.V621fs |
| U02 | CGP-409 | chr5 | 180050961 | 180050961 | T | A | nonsynonymous SNV | FLT4 | NM_002020 | c.A1522T | p.T508S |
| U03 | CGP-259 | chr15 | 31334274 | 31334274 | C | T | nonsynonymous SNV | TRPM1 | NM_001252020 | c.G2018A | p.R673Q |
| U03 | CGP-259 | chr17 | 17125907 | 17125907 | G | A | synonymous SNV | FLCN | NM_144606 | c.C687T | p.F229F |
| U03 | CGP-259 | chr17 | 56436108 | 56436108 | G | A | synonymous SNV | RNF43 | NM_017763 | c.C1029T | p.R343R |
| U03 | CGP-259 | chr17 | 56798133 | 56798133 | A | G | synonymous SNV | RAD51C | NM_058216 | c.A864G | p.T288T |
| U03 | CGP-259 | chr19 | 1206943 | 1206943 | A | T | nonsynonymous SNV | STK11 | NM_000455 | c.A31T | p.M11L |
| U03 | CGP-259 | chr19 | 30313198 | 30313198 | A | G | nonsynonymous SNV | CCNE1 | NM_001238 | c.A892G | p.I298V |
| U03 | CGP-259 | chr22 | 36710237 | 36710237 | C | T | nonsynonymous SNV | MYH9 | NM_002473 | c.G1507A | p.D503N |
| U03 | CGP-259 | chr22 | 36680521 | 36680521 | G | A | synonymous SNV | MYH9 | NM_002473 | c.C5520T | p.T1840T |
| U03 | CGP-259 | chr3 | 10116263 | 10116263 | A | G | nonsynonymous SNV | FANCD2 | NM_001018115 | c.A2765G | p.H922R |
| U03 | CGP-259 | chr3 | 89176370 | 89176370 | G | T | nonsynonymous SNV | EPHA3 | NM_005233 | c.G100T | p.D34Y |
| U05 | CGP-459 | chr11 | 3697848 | 3697848 | C | T | synonymous SNV | NUP98 | NM_139132 | c.G4854A | p.V1618V |
| U05 | CGP-459 | chr22 | 41566519 | 41566519 | T | C | nonsynonymous SNV | EP300 | NM_001429 | c.T4396C | p.W1466R |
| U05 | CGP-459 | chr6 | 41557524 | 41557524 | G | A | nonsynonymous SNV | FOXP4 | NM_001012426 | c.G1081A | p.E361K |
| U05 | CGP-459 | chr7 | 2969673 | 2969673 | G | C | nonsynonymous SNV | CARD11 | NM_032415 | c.C1606G | p.P536A |
| U05 | CGP-459 | chr7 | 151856070 | 151856070 | G | A | stopgain | KMT2C | NM_170606 | c.C11548T | p.R3850X |
| U06 | CGP-460 | chr12 | 78598890 | 78598890 | C | T | nonsynonymous SNV | NAV3 | NM_014903 | c.C6944T | p.P2315L |
| U06 | CGP-460 | chr14 | 36989291 | 36989291 | G | A | nonsynonymous SNV | NKX2-1 | NM_001079668 | c.C44T | p.A15V |
| U06 | CGP-460 | chr17 | 78820280 | 78820280 | C | A | nonsynonymous SNV | RPTOR | NM_001163034 | c.C1220A | p.P407Q |
| U06 | CGP-460 | chr19 | 40762862 | 40762862 | A | T | nonsynonymous SNV | AKT2 | NM_001626 | c.T146A | p.L49Q |
| U06 | CGP-460 | chr19 | 33792732 | 33792737 | GCGGGT | - | nonframeshift deletion | CEBPA | NM_001285829 | c.227_232del | p.76_78del |
| U06 | CGP-460 | chr2 | 198265007 | 198265007 | C | T | nonsynonymous SNV | SF3B1 | NM_012433 | c.G2870A | p.R957Q |
| U06 | CGP-460 | chr3 | 136087952 | 136087952 | A | G | nonsynonymous SNV | STAG1 | NM_005862 | c.T2543C | p.M848T |
| U06 | CGP-460 | chr9 | 98220519 | 98220519 | G | A | nonsynonymous SNV | PTCH1 | NM_000264 | c.C2944T | p.R982W |
| U07 | CGP-461 | chr1 | 40367555 | 40367555 | G | T | stopgain | MYCL | NM_001033082 | c.C6A | p.C2X |
| U07 | CGP-461 | chr6 | 93967957 | 93967957 | T | A | nonsynonymous SNV | EPHA7 | NM_001288629 | c.A1955T | p.K652I |
| U07 | CGP-461 | chr7 | 86416163 | 86416163 | G | A | nonsynonymous SNV | GRM3 | NM_000840 | c.G1055A | p.R352Q |
| U07 | CGP-461 | chr7 | 116415129 | 116415129 | A | G | nonsynonymous SNV | MET | NM_000245 | c.A3223G | p.S1075G |
| U07 | CGP-461 | chr7 | 140624390 | 140624390 | A | G | synonymous SNV | BRAF | NM_004333 | c.T114C | p.A38A |
| U07 | CGP-461 | chr8 | 144940161 | 144940161 | A | T | stoploss | EPPK1 | NM_031308 | c.T7261A | p.X2421R |
| U08 | CGP-462 | chr1 | 2434345 | 2434345 | C | T | synonymous SNV | PLCH2 | NM_001303012 | c.C3375T | p.S1125S |
| U08 | CGP-462 | chr16 | 71682831 | 71682831 | G | A | nonsynonymous SNV | PHLPP2 | NM_001289003 | c.C3733T | p.R1245W |
| U08 | CGP-462 | chr19 | 30303622 | 30303622 | A | C | nonsynonymous SNV | CCNE1 | NM_001238 | c.A50C | p.K17T |
| U08 | CGP-462 | chr2 | 121746368 | 121746368 | G | A | nonsynonymous SNV | GLI2 | NM_005270 | c.G2878A | p.E960K |
| U09 | CGP-463 | chr1 | 17350546 | 17350546 | G | A | synonymous SNV | SDHB | NM_003000 | c.C564T | p.L188L |
| U09 | CGP-463 | chr12 | 111856110 | 111856110 | A | C | nonsynonymous SNV | SH2B3 | NM_005475 | c.A161C | p.Q54P |
| U09 | CGP-463 | chr15 | 34649086 | 34649088 | AGG | - | nonframeshift deletion | NUTM1 | NM_001284293 | c.2847_2849del | p.949_950del |
| U09 | CGP-463 | chr19 | 11107037 | 11107037 | A | G | nonsynonymous SNV | SMARCA4 | NM_001128845 | c.A1742G | p.K581R |
| U09 | CGP-463 | chr2 | 198267489 | 198267489 | T | C | nonsynonymous SNV | SF3B1 | NM_012433 | c.A1868G | p.Y623C |
| U09 | CGP-463 | chr5 | 56171112 | 56171112 | T | A | nonsynonymous SNV | MAP3K1 | NM_005921 | c.T1940A | p.V647D |
| U10 | CGP-464 | chr12 | 115112347 | 115112347 | C | A | nonsynonymous SNV | TBX3 | NM_005996 | c.G1333T | p.A445S |
| U10 | CGP-464 | chr16 | 3900684 | 3900684 | C | T | nonsynonymous SNV | CREBBP | NM_001079846 | c.G412A | p.A138T |
| U10 | CGP-464 | chr17 | 38499040 | 38499040 | G | T | nonsynonymous SNV | RARA | NM_001024809 | c.G84T | p.L28F |
| U10 | CGP-464 | chr2 | 30143268 | 30143268 | C | G | nonsynonymous SNV | ALK | NM_004304 | c.G258C | p.E86D |
| U10 | CGP-464 | chr2 | 198265476 | 198265476 | T | C | nonsynonymous SNV | SF3B1 | NM_012433 | c.A2681G | p.D894G |
| U10 | CGP-464 | chr2 | 225367738 | 225367738 | T | G | nonsynonymous SNV | CUL3 | NM_001257197 | c.A1231C | p.M411L |
| U10 | CGP-464 | chr2 | 225367736 | 225367736 | - | GA | frameshift insertion | CUL3 | NM_001257197 | c.1232_1233insTC | p.M411fs |
| U10 | CGP-464 | chr9 | 117836119 | 117836119 | C | G | nonsynonymous SNV | TNC | NM_002160 | c.G2977C | p.V993L |
| U10 | CGP-464 | chrX | 41204552 | 41204552 | G | C | nonsynonymous SNV | DDX3X | NM_001193417 | c.G1097C | p.S366T |
| U10 | CGP-464 | chrX | 47040962 | 47040962 | C | A | nonsynonymous SNV | RBM10 | NM_001204466 | c.C1261A | p.R421S |
| U11 | CGP-406 | chr14 | 50587065 | 50587065 | T | C | synonymous SNV | SOS2 | NM_006939 | c.A3417G | p.L1139L |
| U11 | CGP-406 | chr6 | 51890718 | 51890718 | G | A | nonsynonymous SNV | PKHD1 | NM_138694 | c.C3890T | p.A1297V |
| U11 | CGP-406 | chrX | 41077774 | 41077774 | T | C | nonsynonymous SNV | USP9X | NM_001039590 | c.T6359C | p.I2120T |
| U14 | CGP-336 | chr11 | 65425803 | 65425803 | G | T | synonymous SNV | RELA | NM_001145138 | c.C823A | p.R275R |
| U14 | CGP-336 | chr12 | 7527100 | 7527100 | G | C | nonsynonymous SNV | CD163L1 | NM_001297650 | c.C3377G | p.P1126R |
| U14 | CGP-336 | chr19 | 33793054 | 33793056 | CTC | - | nonframeshift deletion | CEBPA | NM_001287424 | c.370_372del | p.124_124del |
| U14 | CGP-336 | chr6 | 157099616 | 157099616 | G | C | nonsynonymous SNV | ARID1B | NM_017519 | c.G553C | p.E185Q |
| U14 | CGP-336 | chr6 | 44232739 | 44232742 | GTAA | - | frameshift deletion | NFKBIE | NM_004556 | c.759_762del | p.T253fs |
| U14 | CGP-336 | chr7 | 116340292 | 116340292 | G | A | nonsynonymous SNV | MET | NM_000245 | c.G1154A | p.C385Y |
| U14 | CGP-336 | chr7 | 151921140 | 151921140 | T | C | nonsynonymous SNV | KMT2C | NM_170606 | c.A3283G | p.R1095G |
| U14 | CGP-336 | chrX | 53432177 | 53432177 | T | C | synonymous SNV | SMC1A | NM_006306 | c.A2058G | p.K686K |
| U15 | CGP-337 | chr16 | 396543 | 396543 | C | T | synonymous SNV | AXIN1 | NM_003502 | c.G483A | p.K161K |
| U15 | CGP-337 | chr2 | 121746158 | 121746158 | C | T | nonsynonymous SNV | GLI2 | NM_005270 | c.C2668T | p.R890W |
| U15 | CGP-337 | chr2 | 198266611 | 198266611 | C | T | nonsynonymous SNV | SF3B1 | NM_012433 | c.G2225A | p.G742D |
| U15 | CGP-337 | chr20 | 49195089 | 49195089 | G | A | nonsynonymous SNV | PTPN1 | NM_001278618 | c.G406A | p.G136R |
| U15 | CGP-337 | chr7 | 91503540 | 91503540 | G | A | nonsynonymous SNV | MTERF1 | NM_001301134 | c.C508T | p.R170C |
| U16 | CGP-338 | chr10 | 89692771 | 89692771 | T | C | synonymous SNV | PTEN | NM_000314 | c.T255C | p.V85V |
| U16 | CGP-338 | chr17 | 63530161 | 63530161 | C | T | synonymous SNV | AXIN2 | NM_004655 | c.G2274A | p.A758A |
| U16 | CGP-338 | chr2 | 48010408 | 48010408 | C | T | synonymous SNV | MSH6 | NM_000179 | c.C36T | p.P12P |
| U16 | CGP-338 | chr22 | 41554435 | 41554435 | C | T | nonsynonymous SNV | EP300 | NM_001429 | c.C3521T | p.T1174I |
| U16 | CGP-338 | chr6 | 93965617 | 93965617 | C | A | nonsynonymous SNV | EPHA7 | NM_001288629 | c.G2296T | p.V766F |
| U20 | CGP-340 | chr10 | 75000751 | 75000751 | G | A | nonsynonymous SNV | FAM149B1 | NM_173348 | c.G1723A | p.V575I |
| U20 | CGP-340 | chr13 | 110435692 | 110435700 | GCTGGGCAG | - | nonframeshift deletion | IRS2 | NM_003749 | c.2701_2709del | p.901_903del |
| U20 | CGP-340 | chr2 | 47596696 | 47596696 | G | A | nonsynonymous SNV | EPCAM | NM_002354 | c.G52A | p.A18T |
| U20 | CGP-340 | chr2 | 233712223 | 233712223 | T | C | nonsynonymous SNV | GIGYF2 | NM_001103148 | c.T3608C | p.L1203P |
| U20 | CGP-340 | chrX | 76938464 | 76938464 | T | C | nonsynonymous SNV | ATRX | NM_138270 | c.A2170G | p.K724E |
| U22 | CGP-382 | chr1 | 162740212 | 162740212 | G | A | nonsynonymous SNV | DDR2 | NM_006182 | c.G1414A | p.D472N |
| U22 | CGP-382 | chr11 | 77991786 | 77991786 | C | G | nonsynonymous SNV | GAB2 | NM_012296 | c.G123C | p.E41D |
| U22 | CGP-382 | chr13 | 86370196 | 86370196 | C | T | nonsynonymous SNV | SLITRK6 | NM_032229 | c.G448A | p.V150M |
| U22 | CGP-382 | chr22 | 41574341 | 41574352 | ACCAGTTCCAGC | - | nonframeshift deletion | EP300 | NM_001429 | c.6626_6637del | p.2209_2213del |
| U22 | CGP-382 | chr3 | 138664794 | 138664794 | C | G | synonymous SNV | FOXL2 | NM_023067 | c.G771C | p.P257P |
| U22 | CGP-382 | chr5 | 56184129 | 56184129 | C | G | nonsynonymous SNV | MAP3K1 | NM_005921 | c.C4334G | p.A1445G |
| U22 | CGP-382 | chr7 | 98533284 | 98533284 | G | A | nonsynonymous SNV | TRRAP | NM_001244580 | c.G4097A | p.R1366Q |
| U22 | CGP-382 | chr8 | 55370875 | 55370875 | G | A | synonymous SNV | SOX17 | NM_022454 | c.G177A | p.A59A |
| U23 | CGP-383 | chr14 | 50626364 | 50626364 | C | A | nonsynonymous SNV | SOS2 | NM_006939 | c.G1637T | p.S546I |
| U23 | CGP-383 | chr15 | 88679704 | 88679704 | A | G | synonymous SNV | NTRK3 | NM_001007156 | c.T759C | p.T253T |
| U23 | CGP-383 | chr19 | 40900130 | 40900130 | G | A | nonsynonymous SNV | PRX | NM_181882 | c.C4129T | p.R1377C |
| U23 | CGP-383 | chr2 | 121726416 | 121726416 | T | C | nonsynonymous SNV | GLI2 | NM_005270 | c.T770C | p.V257A |
| U23 | CGP-383 | chr2 | 233712263 | 233712274 | ACAGCAGCCACA | - | nonframeshift deletion | GIGYF2 | NM_001103148 | c.3648_3659del | p.1216_1220del |
| U23 | CGP-383 | chr6 | 157505562 | 157505562 | A | G | synonymous SNV | ARID1B | NM_017519 | c.A3504G | p.P1168P |
| U24 | CGP-384 | chr12 | 7526144 | 7526144 | C | T | nonsynonymous SNV | CD163L1 | NM_001297650 | c.G3532A | p.G1178S |
| U24 | CGP-384 | chr16 | 79633470 | 79633470 | G | C | synonymous SNV | MAF | NM_001031804 | c.C330G | p.P110P |
| U24 | CGP-384 | chr19 | 2210774 | 2210774 | G | A | nonsynonymous SNV | DOT1L | NM_032482 | c.G1271A | p.R424Q |
| U24 | CGP-384 | chr2 | 58388756 | 58388756 | A | G | synonymous SNV | FANCL | NM_001114636 | c.T936C | p.C312C |
| U24 | CGP-384 | chr20 | 39766463 | 39766463 | C | T | nonsynonymous SNV | PLCG1 | NM_002660 | c.C182T | p.T61M |
| U24 | CGP-384 | chr3 | 168833462 | 168833462 | A | G | nonsynonymous SNV | MECOM | NM_001105078 | c.T1634C | p.L545P |
| U24 | CGP-384 | chr5 | 149459791 | 149459791 | G | A | nonsynonymous SNV | CSF1R | NM_001288705 | c.C416T | p.S139L |
| U24 | CGP-384 | chr8 | 31015039 | 31015039 | C | T | synonymous SNV | WRN | NM_000553 | c.C3975T | p.V1325V |
| U24 | CGP-384 | chr9 | 98211440 | 98211440 | G | A | nonsynonymous SNV | PTCH1 | NM_000264 | c.C3715T | p.R1239W |
| U25 | CGP-385 | chr11 | 101981600 | 101981600 | G | A | synonymous SNV | YAP1 | NM_001130145 | c.G21A | p.P7P |
| U25 | CGP-385 | chr14 | 50600913 | 50600913 | T | C | synonymous SNV | SOS2 | NM_006939 | c.A3003G | p.K1001K |
| U25 | CGP-385 | chr15 | 34649211 | 34649211 | C | T | nonsynonymous SNV | NUTM1 | NM_001284293 | c.C2972T | p.T991I |
| U25 | CGP-385 | chr17 | 7579364 | 7579364 | C | T | nonsynonymous SNV | TP53 | NM_001126118 | c.G206A | p.G69D |
| U25 | CGP-385 | chr19 | 40902144 | 40902144 | G | C | nonsynonymous SNV | PRX | NM_181882 | c.C2115G | p.H705Q |
| U25 | CGP-385 | chr2 | 213872308 | 213872308 | A | G | nonsynonymous SNV | IKZF2 | NM_016260 | c.T1357C | p.S453P |
| U25 | CGP-385 | chr22 | 41533706 | 41533706 | A | T | nonsynonymous SNV | EP300 | NM_001429 | c.A1672T | p.T558S |
| U25 | CGP-385 | chr3 | 168834211 | 168834211 | T | C | synonymous SNV | MECOM | NM_001105078 | c.A885G | p.T295T |
| U25 | CGP-385 | chr5 | 180050961 | 180050961 | T | A | nonsynonymous SNV | FLT4 | NM_002020 | c.A1522T | p.T508S |
| U25 | CGP-385 | chr5 | 36972109 | 36972109 | A | G | synonymous SNV | NIPBL | NM_015384 | c.A834G | p.V278V |
| U25 | CGP-385 | chr6 | 112020758 | 112020758 | C | T | synonymous SNV | FYN | NM_153047 | c.G813A | p.E271E |
| U25 | CGP-385 | chr6 | 160525726 | 160525726 | A | T | synonymous SNV | IGF2R | NM_000876 | c.A7086T | p.T2362T |
| U26 | CGP-386 | chr1 | 16262142 | 16262142 | G | A | nonsynonymous SNV | SPEN | NM_015001 | c.G9407A | p.R3136H |
| U26 | CGP-386 | chr12 | 111856181 | 111856181 | G | A | nonsynonymous SNV | SH2B3 | NM_005475 | c.G232A | p.E78K |
| U26 | CGP-386 | chr12 | 111856288 | 111856288 | T | C | synonymous SNV | SH2B3 | NM_005475 | c.T339C | p.P113P |
| U26 | CGP-386 | chr14 | 99640887 | 99640887 | G | A | synonymous SNV | BCL11B | NM_001282238 | c.C2070T | p.G690G |
| U26 | CGP-386 | chr16 | 89862408 | 89862408 | T | C | synonymous SNV | FANCA | NM_000135 | c.A912G | p.G304G |
| U26 | CGP-386 | chr4 | 1941385 | 1941385 | G | A | synonymous SNV | WHSC1 | NM_133334 | c.G1761A | p.T587T |
| U26 | CGP-386 | chr6 | 157099418 | 157099426 | CAGCAGCAA | - | nonframeshift deletion | ARID1B | NM_017519 | c.355_363del | p.119_121del |
| U27 | CGP-387 | chr1 | 158584049 | 158584049 | A | G | nonsynonymous SNV | SPTA1 | NM_003126 | c.T6836C | p.I2279T |
| U27 | CGP-387 | chr11 | 22646327 | 22646327 | G | T | nonsynonymous SNV | FANCF | NM_022725 | c.C1030A | p.L344I |
| U27 | CGP-387 | chr12 | 58144709 | 58144709 | G | A | synonymous SNV | CDK4 | NM_000075 | c.C519T | p.P173P |
| U27 | CGP-387 | chr15 | 91295135 | 91295135 | A | G | synonymous SNV | BLM | NM_000057 | c.A918G | p.E306E |
| U27 | CGP-387 | chr16 | 72821616 | 72821618 | CCA | - | nonframeshift deletion | ZFHX3 | NM_001164766 | c.7815_7817del | p.2605_2606del |
| U27 | CGP-387 | chr19 | 13051383 | 13051383 | A | G | nonsynonymous SNV | CALR | NM_004343 | c.A731G | p.D244G |
| U27 | CGP-387 | chr21 | 39764328 | 39764328 | C | T | nonsynonymous SNV | ERG | NM_001136155 | c.G508A | p.G170S |
| U27 | CGP-387 | chr5 | 112174037 | 112174037 | A | G | nonsynonymous SNV | APC | NM_001127511 | c.A2692G | p.T898A |
| U27 | CGP-387 | chr6 | 51920424 | 51920424 | C | T | synonymous SNV | PKHD1 | NM_138694 | c.G1797A | p.P599P |
| U27 | CGP-387 | chr9 | 21970997 | 21970997 | G | A | synonymous SNV | CDKN2A | NM_000077 | c.C361T | p.L121L |
| U28 | CGP-409 | chr3 | 183209717 | 183209718 | AG | - | frameshift deletion | KLHL6 | NM_130446 | c.1863_1864del | p.V621fs |
| U28 | CGP-409 | chr5 | 180050961 | 180050961 | T | A | nonsynonymous SNV | FLT4 | NM_002020 | c.A1522T | p.T508S |
| U29 | CGP-388 | chr13 | 86368957 | 86368957 | C | A | stopgain | SLITRK6 | NM_032229 | c.G1687T | p.E563X |
| U29 | CGP-388 | chr14 | 99641652 | 99641652 | G | A | synonymous SNV | BCL11B | NM_001282238 | c.C1305T | p.G435G |
| U29 | CGP-388 | chr16 | 72993012 | 72993012 | T | C | nonsynonymous SNV | ZFHX3 | NM_006885 | c.A1033G | p.K345E |
| U29 | CGP-388 | chr16 | 9857210 | 9857210 | A | G | synonymous SNV | GRIN2A | NM_001134407 | c.T4191C | p.N1397N |
| U29 | CGP-388 | chr2 | 61149071 | 61149071 | C | T | nonsynonymous SNV | REL | NM_001291746 | c.C1165T | p.R389C |
| U29 | CGP-388 | chr2 | 141259351 | 141259351 | C | T | nonsynonymous SNV | LRP1B | NM_018557 | c.G8755A | p.G2919S |
| U32 | CGP-412 | chr16 | 11349250 | 11349250 | G | A | nonsynonymous SNV | SOCS1 | NM_003745 | c.C86T | p.S29F |
| U32 | CGP-412 | chr7 | 106509011 | 106509011 | C | T | synonymous SNV | PIK3CG | NM_001282426 | c.C1005T | p.Y335Y |
| U32 | CGP-412 | chrX | 70462260 | 70462263 | GAGA | - | frameshift deletion | ZMYM3 | NM_001171162 | c.3523_3526del | p.S1175fs |
| U33 | CGP-389 | chr1 | 156845349 | 156845349 | G | T | nonsynonymous SNV | NTRK1 | NM_001012331 | c.G1374T | p.M458I |
| U33 | CGP-389 | chr11 | 102199652 | 102199652 | G | T | stopgain | BIRC3 | NM_001165 | c.G1057T | p.G353X |
| U33 | CGP-389 | chr11 | 102207681 | 102207681 | A | T | stopgain | BIRC3 | NM_001165 | c.A1663T | p.R555X |
| U33 | CGP-389 | chr12 | 49434037 | 49434037 | G | A | nonsynonymous SNV | KMT2D | NM_003482 | c.C7516T | p.L2506F |
| U33 | CGP-389 | chr13 | 28636192 | 28636192 | C | T | synonymous SNV | FLT3 | NM_004119 | c.G180A | p.P60P |
| U33 | CGP-389 | chr15 | 41988992 | 41988992 | C | T | nonsynonymous SNV | MGA | NM_001080541 | c.C1784T | p.T595I |
| U33 | CGP-389 | chr2 | 61719472 | 61719472 | C | T | nonsynonymous SNV | XPO1 | NM_003400 | c.G1711A | p.E571K |
| U33 | CGP-389 | chr20 | 39750651 | 39750651 | T | C | nonsynonymous SNV | TOP1 | NM_003286 | c.T2051C | p.V684A |
| U34 | CGP-390 | chr11 | 108213998 | 108213998 | C | T | nonsynonymous SNV | ATM | NM_000051 | c.C8318T | p.T2773I |
| U34 | CGP-390 | chr13 | 32912652 | 32912652 | T | A | stopgain | BRCA2 | NM_000059 | c.T4160A | p.L1387X |
| U34 | CGP-390 | chr17 | 47696601 | 47696601 | G | C | nonsynonymous SNV | SPOP | NM_001007228 | c.C347G | p.A116G |
| U34 | CGP-390 | chr2 | 198266713 | 198266713 | C | T | nonsynonymous SNV | SF3B1 | NM_012433 | c.G2219A | p.G740E |
| U34 | CGP-390 | chr5 | 145515185 | 145515185 | T | C | nonsynonymous SNV | LARS | NM_020117 | c.A2191G | p.I731V |
| U34 | CGP-390 | chr8 | 144941157 | 144941157 | C | T | nonsynonymous SNV | EPPK1 | NM_031308 | c.G6265A | p.A2089T |
| U34 | CGP-390 | chr9 | 139390649 | 139390650 | AG | - | frameshift deletion | NOTCH1 | NM_017617 | c.7541_7542del | p.P2514fs |
| U40 | CGP-415 | chr17 | 56432330 | 56432330 | C | T | nonsynonymous SNV | RNF43 | NM_017763 | c.G2326A | p.E776K |
| U40 | CGP-415 | chr9 | 139390649 | 139390650 | AG | - | frameshift deletion | NOTCH1 | NM_017617 | c.7541_7542del | p.P2514fs |
| U42 | CGP-416 | chr11 | 67353650 | 67353650 | C | A | nonsynonymous SNV | GSTP1 | NM_000852 | c.C412A | p.Q138K |
| U42 | CGP-416 | chr13 | 86369137 | 86369137 | A | G | synonymous SNV | SLITRK6 | NM_032229 | c.T1507C | p.L503L |
| U42 | CGP-416 | chr15 | 75668174 | 75668174 | C | T | synonymous SNV | SIN3A | NM_001145357 | c.G3423A | p.E1141E |
| U42 | CGP-416 | chr5 | 180057225 | 180057225 | C | T | synonymous SNV | FLT4 | NM_002020 | c.G513A | p.S171S |
| U42 | CGP-416 | chr8 | 30973951 | 30973951 | G | A | synonymous SNV | WRN | NM_000553 | c.G2355A | p.R785R |
| U42 | CGP-416 | chr9 | 35076009 | 35076009 | - | T | frameshift insertion | FANCG | NM_004629 | c.1092dupA | p.E365fs |
| U42 | CGP-416 | chrX | 70468610 | 70468610 | C | A | nonsynonymous SNV | ZMYM3 | NM_001171162 | c.G1663T | p.D555Y |
| U71 | CGP-465 | chr11 | 45924054 | 45924054 | G | T | nonsynonymous SNV | MAPK8IP1 | NM_005456 | c.G736T | p.A246S |
| U71 | CGP-465 | chr14 | 95570206 | 95570206 | T | C | nonsynonymous SNV | DICER1 | NM_001195573 | c.A3527G | p.N1176S |
| U71 | CGP-465 | chr14 | 94521405 | 94521405 | C | T | synonymous SNV | DDX24 | NM_020414 | c.G2115A | p.T705T |
| U71 | CGP-465 | chr16 | 67116204 | 67116204 | A | G | nonsynonymous SNV | CBFB | NM_001755 | c.A488G | p.E163G |
| U71 | CGP-465 | chr17 | 16068442 | 16068442 | A | G | nonsynonymous SNV | NCOR1 | NM_001190438 | c.T142C | p.S48P |
| U74 | CGP-468 | chr11 | 119156022 | 119156022 | C | T | nonsynonymous SNV | CBL | NM_005188 | c.C1687T | p.R563C |
| U74 | CGP-468 | chr13 | 110437358 | 110437358 | G | T | nonsynonymous SNV | IRS2 | NM_003749 | c.C1043A | p.A348D |
| U74 | CGP-468 | chr17 | 38512408 | 38512408 | C | T | nonsynonymous SNV | RARA | NM_001145302 | c.C1028T | p.P343L |
| U74 | CGP-468 | chr18 | 42532681 | 42532681 | G | A | nonsynonymous SNV | SETBP1 | NM_015559 | c.G3376A | p.G1126S |
| U74 | CGP-468 | chr2 | 198266611 | 198266611 | C | T | nonsynonymous SNV | SF3B1 | NM_012433 | c.G2225A | p.G742D |
| U74 | CGP-468 | chr3 | 59999857 | 59999857 | C | T | nonsynonymous SNV | FHIT | NM_001166243 | c.G125A | p.R42Q |
| U74 | CGP-468 | chr4 | 1803183 | 1803183 | G | T | nonsynonymous SNV | FGFR3 | NM_000142 | c.G535T | p.A179S |
| U74 | CGP-468 | chr9 | 110251327 | 110251327 | G | T | nonsynonymous SNV | KLF4 | NM_004235 | c.C10A | p.P4T |

**Table S3: Copy Number Aberrations (CNA) in Exposed and Unexposed Cases.**

| **Patient ID#** |  | **Chr** | **Start** | **End** | **log2ratio** | **#probes** |
| --- | --- | --- | --- | --- | --- | --- |
| U6 | CGP-460 | chr1 | 93709 | 39186475 | -0.3226 | 392 |
| U6 | CGP-460 | chr1 | 39186475 | 97522893 | -0.0185 | 501 |
| U6 | CGP-460 | chr1 | 97522893 | 98514340 | -2.0095 | 11 |
| U6 | CGP-460 | chr1 | 98514340 | 121286064 | -0.0646 | 212 |
| U6 | CGP-460 | chr1 | 142633228 | 249153513 | -0.0993 | 933 |
| U6 | CGP-460 | chr2 | 85990 | 25833941 | -0.0775 | 219 |
| U6 | CGP-460 | chr2 | 25833941 | 26130491 | -3.3071 | 2 |
| U6 | CGP-460 | chr2 | 26130491 | 39165672 | 0.0652 | 139 |
| U6 | CGP-460 | chr2 | 39165672 | 39613012 | -2.9881 | 3 |
| U6 | CGP-460 | chr2 | 39613012 | 90458314 | -0.0481 | 420 |
| U6 | CGP-460 | chr2 | 91668610 | 92252664 | -1.4598 | 5 |
| U6 | CGP-460 | chr2 | 95389401 | 140865672 | -0.1546 | 355 |
| U6 | CGP-460 | chr2 | 140865672 | 142061721 | -3.7838 | 8 |
| U6 | CGP-460 | chr2 | 142061721 | 243170925 | -0.1516 | 868 |
| U6 | CGP-460 | chr3 | 133004 | 70970982 | -0.0623 | 677 |
| U6 | CGP-460 | chr3 | 70970982 | 71274268 | -3.6695 | 2 |
| U6 | CGP-460 | chr3 | 71274268 | 90264757 | -0.135 | 160 |
| U6 | CGP-460 | chr3 | 93518046 | 119405608 | -0.1736 | 222 |
| U6 | CGP-460 | chr3 | 119405608 | 119853435 | -3.0847 | 3 |
| U6 | CGP-460 | chr3 | 119853435 | 168751842 | -0.08 | 439 |
| U6 | CGP-460 | chr3 | 168751842 | 169462806 | -2.474 | 5 |
| U6 | CGP-460 | chr3 | 169462806 | 189304320 | -0.1763 | 180 |
| U6 | CGP-460 | chr3 | 189304320 | 189754617 | -3.6189 | 3 |
| U6 | CGP-460 | chr3 | 189754617 | 197888970 | -0.2839 | 60 |
| U6 | CGP-460 | chr4 | 83904 | 49574529 | -0.1035 | 369 |
| U6 | CGP-460 | chr4 | 52678368 | 108908279 | -0.0667 | 517 |
| U6 | CGP-460 | chr4 | 108908279 | 109204938 | -3.4812 | 2 |
| U6 | CGP-460 | chr4 | 109204938 | 190976530 | -0.1243 | 611 |
| U6 | CGP-460 | chr5 | 88178 | 46176126 | -0.1531 | 394 |
| U6 | CGP-460 | chr5 | 49488207 | 158102723 | -0.0895 | 1027 |
| U6 | CGP-460 | chr5 | 158102723 | 158552330 | -3.3694 | 3 |
| U6 | CGP-460 | chr5 | 158552330 | 170817719 | -0.0296 | 92 |
| U6 | CGP-460 | chr5 | 170817719 | 170879068 | 0.5817 | 16 |
| U6 | CGP-460 | chr5 | 170879068 | 176566583 | 0.0349 | 57 |
| U6 | CGP-460 | chr5 | 176566583 | 176858618 | -2.749 | 2 |
| U6 | CGP-460 | chr5 | 176858618 | 179970249 | -0.0917 | 23 |
| U6 | CGP-460 | chr5 | 179970249 | 180063168 | 0.2634 | 31 |
| U6 | CGP-460 | chr5 | 180063168 | 180835789 | -0.2754 | 6 |
| U6 | CGP-460 | chr6 | 276952 | 25969849 | -0.0793 | 202 |
| U6 | CGP-460 | chr6 | 25969849 | 26420118 | -3.6735 | 3 |
| U6 | CGP-460 | chr6 | 26420118 | 28586082 | -0.3429 | 16 |
| U6 | CGP-460 | chr6 | 33418193 | 58710556 | -0.0978 | 307 |
| U6 | CGP-460 | chr6 | 61948494 | 152002032 | -0.3165 | 804 |
| U6 | CGP-460 | chr6 | 152002032 | 152446573 | -3.8266 | 3 |
| U6 | CGP-460 | chr6 | 152446573 | 170982158 | -0.4558 | 227 |
| U6 | CGP-460 | chr7 | 121242 | 18152652 | -0.0906 | 162 |
| U6 | CGP-460 | chr7 | 18152652 | 19053551 | -3.4861 | 6 |
| U6 | CGP-460 | chr7 | 19053551 | 27752599 | -0.1214 | 68 |
| U6 | CGP-460 | chr7 | 27752599 | 28354402 | -3.39 | 4 |
| U6 | CGP-460 | chr7 | 28354402 | 57974477 | -0.1414 | 271 |
| U6 | CGP-460 | chr7 | 61118627 | 151811490 | -0.1355 | 987 |
| U6 | CGP-460 | chr7 | 151811490 | 152417286 | -3.0049 | 4 |
| U6 | CGP-460 | chr7 | 152417286 | 159081217 | -0.178 | 47 |
| U6 | CGP-460 | chr8 | 85562 | 41712704 | -0.053 | 472 |
| U6 | CGP-460 | chr8 | 41712704 | 43599480 | -0.9708 | 13 |
| U6 | CGP-460 | chr8 | 46894812 | 146224689 | -0.081 | 842 |
| U6 | CGP-460 | chr9 | 85971 | 47238906 | -0.1597 | 487 |
| U6 | CGP-460 | chr9 | 65543126 | 141079659 | -0.1034 | 778 |
| U6 | CGP-460 | chr10 | 132497 | 7959302 | -0.0883 | 57 |
| U6 | CGP-460 | chr10 | 7959302 | 8258556 | -3.7759 | 2 |
| U6 | CGP-460 | chr10 | 8258556 | 38976463 | -0.058 | 218 |
| U6 | CGP-460 | chr10 | 42701108 | 63603781 | -0.1093 | 176 |
| U6 | CGP-460 | chr10 | 63603781 | 63904417 | -3.9709 | 2 |
| U6 | CGP-460 | chr10 | 63904417 | 114604780 | -0.1022 | 445 |
| U6 | CGP-460 | chr10 | 114604780 | 115055005 | -3.3731 | 3 |
| U6 | CGP-460 | chr10 | 115055005 | 135442105 | -0.0716 | 172 |
| U6 | CGP-460 | chr11 | 139098 | 50401447 | -0.1035 | 382 |
| U6 | CGP-460 | chr11 | 51404035 | 51530482 | -1.0593 | 2 |
| U6 | CGP-460 | chr11 | 55028532 | 134873895 | -0.0917 | 813 |
| U6 | CGP-460 | chr12 | 77870 | 34611974 | -0.1435 | 375 |
| U6 | CGP-460 | chr12 | 37936846 | 78157985 | -0.1088 | 394 |
| U6 | CGP-460 | chr12 | 78157985 | 78608829 | -3.6986 | 3 |
| U6 | CGP-460 | chr12 | 78608829 | 133756626 | -0.0196 | 435 |
| U6 | CGP-460 | chr13 | 19104207 | 115017146 | -0.1156 | 884 |
| U6 | CGP-460 | chr14 | 19110467 | 107211129 | -0.094 | 752 |
| U6 | CGP-460 | chr15 | 20098901 | 102435164 | -0.0252 | 776 |
| U6 | CGP-460 | chr16 | 133529 | 31105680 | 0.0228 | 370 |
| U6 | CGP-460 | chr16 | 31105680 | 35208244 | -0.3796 | 44 |
| U6 | CGP-460 | chr16 | 46443761 | 90225738 | -0.0362 | 414 |
| U6 | CGP-460 | chr17 | 74407 | 22188081 | -0.2477 | 192 |
| U6 | CGP-460 | chr17 | 25293732 | 81100055 | -0.136 | 702 |
| U6 | CGP-460 | chr18 | 91319 | 15321486 | 0.1468 | 111 |
| U6 | CGP-460 | chr18 | 18528870 | 42156109 | 0.2073 | 191 |
| U6 | CGP-460 | chr18 | 42156109 | 42754349 | -3.2334 | 4 |
| U6 | CGP-460 | chr18 | 42754349 | 52964522 | 0.2652 | 145 |
| U6 | CGP-460 | chr18 | 52964522 | 53403939 | -0.9868 | 12 |
| U6 | CGP-460 | chr18 | 53403939 | 60843274 | 0.2617 | 67 |
| U6 | CGP-460 | chr18 | 60843274 | 78009423 | -0.1 | 129 |
| U6 | CGP-460 | chr19 | 275349 | 24397443 | -0.0082 | 370 |
| U6 | CGP-460 | chr19 | 28283155 | 59042307 | -0.0723 | 290 |
| U6 | CGP-460 | chr20 | 133209 | 26237717 | -0.0307 | 190 |
| U6 | CGP-460 | chr20 | 29478404 | 40653188 | -0.0473 | 151 |
| U6 | CGP-460 | chr20 | 40653188 | 41551517 | -2.6682 | 6 |
| U6 | CGP-460 | chr20 | 41551517 | 62886880 | -0.1465 | 196 |
| U6 | CGP-460 | chr21 | 9464747 | 11106090 | -0.7472 | 10 |
| U6 | CGP-460 | chr21 | 14424106 | 48074396 | -0.1691 | 281 |
| U6 | CGP-460 | chr22 | 16131356 | 51186435 | 0.0143 | 485 |
| U6 | CGP-460 | chrX | 2709879 | 53147034 | -0.1035 | 467 |
| U6 | CGP-460 | chrX | 53147034 | 53577363 | -3.0934 | 3 |
| U6 | CGP-460 | chrX | 53577363 | 57873050 | 0.0216 | 32 |
| U6 | CGP-460 | chrX | 61931753 | 76707518 | -0.1909 | 141 |
| U6 | CGP-460 | chrX | 76707518 | 77136372 | -3.1443 | 3 |
| U6 | CGP-460 | chrX | 77136372 | 129040368 | -0.0783 | 432 |
| U6 | CGP-460 | chrX | 129040368 | 129302518 | -3.3223 | 2 |
| U6 | CGP-460 | chrX | 129302518 | 154882523 | -0.0751 | 188 |
| U6 | CGP-460 | chrY | 2654633 | 9535491 | -0.1687 | 43 |
| U6 | CGP-460 | chrY | 13199529 | 19586679 | -0.1107 | 37 |
| U6 | CGP-460 | chrY | 21033995 | 24521747 | -0.0274 | 18 |
| U6 | CGP-460 | chrY | 27495883 | 28590746 | -0.6216 | 6 |
| U42 | CGP-416 | chr1 | 93709 | 121286064 | -0.1224 | 1109 |
| U42 | CGP-416 | chr1 | 142633228 | 249153513 | -0.0969 | 927 |
| U42 | CGP-416 | chr2 | 85990 | 90458314 | -0.0962 | 776 |
| U42 | CGP-416 | chr2 | 91668610 | 92252664 | -0.0864 | 5 |
| U42 | CGP-416 | chr2 | 95389401 | 243170925 | -0.123 | 1219 |
| U42 | CGP-416 | chr3 | 133004 | 90264757 | -0.0861 | 838 |
| U42 | CGP-416 | chr3 | 93518046 | 187651719 | -0.0937 | 832 |
| U42 | CGP-416 | chr3 | 187651719 | 188102125 | -2.3433 | 3 |
| U42 | CGP-416 | chr3 | 188102125 | 197888970 | -0.1736 | 71 |
| U42 | CGP-416 | chr4 | 83904 | 49574529 | -0.1122 | 369 |
| U42 | CGP-416 | chr4 | 52678368 | 190976530 | -0.0686 | 1124 |
| U42 | CGP-416 | chr5 | 88178 | 46176126 | -0.0835 | 394 |
| U42 | CGP-416 | chr5 | 49488207 | 180697846 | -0.0663 | 1246 |
| U42 | CGP-416 | chr6 | 276952 | 28437090 | -0.0753 | 220 |
| U42 | CGP-416 | chr6 | 33418193 | 58710556 | -0.0783 | 302 |
| U42 | CGP-416 | chr6 | 61948494 | 170982158 | -0.085 | 1025 |
| U42 | CGP-416 | chr7 | 121242 | 57974477 | -0.0715 | 509 |
| U42 | CGP-416 | chr7 | 61118627 | 159081217 | -0.0493 | 1028 |
| U42 | CGP-416 | chr8 | 85562 | 43599480 | -0.309 | 481 |
| U42 | CGP-416 | chr8 | 46894812 | 146224689 | -0.0674 | 838 |
| U42 | CGP-416 | chr9 | 85971 | 47238906 | -0.0518 | 473 |
| U42 | CGP-416 | chr9 | 65543126 | 141079659 | -0.0729 | 770 |
| U42 | CGP-416 | chr10 | 132497 | 38976463 | -0.059 | 276 |
| U42 | CGP-416 | chr10 | 42701108 | 135442105 | -0.1271 | 787 |
| U42 | CGP-416 | chr11 | 139098 | 50401447 | -0.0819 | 382 |
| U42 | CGP-416 | chr11 | 51404035 | 51530482 | -0.2636 | 2 |
| U42 | CGP-416 | chr11 | 55028532 | 134873895 | -0.138 | 803 |
| U42 | CGP-416 | chr12 | 77870 | 34611974 | -0.0662 | 374 |
| U42 | CGP-416 | chr12 | 37936846 | 133756626 | -0.0522 | 828 |
| U42 | CGP-416 | chr13 | 19104207 | 115017146 | -0.096 | 878 |
| U42 | CGP-416 | chr14 | 19110467 | 107211129 | -0.0814 | 750 |
| U42 | CGP-416 | chr15 | 20098901 | 102435164 | -0.0938 | 763 |
| U42 | CGP-416 | chr16 | 133529 | 35208244 | -0.0725 | 409 |
| U42 | CGP-416 | chr16 | 46443761 | 90225738 | -0.1573 | 407 |
| U42 | CGP-416 | chr17 | 74407 | 22188081 | -0.4294 | 186 |
| U42 | CGP-416 | chr17 | 25293732 | 81100055 | 0.1047 | 695 |
| U42 | CGP-416 | chr18 | 91319 | 15321486 | -0.0552 | 110 |
| U42 | CGP-416 | chr18 | 18528870 | 78009423 | -0.1505 | 545 |
| U42 | CGP-416 | chr19 | 275349 | 24397443 | 0.0383 | 365 |
| U42 | CGP-416 | chr19 | 28283155 | 59042307 | -0.0783 | 289 |
| U42 | CGP-416 | chr20 | 133209 | 26237717 | -0.157 | 189 |
| U42 | CGP-416 | chr20 | 29478404 | 62886880 | -0.2351 | 350 |
| U42 | CGP-416 | chr21 | 9464747 | 11106090 | -0.449 | 10 |
| U42 | CGP-416 | chr21 | 14424106 | 48074396 | -0.1077 | 278 |
| U42 | CGP-416 | chr22 | 16131356 | 51186435 | -0.0787 | 482 |
| U42 | CGP-416 | chrX | 2709879 | 57873050 | -0.0632 | 496 |
| U42 | CGP-416 | chrX | 61931753 | 154882523 | -0.1618 | 752 |
| U42 | CGP-416 | chrY | 2654633 | 9535491 | -0.359 | 41 |
| U42 | CGP-416 | chrY | 13199529 | 19586679 | -0.1006 | 35 |
| U42 | CGP-416 | chrY | 21033995 | 24521747 | 0.1596 | 18 |
| U42 | CGP-416 | chrY | 28463067 | 28590746 | 0.267 | 5 |
| U32 | CGP-412 | chr1 | 93709 | 45251691 | -0.2053 | 444 |
| U32 | CGP-412 | chr1 | 45251691 | 45297547 | 0.3339 | 20 |
| U32 | CGP-412 | chr1 | 45297547 | 120534651 | -0.0613 | 641 |
| U32 | CGP-412 | chr1 | 120534651 | 121286064 | 0.3109 | 11 |
| U32 | CGP-412 | chr1 | 142633228 | 156834590 | -0.166 | 115 |
| U32 | CGP-412 | chr1 | 156834590 | 156841748 | 0.5815 | 8 |
| U32 | CGP-412 | chr1 | 156841748 | 249153513 | -0.0395 | 810 |
| U32 | CGP-412 | chr2 | 85990 | 1558215 | -0.2805 | 11 |
| U32 | CGP-412 | chr2 | 1558215 | 24311539 | 0.0228 | 169 |
| U32 | CGP-412 | chr2 | 24311539 | 29377881 | -0.2381 | 65 |
| U32 | CGP-412 | chr2 | 29377881 | 30181462 | 0.232 | 49 |
| U32 | CGP-412 | chr2 | 30181462 | 90458314 | -0.0641 | 489 |
| U32 | CGP-412 | chr2 | 91668610 | 92252664 | 0.2696 | 5 |
| U32 | CGP-412 | chr2 | 95389401 | 212210903 | 0.0243 | 881 |
| U32 | CGP-412 | chr2 | 212210903 | 212994598 | 0.5056 | 38 |
| U32 | CGP-412 | chr2 | 212994598 | 243170925 | -0.0134 | 312 |
| U32 | CGP-412 | chr3 | 133004 | 52397116 | -0.0371 | 472 |
| U32 | CGP-412 | chr3 | 52397116 | 52568085 | -0.4914 | 21 |
| U32 | CGP-412 | chr3 | 52568085 | 52626775 | -0.1872 | 13 |
| U32 | CGP-412 | chr3 | 52626775 | 52684101 | -0.454 | 13 |
| U32 | CGP-412 | chr3 | 52684101 | 70819338 | 0.0624 | 157 |
| U32 | CGP-412 | chr3 | 70819338 | 71425912 | -0.5896 | 4 |
| U32 | CGP-412 | chr3 | 71425912 | 89171511 | 0.0657 | 130 |
| U32 | CGP-412 | chr3 | 89171511 | 90264757 | 0.487 | 29 |
| U32 | CGP-412 | chr3 | 93518046 | 96500173 | 0.0763 | 26 |
| U32 | CGP-412 | chr3 | 96500173 | 97480000 | 0.3794 | 39 |
| U32 | CGP-412 | chr3 | 97480000 | 197888970 | 0.0238 | 847 |
| U32 | CGP-412 | chr4 | 83904 | 3152602 | -0.3029 | 35 |
| U32 | CGP-412 | chr4 | 3152602 | 48038301 | -0.0104 | 324 |
| U32 | CGP-412 | chr4 | 48038301 | 49574529 | -0.4158 | 10 |
| U32 | CGP-412 | chr4 | 52678368 | 55084656 | -0.0848 | 22 |
| U32 | CGP-412 | chr4 | 55084656 | 56138739 | 0.3494 | 85 |
| U32 | CGP-412 | chr4 | 56138739 | 66147800 | 0.0252 | 69 |
| U32 | CGP-412 | chr4 | 66147800 | 66575943 | 0.5418 | 27 |
| U32 | CGP-412 | chr4 | 66575943 | 190976530 | 0.0436 | 927 |
| U32 | CGP-412 | chr5 | 88178 | 46176126 | 0.0396 | 394 |
| U32 | CGP-412 | chr5 | 49488207 | 94220686 | 0.0349 | 417 |
| U32 | CGP-412 | chr5 | 94220686 | 94658840 | 0.5575 | 23 |
| U32 | CGP-412 | chr5 | 94658840 | 112173995 | 0.0375 | 147 |
| U32 | CGP-412 | chr5 | 112173995 | 112340382 | -0.2834 | 23 |
| U32 | CGP-412 | chr5 | 112340382 | 125842174 | 0.0357 | 97 |
| U32 | CGP-412 | chr5 | 125842174 | 126049932 | 0.3986 | 20 |
| U32 | CGP-412 | chr5 | 126049932 | 180835789 | 0.0174 | 530 |
| U32 | CGP-412 | chr6 | 276952 | 28586082 | -0.0639 | 221 |
| U32 | CGP-412 | chr6 | 33418193 | 51444238 | -0.086 | 168 |
| U32 | CGP-412 | chr6 | 51444238 | 51947629 | 0.4017 | 88 |
| U32 | CGP-412 | chr6 | 51947629 | 58710556 | 0.0233 | 51 |
| U32 | CGP-412 | chr6 | 61948494 | 93479248 | 0.0382 | 229 |
| U32 | CGP-412 | chr6 | 93479248 | 94081102 | 0.5119 | 19 |
| U32 | CGP-412 | chr6 | 94081102 | 106538253 | 0.1058 | 101 |
| U32 | CGP-412 | chr6 | 106538253 | 110574300 | -0.2958 | 27 |
| U32 | CGP-412 | chr6 | 110574300 | 116403145 | 0.0716 | 42 |
| U32 | CGP-412 | chr6 | 116403145 | 117042128 | -0.4684 | 5 |
| U32 | CGP-412 | chr6 | 117042128 | 117641679 | 0.0064 | 6 |
| U32 | CGP-412 | chr6 | 117641679 | 117653999 | 0.3106 | 34 |
| U32 | CGP-412 | chr6 | 117653999 | 117658394 | 0.8255 | 3 |
| U32 | CGP-412 | chr6 | 117658394 | 117698795 | 0.4133 | 15 |
| U32 | CGP-412 | chr6 | 117698795 | 128252135 | 0.0561 | 74 |
| U32 | CGP-412 | chr6 | 128252135 | 129607116 | 0.3353 | 44 |
| U32 | CGP-412 | chr6 | 129607116 | 170982158 | -0.0447 | 435 |
| U32 | CGP-412 | chr7 | 121242 | 27752599 | -0.0364 | 236 |
| U32 | CGP-412 | chr7 | 27752599 | 28354402 | -0.9005 | 4 |
| U32 | CGP-412 | chr7 | 28354402 | 57974477 | 0.0788 | 271 |
| U32 | CGP-412 | chr7 | 61118627 | 98547574 | 0.1105 | 344 |
| U32 | CGP-412 | chr7 | 98547574 | 99266688 | -0.3297 | 45 |
| U32 | CGP-412 | chr7 | 99266688 | 159081217 | 0.0206 | 649 |
| U32 | CGP-412 | chr8 | 85562 | 43599480 | 0.0629 | 485 |
| U32 | CGP-412 | chr8 | 46894812 | 92932731 | 0.038 | 416 |
| U32 | CGP-412 | chr8 | 92932731 | 93063460 | 0.7012 | 13 |
| U32 | CGP-412 | chr8 | 93063460 | 146224689 | 0.0212 | 413 |
| U32 | CGP-412 | chr9 | 85971 | 8277255 | 0.0143 | 166 |
| U32 | CGP-412 | chr9 | 8277255 | 8767714 | 0.4623 | 48 |
| U32 | CGP-412 | chr9 | 8767714 | 47238906 | 0.0659 | 272 |
| U32 | CGP-412 | chr9 | 65543126 | 66887179 | -0.3503 | 7 |
| U32 | CGP-412 | chr9 | 66887179 | 73232388 | 0.0638 | 68 |
| U32 | CGP-412 | chr9 | 73232388 | 73465513 | 0.5092 | 22 |
| U32 | CGP-412 | chr9 | 73465513 | 117784900 | 0.013 | 387 |
| U32 | CGP-412 | chr9 | 117784900 | 117894246 | 0.4058 | 32 |
| U32 | CGP-412 | chr9 | 117894246 | 123315284 | 0.0326 | 39 |
| U32 | CGP-412 | chr9 | 123315284 | 140421343 | -0.3204 | 218 |
| U32 | CGP-412 | chr9 | 140421343 | 141079659 | -0.1247 | 5 |
| U32 | CGP-412 | chr10 | 132497 | 8258556 | -0.1811 | 59 |
| U32 | CGP-412 | chr10 | 8258556 | 38976463 | 0.0822 | 218 |
| U32 | CGP-412 | chr10 | 42701108 | 96354112 | -0.0208 | 454 |
| U32 | CGP-412 | chr10 | 96354112 | 96762405 | 0.7963 | 4 |
| U32 | CGP-412 | chr10 | 96762405 | 104363197 | -0.0682 | 70 |
| U32 | CGP-412 | chr10 | 104363197 | 104388417 | -0.9778 | 4 |
| U32 | CGP-412 | chr10 | 104388417 | 111605184 | -0.006 | 54 |
| U32 | CGP-412 | chr10 | 111605184 | 112720814 | -0.4808 | 10 |
| U32 | CGP-412 | chr10 | 112720814 | 135442105 | 0.0181 | 202 |
| U32 | CGP-412 | chr11 | 139098 | 31958825 | -0.028 | 235 |
| U32 | CGP-412 | chr11 | 31958825 | 32593301 | 0.5141 | 17 |
| U32 | CGP-412 | chr11 | 32593301 | 50401447 | -0.06 | 130 |
| U32 | CGP-412 | chr11 | 51404035 | 51530482 | -0.1588 | 2 |
| U32 | CGP-412 | chr11 | 55028532 | 58204472 | 0.1318 | 26 |
| U32 | CGP-412 | chr11 | 58204472 | 77613119 | -0.2317 | 160 |
| U32 | CGP-412 | chr11 | 77613119 | 106630252 | 0.0484 | 257 |
| U32 | CGP-412 | chr11 | 106630252 | 106928972 | 0.4557 | 13 |
| U32 | CGP-412 | chr11 | 106928972 | 116558874 | -0.0115 | 130 |
| U32 | CGP-412 | chr11 | 116558874 | 119310482 | -0.3276 | 101 |
| U32 | CGP-412 | chr11 | 119310482 | 134873895 | -0.0003 | 126 |
| U32 | CGP-412 | chr12 | 77870 | 7469243 | 0.4065 | 60 |
| U32 | CGP-412 | chr12 | 7469243 | 9039717 | 0.7853 | 36 |
| U32 | CGP-412 | chr12 | 9039717 | 12022434 | 0.193 | 77 |
| U32 | CGP-412 | chr12 | 12022434 | 34611974 | 0.5135 | 202 |
| U32 | CGP-412 | chr12 | 37936846 | 133756626 | 0.4708 | 832 |
| U32 | CGP-412 | chr13 | 19104207 | 40539152 | 0.0564 | 294 |
| U32 | CGP-412 | chr13 | 40539152 | 41703531 | -0.4812 | 8 |
| U32 | CGP-412 | chr13 | 41703531 | 115017146 | 0.0015 | 582 |
| U32 | CGP-412 | chr14 | 19110467 | 101708244 | -0.0271 | 682 |
| U32 | CGP-412 | chr14 | 101708244 | 107211129 | -0.359 | 70 |
| U32 | CGP-412 | chr15 | 20098901 | 66778508 | -0.0561 | 436 |
| U32 | CGP-412 | chr15 | 66778508 | 66819090 | -0.8654 | 4 |
| U32 | CGP-412 | chr15 | 66819090 | 82691076 | -0.1903 | 131 |
| U32 | CGP-412 | chr15 | 82691076 | 83200197 | 1.2195 | 3 |
| U32 | CGP-412 | chr15 | 83200197 | 88461601 | -0.0145 | 40 |
| U32 | CGP-412 | chr15 | 88461601 | 88599530 | 0.6867 | 12 |
| U32 | CGP-412 | chr15 | 88599530 | 102435164 | -0.0995 | 149 |
| U32 | CGP-412 | chr16 | 133529 | 2617635 | -0.4202 | 58 |
| U32 | CGP-412 | chr16 | 2617635 | 2647444 | -1.2155 | 4 |
| U32 | CGP-412 | chr16 | 2647444 | 31195922 | -0.0985 | 310 |
| U32 | CGP-412 | chr16 | 31195922 | 31363207 | -0.9318 | 15 |
| U32 | CGP-412 | chr16 | 31363207 | 35208244 | 0.2961 | 27 |
| U32 | CGP-412 | chr16 | 46443761 | 50498155 | -0.0886 | 37 |
| U32 | CGP-412 | chr16 | 50498155 | 51067891 | -0.3344 | 24 |
| U32 | CGP-412 | chr16 | 51067891 | 90225738 | -0.0564 | 353 |
| U32 | CGP-412 | chr17 | 74407 | 22188081 | -0.1391 | 192 |
| U32 | CGP-412 | chr17 | 25293732 | 37866239 | -0.1558 | 180 |
| U32 | CGP-412 | chr17 | 37866239 | 37883831 | 0.1695 | 21 |
| U32 | CGP-412 | chr17 | 37883831 | 43411942 | -0.2197 | 109 |
| U32 | CGP-412 | chr17 | 43411942 | 43797065 | 1.206 | 2 |
| U32 | CGP-412 | chr17 | 43797065 | 81100055 | -0.2045 | 390 |
| U32 | CGP-412 | chr18 | 91319 | 15321486 | 0.0289 | 111 |
| U32 | CGP-412 | chr18 | 18528870 | 50394056 | 0.0322 | 276 |
| U32 | CGP-412 | chr18 | 50394056 | 51093015 | 0.4912 | 40 |
| U32 | CGP-412 | chr18 | 51093015 | 59117729 | -0.012 | 78 |
| U32 | CGP-412 | chr18 | 59117729 | 59262959 | 0.662 | 13 |
| U32 | CGP-412 | chr18 | 59262959 | 76479130 | 0.0444 | 128 |
| U32 | CGP-412 | chr18 | 76479130 | 78009423 | -0.2335 | 13 |
| U32 | CGP-412 | chr19 | 275349 | 15135892 | -0.1731 | 244 |
| U32 | CGP-412 | chr19 | 15135892 | 15305791 | 0.3639 | 36 |
| U32 | CGP-412 | chr19 | 15305791 | 24397443 | -0.0898 | 90 |
| U32 | CGP-412 | chr19 | 28283155 | 36916954 | -0.0379 | 77 |
| U32 | CGP-412 | chr19 | 36916954 | 38269859 | -0.6744 | 10 |
| U32 | CGP-412 | chr19 | 38269859 | 43052541 | -0.1804 | 56 |
| U32 | CGP-412 | chr19 | 43052541 | 43966451 | 0.3596 | 7 |
| U32 | CGP-412 | chr19 | 43966451 | 44981500 | -0.5594 | 7 |
| U32 | CGP-412 | chr19 | 44981500 | 46189319 | 0.1167 | 33 |
| U32 | CGP-412 | chr19 | 46189319 | 59042307 | -0.2231 | 100 |
| U32 | CGP-412 | chr20 | 133209 | 26237717 | -0.0441 | 190 |
| U32 | CGP-412 | chr20 | 29478404 | 62886880 | -0.2343 | 353 |
| U32 | CGP-412 | chr21 | 9464747 | 11106090 | 0.2311 | 10 |
| U32 | CGP-412 | chr21 | 14424106 | 42321798 | 0.0275 | 241 |
| U32 | CGP-412 | chr21 | 42321798 | 48074396 | -0.2668 | 40 |
| U32 | CGP-412 | chr22 | 16131356 | 41452476 | 0.2383 | 349 |
| U32 | CGP-412 | chr22 | 41452476 | 41517848 | 0.859 | 7 |
| U32 | CGP-412 | chr22 | 41517848 | 42368790 | 0.194 | 57 |
| U32 | CGP-412 | chr22 | 42368790 | 42632192 | -0.8987 | 9 |
| U32 | CGP-412 | chr22 | 42632192 | 51186435 | 0.3691 | 63 |
| U32 | CGP-412 | chrX | 2709879 | 53147034 | 0.0137 | 467 |
| U32 | CGP-412 | chrX | 53147034 | 53720806 | -0.6817 | 4 |
| U32 | CGP-412 | chrX | 53720806 | 57873050 | -0.0706 | 31 |
| U32 | CGP-412 | chrX | 61931753 | 65617641 | -0.2076 | 27 |
| U32 | CGP-412 | chrX | 65617641 | 70208528 | 0.2039 | 50 |
| U32 | CGP-412 | chrX | 70208528 | 71436013 | -0.3749 | 23 |
| U32 | CGP-412 | chrX | 71436013 | 76707518 | 0.1306 | 41 |
| U32 | CGP-412 | chrX | 76707518 | 78710417 | -0.3325 | 15 |
| U32 | CGP-412 | chrX | 78710417 | 109646102 | 0.1434 | 232 |
| U32 | CGP-412 | chrX | 109646102 | 110497425 | 0.4762 | 23 |
| U32 | CGP-412 | chrX | 110497425 | 154882523 | 0.0882 | 355 |
| U32 | CGP-412 | chrY | 2654633 | 9535491 | -0.192 | 43 |
| U32 | CGP-412 | chrY | 13199529 | 19586679 | -0.0234 | 37 |
| U32 | CGP-412 | chrY | 21033995 | 24521747 | 0.0655 | 18 |
| U32 | CGP-412 | chrY | 27495883 | 28590746 | 0.0305 | 6 |
| U34 | CGP-390 | chr1 | 93709 | 3564522 | -0.4562 | 40 |
| U34 | CGP-390 | chr1 | 3564522 | 11187448 | -0.093 | 67 |
| U34 | CGP-390 | chr1 | 11187448 | 11191943 | -0.6877 | 6 |
| U34 | CGP-390 | chr1 | 11191943 | 12908331 | -0.0325 | 51 |
| U34 | CGP-390 | chr1 | 12908331 | 13601019 | 0.8169 | 4 |
| U34 | CGP-390 | chr1 | 13601019 | 121286064 | -0.0975 | 948 |
| U34 | CGP-390 | chr1 | 142633228 | 249153513 | -0.0675 | 932 |
| U34 | CGP-390 | chr2 | 85990 | 29377881 | -0.0422 | 244 |
| U34 | CGP-390 | chr2 | 29377881 | 29525184 | 0.2878 | 32 |
| U34 | CGP-390 | chr2 | 29525184 | 90458314 | -0.0556 | 506 |
| U34 | CGP-390 | chr2 | 91668610 | 92252664 | 0.395 | 5 |
| U34 | CGP-390 | chr2 | 95389401 | 243170925 | 0.0163 | 1230 |
| U34 | CGP-390 | chr3 | 133004 | 52436507 | -0.0245 | 473 |
| U34 | CGP-390 | chr3 | 52436507 | 52443671 | -0.6998 | 15 |
| U34 | CGP-390 | chr3 | 52443671 | 53003858 | -0.2333 | 39 |
| U34 | CGP-390 | chr3 | 53003858 | 89238393 | 0.0461 | 285 |
| U34 | CGP-390 | chr3 | 89238393 | 90264757 | 0.3251 | 27 |
| U34 | CGP-390 | chr3 | 93518046 | 197888970 | 0.0371 | 912 |
| U34 | CGP-390 | chr4 | 83904 | 3152602 | -0.3571 | 35 |
| U34 | CGP-390 | chr4 | 3152602 | 48038301 | 0.0232 | 324 |
| U34 | CGP-390 | chr4 | 48038301 | 49574529 | -0.2478 | 10 |
| U34 | CGP-390 | chr4 | 52678368 | 190976530 | 0.0488 | 1130 |
| U34 | CGP-390 | chr5 | 88178 | 46176126 | 0.0348 | 394 |
| U34 | CGP-390 | chr5 | 49488207 | 94212817 | -0.0191 | 416 |
| U34 | CGP-390 | chr5 | 94212817 | 94658840 | 0.4654 | 24 |
| U34 | CGP-390 | chr5 | 94658840 | 112173995 | 0.0258 | 147 |
| U34 | CGP-390 | chr5 | 112173995 | 112178793 | -0.2869 | 18 |
| U34 | CGP-390 | chr5 | 112178793 | 125842174 | 0.0388 | 102 |
| U34 | CGP-390 | chr5 | 125842174 | 126049932 | 0.4794 | 20 |
| U34 | CGP-390 | chr5 | 126049932 | 180835789 | 0.0351 | 530 |
| U34 | CGP-390 | chr6 | 276952 | 24611171 | -0.0414 | 192 |
| U34 | CGP-390 | chr6 | 24611171 | 28586082 | -0.3804 | 29 |
| U34 | CGP-390 | chr6 | 33418193 | 44231574 | -0.2168 | 112 |
| U34 | CGP-390 | chr6 | 44231574 | 58710556 | 0.1503 | 195 |
| U34 | CGP-390 | chr6 | 61948494 | 154969833 | 0.0441 | 825 |
| U34 | CGP-390 | chr6 | 154969833 | 160517056 | -0.2783 | 127 |
| U34 | CGP-390 | chr6 | 160517056 | 170982158 | 0.1121 | 82 |
| U34 | CGP-390 | chr7 | 121242 | 57974477 | 0.0315 | 511 |
| U34 | CGP-390 | chr7 | 61118627 | 62462928 | -0.3872 | 4 |
| U34 | CGP-390 | chr7 | 62462928 | 76175022 | 0.3268 | 97 |
| U34 | CGP-390 | chr7 | 76175022 | 86357539 | -0.0222 | 75 |
| U34 | CGP-390 | chr7 | 86357539 | 86534662 | 0.411 | 13 |
| U34 | CGP-390 | chr7 | 86534662 | 95453691 | 0.0026 | 82 |
| U34 | CGP-390 | chr7 | 95453691 | 98443585 | 0.2845 | 38 |
| U34 | CGP-390 | chr7 | 98443585 | 99266688 | -0.223 | 80 |
| U34 | CGP-390 | chr7 | 99266688 | 159081217 | 0.0466 | 649 |
| U34 | CGP-390 | chr8 | 85562 | 43599480 | 0.0652 | 485 |
| U34 | CGP-390 | chr8 | 46894812 | 145494079 | 0.053 | 836 |
| U34 | CGP-390 | chr8 | 145494079 | 146224689 | -0.7201 | 6 |
| U34 | CGP-390 | chr9 | 85971 | 47238906 | 0.0489 | 487 |
| U34 | CGP-390 | chr9 | 65543126 | 123163255 | 0.0668 | 553 |
| U34 | CGP-390 | chr9 | 123163255 | 141079659 | -0.2957 | 224 |
| U34 | CGP-390 | chr10 | 132497 | 38976463 | 0.0606 | 277 |
| U34 | CGP-390 | chr10 | 42701108 | 102603445 | 0.0128 | 504 |
| U34 | CGP-390 | chr10 | 102603445 | 104429844 | -0.3491 | 27 |
| U34 | CGP-390 | chr10 | 104429844 | 135442105 | 0.0505 | 265 |
| U34 | CGP-390 | chr11 | 139098 | 50401447 | 0.0158 | 382 |
| U34 | CGP-390 | chr11 | 51404035 | 51530482 | -0.1844 | 2 |
| U34 | CGP-390 | chr11 | 55028532 | 58055825 | 0.1192 | 25 |
| U34 | CGP-390 | chr11 | 58055825 | 64384795 | -0.2127 | 46 |
| U34 | CGP-390 | chr11 | 64384795 | 66092221 | -0.4819 | 25 |
| U34 | CGP-390 | chr11 | 66092221 | 82540807 | -0.1398 | 140 |
| U34 | CGP-390 | chr11 | 82540807 | 118161915 | -0.7754 | 362 |
| U34 | CGP-390 | chr11 | 118161915 | 118428430 | -1.1367 | 64 |
| U34 | CGP-390 | chr11 | 118428430 | 123459611 | -0.8252 | 56 |
| U34 | CGP-390 | chr11 | 123459611 | 134873895 | 0.0094 | 95 |
| U34 | CGP-390 | chr12 | 77870 | 7469243 | -0.0932 | 60 |
| U34 | CGP-390 | chr12 | 7469243 | 9304394 | 0.2194 | 38 |
| U34 | CGP-390 | chr12 | 9304394 | 12022434 | -0.3045 | 75 |
| U34 | CGP-390 | chr12 | 12022434 | 12570224 | 0.2641 | 35 |
| U34 | CGP-390 | chr12 | 12570224 | 34611974 | -0.0195 | 167 |
| U34 | CGP-390 | chr12 | 37936846 | 133756626 | -0.02 | 832 |
| U34 | CGP-390 | chr13 | 19104207 | 48890295 | 0.0408 | 354 |
| U34 | CGP-390 | chr13 | 48890295 | 52657843 | -0.9471 | 55 |
| U34 | CGP-390 | chr13 | 52657843 | 115017146 | 0.0295 | 475 |
| U34 | CGP-390 | chr14 | 19110467 | 101406666 | -0.0094 | 680 |
| U34 | CGP-390 | chr14 | 101406666 | 106196483 | -0.3656 | 65 |
| U34 | CGP-390 | chr14 | 106196483 | 107211129 | -1.5935 | 7 |
| U34 | CGP-390 | chr15 | 20098901 | 22740799 | -0.5404 | 23 |
| U34 | CGP-390 | chr15 | 22740799 | 88421911 | -0.0718 | 588 |
| U34 | CGP-390 | chr15 | 88421911 | 88599530 | 0.4221 | 14 |
| U34 | CGP-390 | chr15 | 88599530 | 102435164 | -0.0899 | 149 |
| U34 | CGP-390 | chr16 | 133529 | 3908157 | -0.4588 | 114 |
| U34 | CGP-390 | chr16 | 3908157 | 31195922 | -0.0657 | 258 |
| U34 | CGP-390 | chr16 | 31195922 | 31363207 | -0.825 | 15 |
| U34 | CGP-390 | chr16 | 31363207 | 35208244 | 0.1168 | 27 |
| U34 | CGP-390 | chr16 | 46443761 | 77829749 | -0.0293 | 307 |
| U34 | CGP-390 | chr16 | 77829749 | 78311751 | 0.3929 | 14 |
| U34 | CGP-390 | chr16 | 78311751 | 85466799 | -0.0044 | 51 |
| U34 | CGP-390 | chr16 | 85466799 | 90225738 | -0.3438 | 42 |
| U34 | CGP-390 | chr17 | 74407 | 22188081 | -0.1239 | 192 |
| U34 | CGP-390 | chr17 | 25293732 | 78579545 | -0.1648 | 643 |
| U34 | CGP-390 | chr17 | 78579545 | 81100055 | -0.6311 | 59 |
| U34 | CGP-390 | chr18 | 91319 | 15321486 | 0.071 | 111 |
| U34 | CGP-390 | chr18 | 18528870 | 59117729 | 0.0649 | 394 |
| U34 | CGP-390 | chr18 | 59117729 | 59549039 | 0.502 | 15 |
| U34 | CGP-390 | chr18 | 59549039 | 78001503 | -0.0012 | 138 |
| U34 | CGP-390 | chr19 | 275349 | 490198 | 0.3577 | 2 |
| U34 | CGP-390 | chr19 | 490198 | 3034367 | -0.6469 | 61 |
| U34 | CGP-390 | chr19 | 3034367 | 15135892 | -0.1464 | 181 |
| U34 | CGP-390 | chr19 | 15135892 | 15347848 | 0.2699 | 37 |
| U34 | CGP-390 | chr19 | 15347848 | 24397443 | -0.1056 | 89 |
| U34 | CGP-390 | chr19 | 28283155 | 34546084 | 0.0386 | 60 |
| U34 | CGP-390 | chr19 | 34546084 | 43206470 | -0.2886 | 84 |
| U34 | CGP-390 | chr19 | 43206470 | 44267124 | 0.3049 | 8 |
| U34 | CGP-390 | chr19 | 44267124 | 45857291 | -0.1931 | 19 |
| U34 | CGP-390 | chr19 | 45857291 | 46031850 | 0.1664 | 18 |
| U34 | CGP-390 | chr19 | 46031850 | 59042307 | -0.2842 | 101 |
| U34 | CGP-390 | chr20 | 133209 | 26237717 | 0.013 | 190 |
| U34 | CGP-390 | chr20 | 29478404 | 62886880 | -0.1956 | 351 |
| U34 | CGP-390 | chr21 | 9464747 | 11106090 | 0.2174 | 10 |
| U34 | CGP-390 | chr21 | 14424106 | 48074396 | -0.0195 | 281 |
| U34 | CGP-390 | chr22 | 16131356 | 51186435 | -0.2434 | 485 |
| U34 | CGP-390 | chrX | 2709879 | 34567754 | 0.1375 | 232 |
| U34 | CGP-390 | chrX | 34567754 | 57873050 | -0.1213 | 270 |
| U34 | CGP-390 | chrX | 61931753 | 154882523 | 0.0769 | 765 |
| U34 | CGP-390 | chrY | 2654633 | 9535491 | -0.1682 | 43 |
| U34 | CGP-390 | chrY | 13199529 | 19586679 | 0.0049 | 37 |
| U34 | CGP-390 | chrY | 21033995 | 24521747 | 0.0426 | 18 |
| U34 | CGP-390 | chrY | 27495883 | 28590746 | -0.0742 | 6 |
| U33 | CGP-389 | chr1 | 93709 | 121286064 | -0.1177 | 1116 |
| U33 | CGP-389 | chr1 | 142633228 | 158854524 | -0.1654 | 156 |
| U33 | CGP-389 | chr1 | 158854524 | 164403658 | -0.8442 | 56 |
| U33 | CGP-389 | chr1 | 164403658 | 168486049 | 0.031 | 29 |
| U33 | CGP-389 | chr1 | 168486049 | 170701782 | 0.4886 | 16 |
| U33 | CGP-389 | chr1 | 170701782 | 173868806 | 0.0047 | 22 |
| U33 | CGP-389 | chr1 | 173868806 | 177154236 | -0.6886 | 25 |
| U33 | CGP-389 | chr1 | 177154236 | 186010832 | 0.0504 | 113 |
| U33 | CGP-389 | chr1 | 186010832 | 192157361 | -0.7322 | 42 |
| U33 | CGP-389 | chr1 | 192157361 | 209551776 | -0.0349 | 171 |
| U33 | CGP-389 | chr1 | 209551776 | 209963373 | 0.9684 | 3 |
| U33 | CGP-389 | chr1 | 209963373 | 220543853 | 0.0561 | 77 |
| U33 | CGP-389 | chr1 | 220543853 | 229365747 | -0.6266 | 63 |
| U33 | CGP-389 | chr1 | 229365747 | 230110159 | -1.314 | 5 |
| U33 | CGP-389 | chr1 | 230110159 | 232363647 | -0.5904 | 16 |
| U33 | CGP-389 | chr1 | 232363647 | 244047776 | -0.012 | 103 |
| U33 | CGP-389 | chr1 | 244047776 | 249153513 | 0.387 | 36 |
| U33 | CGP-389 | chr2 | 85990 | 90458314 | -0.0407 | 783 |
| U33 | CGP-389 | chr2 | 91668610 | 92252664 | 0.3398 | 5 |
| U33 | CGP-389 | chr2 | 95389401 | 243170925 | 0.0208 | 1231 |
| U33 | CGP-389 | chr3 | 133004 | 52279985 | -0.0115 | 471 |
| U33 | CGP-389 | chr3 | 52279985 | 52859057 | -0.4308 | 55 |
| U33 | CGP-389 | chr3 | 52859057 | 90264757 | 0.0523 | 313 |
| U33 | CGP-389 | chr3 | 93518046 | 197888970 | 0.0679 | 912 |
| U33 | CGP-389 | chr4 | 83904 | 3152602 | -0.2948 | 35 |
| U33 | CGP-389 | chr4 | 3152602 | 48595666 | 0.0216 | 328 |
| U33 | CGP-389 | chr4 | 48595666 | 49574529 | -0.4452 | 6 |
| U33 | CGP-389 | chr4 | 52678368 | 190976530 | 0.0882 | 1130 |
| U33 | CGP-389 | chr5 | 88178 | 3771661 | -0.2871 | 46 |
| U33 | CGP-389 | chr5 | 3771661 | 46176126 | 0.0404 | 348 |
| U33 | CGP-389 | chr5 | 49488207 | 69516698 | -0.0116 | 191 |
| U33 | CGP-389 | chr5 | 69516698 | 70108308 | -2.0749 | 3 |
| U33 | CGP-389 | chr5 | 70108308 | 149473313 | 0.0588 | 732 |
| U33 | CGP-389 | chr5 | 149473313 | 149550752 | -0.3858 | 38 |
| U33 | CGP-389 | chr5 | 149550752 | 180835789 | -0.0118 | 293 |
| U33 | CGP-389 | chr6 | 276952 | 28437090 | -0.0387 | 220 |
| U33 | CGP-389 | chr6 | 33418193 | 58710556 | 0.004 | 307 |
| U33 | CGP-389 | chr6 | 61948494 | 157231102 | 0.0726 | 851 |
| U33 | CGP-389 | chr6 | 157231102 | 160525907 | -0.264 | 105 |
| U33 | CGP-389 | chr6 | 160525907 | 170982158 | -0.013 | 78 |
| U33 | CGP-389 | chr7 | 121242 | 57974477 | 0.0103 | 511 |
| U33 | CGP-389 | chr7 | 61118627 | 98443585 | 0.1169 | 308 |
| U33 | CGP-389 | chr7 | 98443585 | 98643124 | -0.3384 | 71 |
| U33 | CGP-389 | chr7 | 98643124 | 159081217 | 0.0229 | 658 |
| U33 | CGP-389 | chr8 | 85562 | 43599480 | 0.0259 | 485 |
| U33 | CGP-389 | chr8 | 46894812 | 146224689 | 0.0358 | 841 |
| U33 | CGP-389 | chr9 | 85971 | 47238906 | 0.0518 | 486 |
| U33 | CGP-389 | chr9 | 65543126 | 133572959 | -0.0188 | 639 |
| U33 | CGP-389 | chr9 | 133572959 | 141079659 | -0.5358 | 138 |
| U33 | CGP-389 | chr10 | 132497 | 4466221 | -0.2316 | 32 |
| U33 | CGP-389 | chr10 | 4466221 | 38976463 | 0.0745 | 245 |
| U33 | CGP-389 | chr10 | 42701108 | 135442105 | -0.0375 | 798 |
| U33 | CGP-389 | chr11 | 139098 | 50401447 | -0.0146 | 382 |
| U33 | CGP-389 | chr11 | 51404035 | 51530482 | 0.1371 | 2 |
| U33 | CGP-389 | chr11 | 55028532 | 77943800 | -0.1671 | 196 |
| U33 | CGP-389 | chr11 | 77943800 | 117039724 | -0.7519 | 394 |
| U33 | CGP-389 | chr11 | 117039724 | 134873895 | -0.1289 | 223 |
| U33 | CGP-389 | chr12 | 77870 | 12007090 | -0.0195 | 124 |
| U33 | CGP-389 | chr12 | 12007090 | 12022434 | -0.3397 | 49 |
| U33 | CGP-389 | chr12 | 12022434 | 34611974 | 0.0182 | 202 |
| U33 | CGP-389 | chr12 | 37936846 | 133756626 | -0.0307 | 831 |
| U33 | CGP-389 | chr13 | 19104207 | 37068586 | 0.033 | 269 |
| U33 | CGP-389 | chr13 | 37068586 | 54619400 | -0.7668 | 154 |
| U33 | CGP-389 | chr13 | 54619400 | 115017146 | 0.0489 | 461 |
| U33 | CGP-389 | chr14 | 19110467 | 20439201 | -0.5383 | 18 |
| U33 | CGP-389 | chr14 | 20439201 | 102514263 | -0.0209 | 670 |
| U33 | CGP-389 | chr14 | 102514263 | 102556539 | -0.8003 | 13 |
| U33 | CGP-389 | chr14 | 102556539 | 107211129 | -0.2907 | 51 |
| U33 | CGP-389 | chr15 | 20098901 | 40552189 | -0.1122 | 219 |
| U33 | CGP-389 | chr15 | 40552189 | 42305296 | 0.1931 | 32 |
| U33 | CGP-389 | chr15 | 42305296 | 82860134 | -0.1579 | 322 |
| U33 | CGP-389 | chr15 | 82860134 | 83200197 | -1.6195 | 2 |
| U33 | CGP-389 | chr15 | 83200197 | 102435164 | -0.1185 | 201 |
| U33 | CGP-389 | chr16 | 133529 | 790789 | 0.0853 | 4 |
| U33 | CGP-389 | chr16 | 790789 | 3852085 | -0.543 | 104 |
| U33 | CGP-389 | chr16 | 3852085 | 31195922 | -0.1434 | 263 |
| U33 | CGP-389 | chr16 | 31195922 | 31240685 | -1.0156 | 14 |
| U33 | CGP-389 | chr16 | 31240685 | 35208244 | 0.174 | 28 |
| U33 | CGP-389 | chr16 | 46443761 | 90225738 | -0.1065 | 414 |
| U33 | CGP-389 | chr17 | 74407 | 22188081 | -0.1587 | 192 |
| U33 | CGP-389 | chr17 | 25293732 | 74117849 | -0.1819 | 610 |
| U33 | CGP-389 | chr17 | 74117849 | 81100055 | -0.5073 | 91 |
| U33 | CGP-389 | chr18 | 91319 | 15321486 | -0.0054 | 111 |
| U33 | CGP-389 | chr18 | 18528870 | 50591114 | 0.0322 | 282 |
| U33 | CGP-389 | chr18 | 50591114 | 51055009 | 0.3995 | 33 |
| U33 | CGP-389 | chr18 | 51055009 | 78009423 | 0.0335 | 233 |
| U33 | CGP-389 | chr19 | 275349 | 3430603 | -0.596 | 73 |
| U33 | CGP-389 | chr19 | 3430603 | 24397443 | -0.2121 | 297 |
| U33 | CGP-389 | chr19 | 28283155 | 59042307 | -0.2091 | 290 |
| U33 | CGP-389 | chr20 | 133209 | 26237717 | -0.0478 | 190 |
| U33 | CGP-389 | chr20 | 29478404 | 62886880 | -0.2395 | 353 |
| U33 | CGP-389 | chr21 | 9464747 | 11106090 | -0.1364 | 10 |
| U33 | CGP-389 | chr21 | 14424106 | 48074396 | -0.071 | 281 |
| U33 | CGP-389 | chr22 | 16131356 | 29666268 | -0.135 | 136 |
| U33 | CGP-389 | chr22 | 29666268 | 29694709 | -0.6088 | 56 |
| U33 | CGP-389 | chr22 | 29694709 | 39477303 | -0.2528 | 108 |
| U33 | CGP-389 | chr22 | 39477303 | 39637968 | -0.8186 | 29 |
| U33 | CGP-389 | chr22 | 39637968 | 51186435 | -0.3194 | 156 |
| U33 | CGP-389 | chrX | 2709879 | 57873050 | 0.0347 | 502 |
| U33 | CGP-389 | chrX | 61931753 | 154882523 | 0.0575 | 766 |
| U33 | CGP-389 | chrY | 2654633 | 9535491 | -0.1387 | 43 |
| U33 | CGP-389 | chrY | 13199529 | 19586679 | -0.0168 | 37 |
| U33 | CGP-389 | chrY | 21033995 | 24521747 | 0.0606 | 18 |
| U33 | CGP-389 | chrY | 27495883 | 28590746 | -0.2726 | 6 |
| U15 | CGP-337 | chr1 | 93709 | 45251691 | -0.0839 | 444 |
| U15 | CGP-337 | chr1 | 45251691 | 45805776 | 0.221 | 43 |
| U15 | CGP-337 | chr1 | 45805776 | 121286064 | -0.0797 | 629 |
| U15 | CGP-337 | chr1 | 142633228 | 149779198 | -0.0816 | 52 |
| U15 | CGP-337 | chr1 | 149779198 | 151950163 | -0.4648 | 22 |
| U15 | CGP-337 | chr1 | 151950163 | 249153513 | -0.0527 | 859 |
| U15 | CGP-337 | chr2 | 85990 | 90458314 | 0.4225 | 783 |
| U15 | CGP-337 | chr2 | 91668610 | 92252664 | 0.5641 | 5 |
| U15 | CGP-337 | chr2 | 95389401 | 243170925 | -0.0206 | 1230 |
| U15 | CGP-337 | chr3 | 133004 | 30607901 | -0.0235 | 266 |
| U15 | CGP-337 | chr3 | 30607901 | 30713669 | -0.716 | 9 |
| U15 | CGP-337 | chr3 | 30713669 | 90264757 | -0.0197 | 564 |
| U15 | CGP-337 | chr3 | 93518046 | 197888970 | -0.0169 | 912 |
| U15 | CGP-337 | chr4 | 83904 | 49574529 | -0.0442 | 369 |
| U15 | CGP-337 | chr4 | 52678368 | 79475820 | 0.0549 | 294 |
| U15 | CGP-337 | chr4 | 79475820 | 102752394 | -0.5768 | 168 |
| U15 | CGP-337 | chr4 | 102752394 | 190976530 | 0.0009 | 668 |
| U15 | CGP-337 | chr5 | 88178 | 46176126 | -0.013 | 394 |
| U15 | CGP-337 | chr5 | 49488207 | 180835789 | -0.0242 | 1257 |
| U15 | CGP-337 | chr6 | 276952 | 28586082 | -0.1025 | 221 |
| U15 | CGP-337 | chr6 | 33418193 | 58710556 | -0.029 | 307 |
| U15 | CGP-337 | chr6 | 61948494 | 155967114 | -0.0031 | 832 |
| U15 | CGP-337 | chr6 | 155967114 | 160517056 | -0.2517 | 120 |
| U15 | CGP-337 | chr6 | 160517056 | 170982158 | -0.0443 | 82 |
| U15 | CGP-337 | chr7 | 121242 | 57974477 | -0.0335 | 511 |
| U15 | CGP-337 | chr7 | 61118627 | 62462928 | -0.4763 | 4 |
| U15 | CGP-337 | chr7 | 62462928 | 159081217 | 0.0105 | 1034 |
| U15 | CGP-337 | chr8 | 85562 | 43599480 | 0.0135 | 485 |
| U15 | CGP-337 | chr8 | 46894812 | 146224689 | -0.0155 | 842 |
| U15 | CGP-337 | chr9 | 85971 | 42704433 | -0.0194 | 467 |
| U15 | CGP-337 | chr9 | 42704433 | 47238906 | 0.3079 | 20 |
| U15 | CGP-337 | chr9 | 65543126 | 141079659 | -0.0604 | 778 |
| U15 | CGP-337 | chr10 | 132497 | 38976463 | -0.0023 | 277 |
| U15 | CGP-337 | chr10 | 42701108 | 135442105 | -0.0237 | 798 |
| U15 | CGP-337 | chr11 | 139098 | 50401447 | -0.0457 | 382 |
| U15 | CGP-337 | chr11 | 51404035 | 51530482 | -0.0401 | 2 |
| U15 | CGP-337 | chr11 | 55028532 | 134873895 | -0.0555 | 813 |
| U15 | CGP-337 | chr12 | 77870 | 34611974 | -0.0761 | 375 |
| U15 | CGP-337 | chr12 | 37936846 | 133756626 | -0.0288 | 832 |
| U15 | CGP-337 | chr13 | 19104207 | 49810472 | -0.0554 | 388 |
| U15 | CGP-337 | chr13 | 49810472 | 51621518 | -1.2205 | 13 |
| U15 | CGP-337 | chr13 | 51621518 | 115017146 | -0.0368 | 483 |
| U15 | CGP-337 | chr14 | 19110467 | 106509277 | -0.0563 | 747 |
| U15 | CGP-337 | chr14 | 106509277 | 107211129 | -1.2284 | 5 |
| U15 | CGP-337 | chr15 | 20098901 | 102435164 | -0.0643 | 776 |
| U15 | CGP-337 | chr16 | 133529 | 31195922 | -0.0425 | 372 |
| U15 | CGP-337 | chr16 | 31195922 | 31363207 | -0.5899 | 15 |
| U15 | CGP-337 | chr16 | 31363207 | 35208244 | -0.0027 | 27 |
| U15 | CGP-337 | chr16 | 46443761 | 68731465 | -0.0838 | 200 |
| U15 | CGP-337 | chr16 | 68731465 | 68854626 | 0.3625 | 12 |
| U15 | CGP-337 | chr16 | 68854626 | 90225738 | -0.0349 | 202 |
| U15 | CGP-337 | chr17 | 74407 | 22188081 | -0.0454 | 192 |
| U15 | CGP-337 | chr17 | 25293732 | 37866239 | -0.0992 | 180 |
| U15 | CGP-337 | chr17 | 37866239 | 37883831 | 0.2332 | 21 |
| U15 | CGP-337 | chr17 | 37883831 | 81100055 | -0.0964 | 501 |
| U15 | CGP-337 | chr18 | 91319 | 15321486 | -0.7694 | 111 |
| U15 | CGP-337 | chr18 | 18528870 | 78009423 | -0.0055 | 548 |
| U15 | CGP-337 | chr19 | 275349 | 14843730 | 0.0124 | 241 |
| U15 | CGP-337 | chr19 | 14843730 | 15305791 | 0.3432 | 39 |
| U15 | CGP-337 | chr19 | 15305791 | 16831071 | -0.0528 | 12 |
| U15 | CGP-337 | chr19 | 16831071 | 17952384 | 0.2316 | 24 |
| U15 | CGP-337 | chr19 | 17952384 | 24397443 | -0.0151 | 54 |
| U15 | CGP-337 | chr19 | 28283155 | 45857291 | 0.0133 | 171 |
| U15 | CGP-337 | chr19 | 45857291 | 45873611 | 0.3379 | 16 |
| U15 | CGP-337 | chr19 | 45873611 | 59042307 | -0.022 | 103 |
| U15 | CGP-337 | chr20 | 133209 | 26237717 | -0.048 | 190 |
| U15 | CGP-337 | chr20 | 29478404 | 62886880 | -0.1241 | 353 |
| U15 | CGP-337 | chr21 | 9464747 | 11106090 | 0.3278 | 10 |
| U15 | CGP-337 | chr21 | 14424106 | 48074396 | -0.0542 | 281 |
| U15 | CGP-337 | chr22 | 16131356 | 51186435 | -0.0553 | 485 |
| U15 | CGP-337 | chrX | 2709879 | 57873050 | -0.0341 | 502 |
| U15 | CGP-337 | chrX | 61931753 | 154882523 | 0.0015 | 766 |
| U15 | CGP-337 | chrY | 2654633 | 9535491 | -0.2047 | 43 |
| U15 | CGP-337 | chrY | 13199529 | 19586679 | 0.0601 | 37 |
| U15 | CGP-337 | chrY | 21033995 | 24521747 | -0.0918 | 18 |
| U15 | CGP-337 | chrY | 27495883 | 28590746 | -0.0958 | 6 |
| U14 | CGP-336 | chr1 | 93709 | 11173687 | -0.0755 | 98 |
| U14 | CGP-336 | chr1 | 11173687 | 11185684 | 0.3066 | 7 |
| U14 | CGP-336 | chr1 | 11185684 | 45251691 | -0.0159 | 339 |
| U14 | CGP-336 | chr1 | 45251691 | 45805776 | 0.1908 | 43 |
| U14 | CGP-336 | chr1 | 45805776 | 121286064 | -0.0409 | 629 |
| U14 | CGP-336 | chr1 | 142633228 | 156177602 | -0.1326 | 104 |
| U14 | CGP-336 | chr1 | 156177602 | 156848904 | 0.23 | 35 |
| U14 | CGP-336 | chr1 | 156848904 | 249153513 | -0.0317 | 794 |
| U14 | CGP-336 | chr2 | 85990 | 47347822 | -0.0331 | 420 |
| U14 | CGP-336 | chr2 | 47347822 | 48024470 | 0.2524 | 27 |
| U14 | CGP-336 | chr2 | 48024470 | 89150242 | -0.0312 | 329 |
| U14 | CGP-336 | chr2 | 89150242 | 90458314 | -0.7647 | 7 |
| U14 | CGP-336 | chr2 | 91668610 | 92252664 | 0.2936 | 5 |
| U14 | CGP-336 | chr2 | 95389401 | 243170925 | -0.0235 | 1231 |
| U14 | CGP-336 | chr3 | 133004 | 30486962 | -0.0079 | 265 |
| U14 | CGP-336 | chr3 | 30486962 | 30726365 | -0.58 | 13 |
| U14 | CGP-336 | chr3 | 30726365 | 52397116 | -0.0183 | 194 |
| U14 | CGP-336 | chr3 | 52397116 | 52464387 | -0.402 | 18 |
| U14 | CGP-336 | chr3 | 52464387 | 90264757 | -0.0103 | 349 |
| U14 | CGP-336 | chr3 | 93518046 | 197888970 | -0.0282 | 912 |
| U14 | CGP-336 | chr4 | 83904 | 49574529 | -0.0216 | 369 |
| U14 | CGP-336 | chr4 | 52678368 | 190976530 | -0.006 | 1128 |
| U14 | CGP-336 | chr5 | 88178 | 46176126 | 0.005 | 394 |
| U14 | CGP-336 | chr5 | 49488207 | 180835789 | -0.0111 | 1257 |
| U14 | CGP-336 | chr6 | 276952 | 25969849 | -0.0068 | 202 |
| U14 | CGP-336 | chr6 | 25969849 | 26270028 | -1.0082 | 2 |
| U14 | CGP-336 | chr6 | 26270028 | 28586082 | -0.1745 | 17 |
| U14 | CGP-336 | chr6 | 33418193 | 58710556 | -0.0252 | 307 |
| U14 | CGP-336 | chr6 | 61948494 | 137262456 | -0.0149 | 688 |
| U14 | CGP-336 | chr6 | 137262456 | 138238791 | -0.3388 | 17 |
| U14 | CGP-336 | chr6 | 138238791 | 170982158 | -0.0999 | 329 |
| U14 | CGP-336 | chr7 | 121242 | 57974477 | -0.0157 | 511 |
| U14 | CGP-336 | chr7 | 61118627 | 159081217 | 0.0038 | 1038 |
| U14 | CGP-336 | chr8 | 85562 | 11705047 | 0.0119 | 118 |
| U14 | CGP-336 | chr8 | 11705047 | 12597732 | 0.5797 | 7 |
| U14 | CGP-336 | chr8 | 12597732 | 43599480 | 0.0167 | 360 |
| U14 | CGP-336 | chr8 | 46894812 | 146224689 | -0.0164 | 842 |
| U14 | CGP-336 | chr9 | 85971 | 39745541 | -0.0313 | 453 |
| U14 | CGP-336 | chr9 | 39745541 | 41534955 | 0.8246 | 7 |
| U14 | CGP-336 | chr9 | 41534955 | 47238906 | -0.0204 | 26 |
| U14 | CGP-336 | chr9 | 65543126 | 141079659 | -0.0564 | 778 |
| U14 | CGP-336 | chr10 | 132497 | 17827414 | -0.0175 | 128 |
| U14 | CGP-336 | chr10 | 17827414 | 18146942 | 1.0645 | 2 |
| U14 | CGP-336 | chr10 | 18146942 | 38976463 | 0.0409 | 147 |
| U14 | CGP-336 | chr10 | 42701108 | 135442105 | -0.0201 | 798 |
| U14 | CGP-336 | chr11 | 139098 | 50401447 | -0.012 | 382 |
| U14 | CGP-336 | chr11 | 51404035 | 51530482 | 0.0734 | 2 |
| U14 | CGP-336 | chr11 | 55028532 | 92721692 | -0.0212 | 309 |
| U14 | CGP-336 | chr11 | 92721692 | 116410933 | -0.7461 | 275 |
| U14 | CGP-336 | chr11 | 116410933 | 134873895 | -0.0444 | 228 |
| U14 | CGP-336 | chr12 | 77870 | 34611974 | -0.0475 | 375 |
| U14 | CGP-336 | chr12 | 37936846 | 133756626 | -0.0128 | 832 |
| U14 | CGP-336 | chr13 | 19104207 | 50573302 | -0.0042 | 393 |
| U14 | CGP-336 | chr13 | 50573302 | 51474185 | -1.0205 | 7 |
| U14 | CGP-336 | chr13 | 51474185 | 78481326 | -0.0297 | 200 |
| U14 | CGP-336 | chr13 | 78481326 | 79215402 | -0.4781 | 8 |
| U14 | CGP-336 | chr13 | 79215402 | 115017146 | -0.0226 | 276 |
| U14 | CGP-336 | chr14 | 19110467 | 107211129 | -0.0224 | 752 |
| U14 | CGP-336 | chr15 | 20098901 | 20239654 | -0.0843 | 2 |
| U14 | CGP-336 | chr15 | 20239654 | 22619373 | 0.7538 | 20 |
| U14 | CGP-336 | chr15 | 22619373 | 40943183 | 0.0144 | 200 |
| U14 | CGP-336 | chr15 | 40943183 | 42155446 | 0.2836 | 28 |
| U14 | CGP-336 | chr15 | 42155446 | 88104431 | -0.0278 | 360 |
| U14 | CGP-336 | chr15 | 88104431 | 88646216 | 0.3097 | 18 |
| U14 | CGP-336 | chr15 | 88646216 | 102435164 | -0.0731 | 148 |
| U14 | CGP-336 | chr16 | 133529 | 31165639 | 0.0171 | 371 |
| U14 | CGP-336 | chr16 | 31165639 | 31363207 | -0.4063 | 16 |
| U14 | CGP-336 | chr16 | 31363207 | 35208244 | 0.1571 | 27 |
| U14 | CGP-336 | chr16 | 46443761 | 68819803 | -0.0123 | 203 |
| U14 | CGP-336 | chr16 | 68819803 | 69166118 | 0.3756 | 17 |
| U14 | CGP-336 | chr16 | 69166118 | 90225738 | -0.012 | 194 |
| U14 | CGP-336 | chr17 | 74407 | 22188081 | 0.0048 | 192 |
| U14 | CGP-336 | chr17 | 25293732 | 43621848 | 0.042 | 311 |
| U14 | CGP-336 | chr17 | 43621848 | 44562730 | 0.6292 | 6 |
| U14 | CGP-336 | chr17 | 44562730 | 81100055 | -0.0448 | 385 |
| U14 | CGP-336 | chr18 | 91319 | 15321486 | 0.0293 | 111 |
| U14 | CGP-336 | chr18 | 18528870 | 78009423 | -0.0264 | 548 |
| U14 | CGP-336 | chr19 | 275349 | 3546764 | -0.1757 | 74 |
| U14 | CGP-336 | chr19 | 3546764 | 7120102 | 0.0339 | 45 |
| U14 | CGP-336 | chr19 | 7120102 | 7246721 | 0.4439 | 19 |
| U14 | CGP-336 | chr19 | 7246721 | 15237486 | 0.0683 | 107 |
| U14 | CGP-336 | chr19 | 15237486 | 15305791 | 0.3946 | 35 |
| U14 | CGP-336 | chr19 | 15305791 | 24397443 | 0.1039 | 90 |
| U14 | CGP-336 | chr19 | 28283155 | 59042307 | 0.0557 | 290 |
| U14 | CGP-336 | chr20 | 133209 | 26237717 | -0.051 | 190 |
| U14 | CGP-336 | chr20 | 29478404 | 62886880 | -0.0777 | 353 |
| U14 | CGP-336 | chr21 | 9464747 | 11106090 | 0.1592 | 10 |
| U14 | CGP-336 | chr21 | 14424106 | 48074396 | -0.0583 | 281 |
| U14 | CGP-336 | chr22 | 16131356 | 39477303 | 0.0084 | 300 |
| U14 | CGP-336 | chr22 | 39477303 | 39637968 | -0.3339 | 29 |
| U14 | CGP-336 | chr22 | 39637968 | 51186435 | -0.006 | 156 |
| U14 | CGP-336 | chrX | 2709879 | 57873050 | -0.0342 | 502 |
| U14 | CGP-336 | chrX | 61931753 | 154882523 | -0.0093 | 766 |
| U14 | CGP-336 | chrY | 2654633 | 9535491 | -0.1238 | 43 |
| U14 | CGP-336 | chrY | 13199529 | 19586679 | -0.1252 | 37 |
| U14 | CGP-336 | chrY | 21033995 | 24521747 | 0.13 | 18 |
| U14 | CGP-336 | chrY | 27495883 | 28590746 | -0.0537 | 6 |
| E20 | CGP-454 | chr1 | 93709 | 97522893 | 0.0368 | 893 |
| E20 | CGP-454 | chr1 | 97522893 | 98514340 | -2.1061 | 11 |
| E20 | CGP-454 | chr1 | 98514340 | 121286064 | -0.1213 | 212 |
| E20 | CGP-454 | chr1 | 142633228 | 249153513 | -0.0987 | 933 |
| E20 | CGP-454 | chr2 | 85990 | 90458314 | -0.0565 | 783 |
| E20 | CGP-454 | chr2 | 91668610 | 92252664 | -1.6004 | 5 |
| E20 | CGP-454 | chr2 | 95389401 | 140865672 | -0.1597 | 355 |
| E20 | CGP-454 | chr2 | 140865672 | 142061721 | -3.7181 | 8 |
| E20 | CGP-454 | chr2 | 142061721 | 243170925 | -0.1945 | 868 |
| E20 | CGP-454 | chr3 | 133004 | 70970982 | -0.0458 | 677 |
| E20 | CGP-454 | chr3 | 70970982 | 71274268 | -3.3984 | 2 |
| E20 | CGP-454 | chr3 | 71274268 | 90264757 | -0.1403 | 160 |
| E20 | CGP-454 | chr3 | 93518046 | 189304320 | -0.1911 | 849 |
| E20 | CGP-454 | chr3 | 189304320 | 189754617 | -3.5405 | 3 |
| E20 | CGP-454 | chr3 | 189754617 | 197888970 | -0.2711 | 60 |
| E20 | CGP-454 | chr4 | 83904 | 49574529 | -0.0602 | 369 |
| E20 | CGP-454 | chr4 | 52678368 | 108908279 | -0.1046 | 517 |
| E20 | CGP-454 | chr4 | 108908279 | 109204938 | -3.3582 | 2 |
| E20 | CGP-454 | chr4 | 109204938 | 190976530 | -0.1383 | 611 |
| E20 | CGP-454 | chr5 | 88178 | 46176126 | -0.1501 | 394 |
| E20 | CGP-454 | chr5 | 49488207 | 158102723 | -0.1477 | 1027 |
| E20 | CGP-454 | chr5 | 158102723 | 158552330 | -3.157 | 3 |
| E20 | CGP-454 | chr5 | 158552330 | 170817719 | -0.0343 | 92 |
| E20 | CGP-454 | chr5 | 170817719 | 170879068 | 0.8381 | 16 |
| E20 | CGP-454 | chr5 | 170879068 | 176566583 | 0.023 | 57 |
| E20 | CGP-454 | chr5 | 176566583 | 176858618 | -2.7782 | 2 |
| E20 | CGP-454 | chr5 | 176858618 | 179970249 | -0.0002 | 23 |
| E20 | CGP-454 | chr5 | 179970249 | 180063168 | 0.3019 | 31 |
| E20 | CGP-454 | chr5 | 180063168 | 180835789 | -0.2062 | 6 |
| E20 | CGP-454 | chr6 | 276952 | 25969849 | -0.0415 | 202 |
| E20 | CGP-454 | chr6 | 25969849 | 26420118 | -3.3987 | 3 |
| E20 | CGP-454 | chr6 | 26420118 | 28586082 | -0.2593 | 16 |
| E20 | CGP-454 | chr6 | 33418193 | 58710556 | -0.1326 | 307 |
| E20 | CGP-454 | chr6 | 61948494 | 152002032 | -0.2063 | 804 |
| E20 | CGP-454 | chr6 | 152002032 | 152446573 | -3.4134 | 3 |
| E20 | CGP-454 | chr6 | 152446573 | 170982158 | -0.1932 | 227 |
| E20 | CGP-454 | chr7 | 121242 | 18152652 | -0.0329 | 162 |
| E20 | CGP-454 | chr7 | 18152652 | 19053551 | -3.3253 | 6 |
| E20 | CGP-454 | chr7 | 19053551 | 27752599 | -0.192 | 68 |
| E20 | CGP-454 | chr7 | 27752599 | 28354402 | -3.1241 | 4 |
| E20 | CGP-454 | chr7 | 28354402 | 57974477 | -0.1725 | 271 |
| E20 | CGP-454 | chr7 | 61118627 | 151811490 | -0.156 | 987 |
| E20 | CGP-454 | chr7 | 151811490 | 152417286 | -2.8961 | 4 |
| E20 | CGP-454 | chr7 | 152417286 | 159081217 | -0.181 | 47 |
| E20 | CGP-454 | chr8 | 85562 | 43599480 | -0.0791 | 485 |
| E20 | CGP-454 | chr8 | 46894812 | 92932731 | -0.075 | 416 |
| E20 | CGP-454 | chr8 | 92932731 | 93246201 | -1.0009 | 16 |
| E20 | CGP-454 | chr8 | 93246201 | 146224689 | -0.0832 | 410 |
| E20 | CGP-454 | chr9 | 85971 | 47238906 | -0.1923 | 487 |
| E20 | CGP-454 | chr9 | 65543126 | 141079659 | -0.0233 | 778 |
| E20 | CGP-454 | chr10 | 132497 | 7959302 | -0.0282 | 57 |
| E20 | CGP-454 | chr10 | 7959302 | 8258556 | -3.5002 | 2 |
| E20 | CGP-454 | chr10 | 8258556 | 38976463 | -0.0473 | 218 |
| E20 | CGP-454 | chr10 | 42701108 | 63603781 | -0.0688 | 176 |
| E20 | CGP-454 | chr10 | 63603781 | 63904417 | -3.7448 | 2 |
| E20 | CGP-454 | chr10 | 63904417 | 114604780 | -0.1022 | 445 |
| E20 | CGP-454 | chr10 | 114604780 | 115055005 | -3.2944 | 3 |
| E20 | CGP-454 | chr10 | 115055005 | 135442105 | -0.0824 | 172 |
| E20 | CGP-454 | chr11 | 139098 | 50401447 | -0.0806 | 382 |
| E20 | CGP-454 | chr11 | 51404035 | 51530482 | -0.8387 | 2 |
| E20 | CGP-454 | chr11 | 55028532 | 134873895 | -0.0671 | 813 |
| E20 | CGP-454 | chr12 | 77870 | 34611974 | -0.1789 | 375 |
| E20 | CGP-454 | chr12 | 37936846 | 49561665 | -0.2635 | 116 |
| E20 | CGP-454 | chr12 | 49561665 | 78157985 | 0.4138 | 278 |
| E20 | CGP-454 | chr12 | 78157985 | 78608829 | -3.2067 | 3 |
| E20 | CGP-454 | chr12 | 78608829 | 112863713 | 0.3263 | 258 |
| E20 | CGP-454 | chr12 | 112863713 | 112980724 | 1.1207 | 17 |
| E20 | CGP-454 | chr12 | 112980724 | 133756626 | 0.4012 | 160 |
| E20 | CGP-454 | chr13 | 19104207 | 28566521 | -0.2406 | 100 |
| E20 | CGP-454 | chr13 | 28566521 | 28925686 | 0.405 | 40 |
| E20 | CGP-454 | chr13 | 28925686 | 33163254 | -0.0488 | 100 |
| E20 | CGP-454 | chr13 | 33163254 | 33462329 | -3.5587 | 2 |
| E20 | CGP-454 | chr13 | 33462329 | 78637654 | -0.4011 | 362 |
| E20 | CGP-454 | chr13 | 78637654 | 115017146 | -0.1189 | 280 |
| E20 | CGP-454 | chr14 | 19110467 | 106352640 | -0.0378 | 746 |
| E20 | CGP-454 | chr14 | 106352640 | 107211129 | -2.151 | 6 |
| E20 | CGP-454 | chr15 | 20098901 | 102435164 | 0.0038 | 776 |
| E20 | CGP-454 | chr16 | 133529 | 31240685 | 0.2462 | 386 |
| E20 | CGP-454 | chr16 | 31240685 | 35208244 | -0.3624 | 28 |
| E20 | CGP-454 | chr16 | 46443761 | 90225738 | 0.0458 | 414 |
| E20 | CGP-454 | chr17 | 74407 | 22188081 | -0.1348 | 192 |
| E20 | CGP-454 | chr17 | 25293732 | 81100055 | 0.0017 | 702 |
| E20 | CGP-454 | chr18 | 91319 | 15321486 | -0.1224 | 111 |
| E20 | CGP-454 | chr18 | 18528870 | 42156109 | -0.1071 | 191 |
| E20 | CGP-454 | chr18 | 42156109 | 42754349 | -3.2224 | 4 |
| E20 | CGP-454 | chr18 | 42754349 | 78009423 | -0.1824 | 353 |
| E20 | CGP-454 | chr19 | 275349 | 24397443 | 0.3365 | 370 |
| E20 | CGP-454 | chr19 | 28283155 | 59042307 | 0.117 | 290 |
| E20 | CGP-454 | chr20 | 133209 | 26237717 | -0.0115 | 190 |
| E20 | CGP-454 | chr20 | 29478404 | 40653188 | 0.021 | 151 |
| E20 | CGP-454 | chr20 | 40653188 | 41551517 | -2.521 | 6 |
| E20 | CGP-454 | chr20 | 41551517 | 62886880 | -0.0306 | 196 |
| E20 | CGP-454 | chr21 | 9464747 | 11106090 | -0.4651 | 10 |
| E20 | CGP-454 | chr21 | 14424106 | 48074396 | -0.1253 | 281 |
| E20 | CGP-454 | chr22 | 16131356 | 51186435 | 0.234 | 485 |
| E20 | CGP-454 | chrX | 2709879 | 53147034 | -0.1143 | 467 |
| E20 | CGP-454 | chrX | 53147034 | 53577363 | -2.9877 | 3 |
| E20 | CGP-454 | chrX | 53577363 | 57873050 | 0.0316 | 32 |
| E20 | CGP-454 | chrX | 61931753 | 76707518 | -0.209 | 141 |
| E20 | CGP-454 | chrX | 76707518 | 77136372 | -3.11 | 3 |
| E20 | CGP-454 | chrX | 77136372 | 154882523 | -0.1106 | 622 |
| E20 | CGP-454 | chrY | 2654633 | 9535491 | -0.126 | 43 |
| E20 | CGP-454 | chrY | 13199529 | 19586679 | -0.0734 | 37 |
| E14 | CGP-449 | chrY | 21033995 | 24521747 | -0.0474 | 18 |
| E14 | CGP-449 | chrY | 27495883 | 28590746 | -0.7366 | 6 |
| E14 | CGP-449 | chr1 | 93709 | 97522893 | 0.0291 | 893 |
| E14 | CGP-449 | chr1 | 97522893 | 98514340 | -1.921 | 11 |
| E14 | CGP-449 | chr1 | 98514340 | 121286064 | -0.0674 | 212 |
| E14 | CGP-449 | chr1 | 142633228 | 145394502 | -0.0937 | 17 |
| E14 | CGP-449 | chr1 | 145394502 | 180755979 | 0.4886 | 331 |
| E14 | CGP-449 | chr1 | 180755979 | 222609975 | 0.0031 | 377 |
| E14 | CGP-449 | chr1 | 222609975 | 244952637 | -0.8879 | 179 |
| E14 | CGP-449 | chr1 | 244952637 | 249153513 | -0.0299 | 29 |
| E14 | CGP-449 | chr2 | 85990 | 25833941 | -0.0028 | 219 |
| E14 | CGP-449 | chr2 | 25833941 | 26130491 | -3.0312 | 2 |
| E14 | CGP-449 | chr2 | 26130491 | 39165672 | 0.0936 | 139 |
| E14 | CGP-449 | chr2 | 39165672 | 39613012 | -2.8073 | 3 |
| E14 | CGP-449 | chr2 | 39613012 | 90458314 | 0.045 | 420 |
| E14 | CGP-449 | chr2 | 91668610 | 92252664 | -1.3904 | 5 |
| E14 | CGP-449 | chr2 | 95389401 | 140865672 | -0.1311 | 355 |
| E14 | CGP-449 | chr2 | 140865672 | 142061721 | -3.5258 | 8 |
| E14 | CGP-449 | chr2 | 142061721 | 243170925 | -0.1512 | 868 |
| E14 | CGP-449 | chr3 | 133004 | 70970982 | -0.0322 | 677 |
| E14 | CGP-449 | chr3 | 70970982 | 71274268 | -3.4684 | 2 |
| E14 | CGP-449 | chr3 | 71274268 | 90264757 | -0.1464 | 160 |
| E14 | CGP-449 | chr3 | 93518046 | 119405608 | -0.1709 | 222 |
| E14 | CGP-449 | chr3 | 119405608 | 119853435 | -3.0318 | 3 |
| E14 | CGP-449 | chr3 | 119853435 | 168751842 | -0.1175 | 439 |
| E14 | CGP-449 | chr3 | 168751842 | 169462806 | -2.4716 | 5 |
| E14 | CGP-449 | chr3 | 169462806 | 189304320 | -0.1849 | 180 |
| E14 | CGP-449 | chr3 | 189304320 | 189754617 | -3.4347 | 3 |
| E14 | CGP-449 | chr3 | 189754617 | 197888970 | -0.2185 | 60 |
| E14 | CGP-449 | chr4 | 83904 | 49574529 | -0.1148 | 369 |
| E14 | CGP-449 | chr4 | 52678368 | 108908279 | -0.0902 | 517 |
| E14 | CGP-449 | chr4 | 108908279 | 109204938 | -3.4132 | 2 |
| E14 | CGP-449 | chr4 | 109204938 | 190976530 | -0.1456 | 611 |
| E14 | CGP-449 | chr5 | 88178 | 46176126 | -0.1784 | 394 |
| E14 | CGP-449 | chr5 | 49488207 | 158102723 | -0.1144 | 1027 |
| E14 | CGP-449 | chr5 | 158102723 | 158552330 | -3.1466 | 3 |
| E14 | CGP-449 | chr5 | 158552330 | 170818499 | -0.0086 | 93 |
| E14 | CGP-449 | chr5 | 170818499 | 170879068 | 0.5706 | 15 |
| E14 | CGP-449 | chr5 | 170879068 | 176566583 | 0.0116 | 57 |
| E14 | CGP-449 | chr5 | 176566583 | 176858618 | -2.5351 | 2 |
| E14 | CGP-449 | chr5 | 176858618 | 180835789 | 0.0486 | 60 |
| E14 | CGP-449 | chr6 | 276952 | 25969849 | -0.0377 | 202 |
| E14 | CGP-449 | chr6 | 25969849 | 26420118 | -3.3984 | 3 |
| E14 | CGP-449 | chr6 | 26420118 | 28586082 | -0.2346 | 16 |
| E14 | CGP-449 | chr6 | 33418193 | 58710556 | -0.0995 | 307 |
| E14 | CGP-449 | chr6 | 61948494 | 152002032 | -0.1794 | 804 |
| E14 | CGP-449 | chr6 | 152002032 | 152446573 | -3.43 | 3 |
| E14 | CGP-449 | chr6 | 152446573 | 170982158 | -0.2002 | 227 |
| E14 | CGP-449 | chr7 | 121242 | 18152652 | -0.061 | 162 |
| E14 | CGP-449 | chr7 | 18152652 | 19053551 | -3.2775 | 6 |
| E14 | CGP-449 | chr7 | 19053551 | 27752599 | -0.1561 | 68 |
| E14 | CGP-449 | chr7 | 27752599 | 28354402 | -3.2439 | 4 |
| E14 | CGP-449 | chr7 | 28354402 | 57974477 | -0.1891 | 271 |
| E14 | CGP-449 | chr7 | 61118627 | 151811490 | -0.1417 | 987 |
| E14 | CGP-449 | chr7 | 151811490 | 152417286 | -2.8483 | 4 |
| E14 | CGP-449 | chr7 | 152417286 | 159081217 | -0.2116 | 47 |
| E14 | CGP-449 | chr8 | 85562 | 43599480 | -0.1999 | 485 |
| E14 | CGP-449 | chr8 | 46894812 | 146224689 | -0.1032 | 842 |
| E14 | CGP-449 | chr9 | 85971 | 47238906 | -0.1631 | 487 |
| E14 | CGP-449 | chr9 | 65543126 | 141079659 | -0.0551 | 778 |
| E14 | CGP-449 | chr10 | 132497 | 38976463 | -0.0922 | 277 |
| E14 | CGP-449 | chr10 | 42701108 | 63603781 | -0.1266 | 175 |
| E14 | CGP-449 | chr10 | 63603781 | 63904417 | -3.6899 | 2 |
| E14 | CGP-449 | chr10 | 63904417 | 114604780 | -0.0752 | 445 |
| E14 | CGP-449 | chr10 | 114604780 | 115055005 | -3.2646 | 3 |
| E14 | CGP-449 | chr10 | 115055005 | 135442105 | -0.0459 | 172 |
| E14 | CGP-449 | chr11 | 139098 | 50401447 | -0.0996 | 382 |
| E14 | CGP-449 | chr11 | 51404035 | 51530482 | -0.8569 | 2 |
| E14 | CGP-449 | chr11 | 55028532 | 134873895 | -0.0349 | 813 |
| E14 | CGP-449 | chr12 | 77870 | 9757618 | -0.1933 | 101 |
| E14 | CGP-449 | chr12 | 9757618 | 12009266 | -2.6676 | 27 |
| E14 | CGP-449 | chr12 | 12009266 | 12013489 | -0.0318 | 15 |
| E14 | CGP-449 | chr12 | 12013489 | 12116903 | -2.7938 | 30 |
| E14 | CGP-449 | chr12 | 12116903 | 34611974 | -0.0856 | 199 |
| E14 | CGP-449 | chr12 | 37936846 | 78157985 | -0.029 | 394 |
| E14 | CGP-449 | chr12 | 78157985 | 78608829 | -3.4124 | 3 |
| E14 | CGP-449 | chr12 | 78608829 | 133756626 | 0.1163 | 435 |
| E14 | CGP-449 | chr13 | 19104207 | 115017146 | -0.1246 | 884 |
| E14 | CGP-449 | chr14 | 19110467 | 106352640 | -0.0146 | 746 |
| E14 | CGP-449 | chr14 | 106352640 | 107211129 | -2.7495 | 5 |
| E14 | CGP-449 | chr15 | 20098901 | 102435164 | -0.0064 | 776 |
| E14 | CGP-449 | chr16 | 133529 | 1484857 | -0.5781 | 9 |
| E14 | CGP-449 | chr16 | 1484857 | 31240685 | 0.1996 | 377 |
| E14 | CGP-449 | chr16 | 31240685 | 35208244 | -0.3041 | 28 |
| E14 | CGP-449 | chr16 | 46443761 | 90225738 | 0.0099 | 414 |
| E14 | CGP-449 | chr17 | 74407 | 22188081 | -0.1468 | 192 |
| E14 | CGP-449 | chr17 | 25293732 | 81100055 | -0.0269 | 702 |
| E14 | CGP-449 | chr18 | 91319 | 15321486 | -0.1999 | 111 |
| E14 | CGP-449 | chr18 | 18528870 | 42156109 | -0.1325 | 191 |
| E14 | CGP-449 | chr18 | 42156109 | 42754349 | -3.3278 | 4 |
| E14 | CGP-449 | chr18 | 42754349 | 78009423 | -0.1618 | 353 |
| E14 | CGP-449 | chr19 | 275349 | 24397443 | 0.2258 | 370 |
| E14 | CGP-449 | chr19 | 28283155 | 59042307 | 0.0636 | 290 |
| E14 | CGP-449 | chr20 | 133209 | 26237717 | -0.0624 | 190 |
| E14 | CGP-449 | chr20 | 29478404 | 40653188 | 0.0017 | 151 |
| E14 | CGP-449 | chr20 | 40653188 | 41551517 | -2.5027 | 6 |
| E14 | CGP-449 | chr20 | 41551517 | 62886880 | -0.2952 | 196 |
| E14 | CGP-449 | chr21 | 9464747 | 11106090 | -0.8308 | 10 |
| E14 | CGP-449 | chr21 | 14424106 | 48074396 | -0.142 | 281 |
| E14 | CGP-449 | chr22 | 16131356 | 51186435 | 0.1548 | 485 |
| E14 | CGP-449 | chrX | 2709879 | 53147034 | -0.1001 | 467 |
| E14 | CGP-449 | chrX | 53147034 | 53577363 | -2.9457 | 3 |
| E14 | CGP-449 | chrX | 53577363 | 57873050 | 0.0417 | 32 |
| E14 | CGP-449 | chrX | 61931753 | 76707518 | -0.1765 | 141 |
| E14 | CGP-449 | chrX | 76707518 | 77136372 | -3.0054 | 3 |
| E14 | CGP-449 | chrX | 77136372 | 154882523 | -0.1071 | 622 |
| E14 | CGP-449 | chrY | 2654633 | 9535491 | -0.2106 | 43 |
| E14 | CGP-449 | chrY | 13199529 | 19586679 | -0.1853 | 37 |
| E14 | CGP-449 | chrY | 21033995 | 24521747 | -0.1275 | 18 |
| E14 | CGP-449 | chrY | 27495883 | 28590746 | -0.599 | 6 |
| E10 | CGP-447 | chr1 | 93709 | 51400142 | 0.0757 | 529 |
| E10 | CGP-447 | chr1 | 51400142 | 51478178 | 8.108 | 3 |
| E10 | CGP-447 | chr1 | 51478178 | 97522893 | -0.0699 | 361 |
| E10 | CGP-447 | chr1 | 97522893 | 98514340 | -2.0678 | 11 |
| E10 | CGP-447 | chr1 | 98514340 | 121286064 | -0.1189 | 212 |
| E10 | CGP-447 | chr1 | 142633228 | 249153513 | -0.0859 | 933 |
| E10 | CGP-447 | chr2 | 85990 | 25833941 | -0.0693 | 219 |
| E10 | CGP-447 | chr2 | 25833941 | 26130491 | -3.1437 | 2 |
| E10 | CGP-447 | chr2 | 26130491 | 39165672 | 0.0085 | 139 |
| E10 | CGP-447 | chr2 | 39165672 | 39613012 | -2.8034 | 3 |
| E10 | CGP-447 | chr2 | 39613012 | 59101429 | 0.04 | 182 |
| E10 | CGP-447 | chr2 | 59101429 | 61661961 | 0.6176 | 31 |
| E10 | CGP-447 | chr2 | 61661961 | 90458314 | -0.051 | 207 |
| E10 | CGP-447 | chr2 | 91668610 | 92252664 | -1.6007 | 5 |
| E10 | CGP-447 | chr2 | 95389401 | 140865672 | -0.1982 | 355 |
| E10 | CGP-447 | chr2 | 140865672 | 142958757 | -2.8791 | 14 |
| E10 | CGP-447 | chr2 | 142958757 | 243170925 | -0.2046 | 862 |
| E10 | CGP-447 | chr3 | 133004 | 90264757 | -0.0933 | 839 |
| E10 | CGP-447 | chr3 | 93518046 | 119405608 | -0.2196 | 222 |
| E10 | CGP-447 | chr3 | 119405608 | 119853435 | -3.2053 | 3 |
| E10 | CGP-447 | chr3 | 119853435 | 168751842 | -0.1599 | 439 |
| E10 | CGP-447 | chr3 | 168751842 | 169462806 | -2.6406 | 5 |
| E10 | CGP-447 | chr3 | 169462806 | 189304320 | -0.2405 | 180 |
| E10 | CGP-447 | chr3 | 189304320 | 189754617 | -3.7078 | 3 |
| E10 | CGP-447 | chr3 | 189754617 | 197888970 | -0.2889 | 60 |
| E10 | CGP-447 | chr4 | 83904 | 49574529 | -0.1432 | 369 |
| E10 | CGP-447 | chr4 | 52678368 | 190976530 | -0.1581 | 1130 |
| E10 | CGP-447 | chr5 | 88178 | 46176126 | -0.1858 | 394 |
| E10 | CGP-447 | chr5 | 49488207 | 158102723 | -0.1836 | 1026 |
| E10 | CGP-447 | chr5 | 158102723 | 158552330 | -3.4554 | 3 |
| E10 | CGP-447 | chr5 | 158552330 | 176566583 | -0.0162 | 165 |
| E10 | CGP-447 | chr5 | 176566583 | 176858618 | -2.6691 | 2 |
| E10 | CGP-447 | chr5 | 176858618 | 180352988 | 0.0708 | 57 |
| E10 | CGP-447 | chr5 | 180352988 | 180835789 | -0.9409 | 3 |
| E10 | CGP-447 | chr6 | 276952 | 25969849 | -0.0083 | 202 |
| E10 | CGP-447 | chr6 | 25969849 | 26420118 | -3.2796 | 3 |
| E10 | CGP-447 | chr6 | 26420118 | 28586082 | -0.2373 | 16 |
| E10 | CGP-447 | chr6 | 33418193 | 58710556 | -0.1327 | 307 |
| E10 | CGP-447 | chr6 | 61948494 | 152002032 | -0.2185 | 804 |
| E10 | CGP-447 | chr6 | 152002032 | 152446573 | -3.7881 | 3 |
| E10 | CGP-447 | chr6 | 152446573 | 170982158 | -0.2073 | 227 |
| E10 | CGP-447 | chr7 | 121242 | 18152652 | -0.0823 | 162 |
| E10 | CGP-447 | chr7 | 18152652 | 19053551 | -3.5724 | 6 |
| E10 | CGP-447 | chr7 | 19053551 | 27752599 | -0.2475 | 68 |
| E10 | CGP-447 | chr7 | 27752599 | 28354402 | -3.2597 | 4 |
| E10 | CGP-447 | chr7 | 28354402 | 57974477 | -0.2143 | 271 |
| E10 | CGP-447 | chr7 | 61118627 | 101415617 | -0.1921 | 431 |
| E10 | CGP-447 | chr7 | 101415617 | 102025261 | -2.8157 | 4 |
| E10 | CGP-447 | chr7 | 102025261 | 151811490 | -0.1735 | 552 |
| E10 | CGP-447 | chr7 | 151811490 | 152417286 | -2.9306 | 4 |
| E10 | CGP-447 | chr7 | 152417286 | 159081217 | -0.2278 | 47 |
| E10 | CGP-447 | chr8 | 85562 | 43599480 | -0.1431 | 485 |
| E10 | CGP-447 | chr8 | 46894812 | 146224689 | -0.1396 | 842 |
| E10 | CGP-447 | chr9 | 85971 | 21969640 | -0.1415 | 308 |
| E10 | CGP-447 | chr9 | 21969640 | 21984470 | 5.437 | 3 |
| E10 | CGP-447 | chr9 | 21984470 | 47238906 | -0.2762 | 175 |
| E10 | CGP-447 | chr9 | 65543126 | 141079659 | -0.0371 | 778 |
| E10 | CGP-447 | chr10 | 132497 | 7959302 | -0.0582 | 57 |
| E10 | CGP-447 | chr10 | 7959302 | 8258556 | -3.8843 | 2 |
| E10 | CGP-447 | chr10 | 8258556 | 38976463 | -0.0853 | 218 |
| E10 | CGP-447 | chr10 | 42701108 | 63603781 | -0.1411 | 176 |
| E10 | CGP-447 | chr10 | 63603781 | 63904417 | -3.7998 | 2 |
| E10 | CGP-447 | chr10 | 63904417 | 114604780 | -0.1201 | 445 |
| E10 | CGP-447 | chr10 | 114604780 | 115055005 | -3.5366 | 3 |
| E10 | CGP-447 | chr10 | 115055005 | 135442105 | -0.0955 | 172 |
| E10 | CGP-447 | chr11 | 139098 | 50401447 | -0.1283 | 382 |
| E10 | CGP-447 | chr11 | 51404035 | 51530482 | -0.6134 | 2 |
| E10 | CGP-447 | chr11 | 55028532 | 134873895 | -0.0655 | 813 |
| E10 | CGP-447 | chr12 | 77870 | 34611974 | -0.2078 | 375 |
| E10 | CGP-447 | chr12 | 37936846 | 78157985 | -0.1066 | 394 |
| E10 | CGP-447 | chr12 | 78157985 | 78608829 | -3.7881 | 3 |
| E10 | CGP-447 | chr12 | 78608829 | 133756626 | -0.0262 | 435 |
| E10 | CGP-447 | chr13 | 19104207 | 47403814 | -0.1298 | 342 |
| E10 | CGP-447 | chr13 | 47403814 | 52808982 | -0.837 | 68 |
| E10 | CGP-447 | chr13 | 52808982 | 115017146 | -0.1956 | 474 |
| E10 | CGP-447 | chr14 | 19110467 | 20707693 | -0.8509 | 20 |
| E10 | CGP-447 | chr14 | 20707693 | 106352640 | -0.0649 | 726 |
| E10 | CGP-447 | chr14 | 106352640 | 107211129 | -1.4467 | 6 |
| E10 | CGP-447 | chr15 | 20098901 | 102435164 | 0.0165 | 776 |
| E10 | CGP-447 | chr16 | 133529 | 1484857 | -0.8695 | 9 |
| E10 | CGP-447 | chr16 | 1484857 | 31240685 | 0.2004 | 377 |
| E10 | CGP-447 | chr16 | 31240685 | 35208244 | -0.3753 | 28 |
| E10 | CGP-447 | chr16 | 46443761 | 90225738 | 0.0175 | 414 |
| E10 | CGP-447 | chr17 | 74407 | 22188081 | -0.7887 | 192 |
| E10 | CGP-447 | chr17 | 25293732 | 81100055 | -0.0033 | 702 |
| E10 | CGP-447 | chr18 | 91319 | 15321486 | -0.1866 | 111 |
| E10 | CGP-447 | chr18 | 18528870 | 42156109 | -0.1338 | 191 |
| E10 | CGP-447 | chr18 | 42156109 | 42754349 | -3.5323 | 4 |
| E10 | CGP-447 | chr18 | 42754349 | 78009423 | -0.1958 | 353 |
| E10 | CGP-447 | chr19 | 275349 | 24397443 | 0.205 | 370 |
| E10 | CGP-447 | chr19 | 28283155 | 59042307 | 0.0826 | 290 |
| E10 | CGP-447 | chr20 | 133209 | 26237717 | -0.0437 | 190 |
| E10 | CGP-447 | chr20 | 29478404 | 40653188 | -0.0032 | 151 |
| E10 | CGP-447 | chr20 | 40653188 | 41551517 | -2.7131 | 6 |
| E10 | CGP-447 | chr20 | 41551517 | 62886880 | -0.0628 | 196 |
| E10 | CGP-447 | chr21 | 9464747 | 11106090 | -0.484 | 10 |
| E10 | CGP-447 | chr21 | 14424106 | 48074396 | -0.1174 | 281 |
| E10 | CGP-447 | chr22 | 16131356 | 22053660 | -0.0062 | 59 |
| E10 | CGP-447 | chr22 | 22053660 | 23264283 | -1.7644 | 8 |
| E10 | CGP-447 | chr22 | 23264283 | 51186435 | 0.2271 | 418 |
| E10 | CGP-447 | chrX | 2709879 | 53147034 | -0.1405 | 466 |
| E10 | CGP-447 | chrX | 53147034 | 53577363 | -3.1938 | 3 |
| E10 | CGP-447 | chrX | 53577363 | 57873050 | 0.071 | 32 |
| E10 | CGP-447 | chrX | 61931753 | 154882523 | -0.1692 | 766 |
| E10 | CGP-447 | chrY | 2654633 | 9535491 | -0.1951 | 43 |
| E10 | CGP-447 | chrY | 13199529 | 19586679 | -0.1116 | 37 |
| E10 | CGP-447 | chrY | 21033995 | 24521747 | -0.0813 | 18 |
| E10 | CGP-447 | chrY | 28463067 | 28590746 | -0.0151 | 5 |
| E4 | CGP-254 | chr1 | 93709 | 51400153 | 0.1793 | 633 |
| E4 | CGP-254 | chr1 | 51400153 | 51478162 | 2.4926 | 3 |
| E4 | CGP-254 | chr1 | 51478162 | 121286064 | -0.0287 | 657 |
| E4 | CGP-254 | chr1 | 142633228 | 249153501 | 0.0806 | 1032 |
| E4 | CGP-254 | chr2 | 85992 | 47556925 | 0.0208 | 479 |
| E4 | CGP-254 | chr2 | 47556925 | 48032461 | 0.427 | 49 |
| E4 | CGP-254 | chr2 | 48032461 | 58348445 | 0.0335 | 79 |
| E4 | CGP-254 | chr2 | 58348445 | 58493089 | -0.3209 | 18 |
| E4 | CGP-254 | chr2 | 58493089 | 61120252 | 0.1277 | 21 |
| E4 | CGP-254 | chr2 | 61120252 | 62694944 | 0.3931 | 46 |
| E4 | CGP-254 | chr2 | 62694944 | 90458314 | 0.0364 | 204 |
| E4 | CGP-254 | chr2 | 91668610 | 92252664 | 0.1818 | 5 |
| E4 | CGP-254 | chr2 | 95389406 | 141021341 | -0.0258 | 454 |
| E4 | CGP-254 | chr2 | 141021341 | 142928543 | -0.4377 | 114 |
| E4 | CGP-254 | chr2 | 142928543 | 243170925 | -0.0884 | 1018 |
| E4 | CGP-254 | chr3 | 133006 | 9461972 | -0.0472 | 69 |
| E4 | CGP-254 | chr3 | 9461972 | 10232101 | 0.4666 | 51 |
| E4 | CGP-254 | chr3 | 10232101 | 12628173 | 0.0166 | 22 |
| E4 | CGP-254 | chr3 | 12628173 | 12804714 | 0.3044 | 36 |
| E4 | CGP-254 | chr3 | 12804714 | 37036633 | -0.1075 | 189 |
| E4 | CGP-254 | chr3 | 37036633 | 37379793 | 0.2992 | 22 |
| E4 | CGP-254 | chr3 | 37379793 | 47074725 | -0.0541 | 95 |
| E4 | CGP-254 | chr3 | 47074725 | 47159731 | 0.4203 | 21 |
| E4 | CGP-254 | chr3 | 47159731 | 49242497 | 0.1683 | 35 |
| E4 | CGP-254 | chr3 | 49242497 | 49700204 | 0.6823 | 8 |
| E4 | CGP-254 | chr3 | 49700204 | 52596631 | -0.0471 | 45 |
| E4 | CGP-254 | chr3 | 52596631 | 54055431 | 0.3061 | 39 |
| E4 | CGP-254 | chr3 | 54055431 | 90264750 | -0.0996 | 330 |
| E4 | CGP-254 | chr3 | 93518057 | 197888969 | -0.1125 | 1123 |
| E4 | CGP-254 | chr4 | 79870 | 1904436 | 0.0519 | 61 |
| E4 | CGP-254 | chr4 | 1904436 | 1938583 | 0.6106 | 8 |
| E4 | CGP-254 | chr4 | 1938583 | 49574529 | -0.0829 | 363 |
| E4 | CGP-254 | chr4 | 52678380 | 66147806 | -0.0986 | 177 |
| E4 | CGP-254 | chr4 | 66147806 | 66478224 | -0.4719 | 22 |
| E4 | CGP-254 | chr4 | 66478224 | 190976528 | -0.0687 | 960 |
| E4 | CGP-254 | chr5 | 114213 | 46176124 | -0.1123 | 501 |
| E4 | CGP-254 | chr5 | 49488216 | 67484361 | -0.0375 | 158 |
| E4 | CGP-254 | chr5 | 67484361 | 67741868 | -0.4148 | 22 |
| E4 | CGP-254 | chr5 | 67741868 | 68925095 | -0.065 | 8 |
| E4 | CGP-254 | chr5 | 68925095 | 69664612 | 0.6953 | 5 |
| E4 | CGP-254 | chr5 | 69664612 | 125115904 | -0.116 | 515 |
| E4 | CGP-254 | chr5 | 125115904 | 125930363 | 0.3193 | 23 |
| E4 | CGP-254 | chr5 | 125930363 | 176552797 | -0.0503 | 570 |
| E4 | CGP-254 | chr5 | 176552797 | 176717463 | 0.3835 | 36 |
| E4 | CGP-254 | chr5 | 176717463 | 180697842 | 0.0229 | 66 |
| E4 | CGP-254 | chr6 | 143533 | 25989013 | -0.0011 | 217 |
| E4 | CGP-254 | chr6 | 25989013 | 26266687 | -0.7168 | 10 |
| E4 | CGP-254 | chr6 | 26266687 | 28617731 | 0.0659 | 17 |
| E4 | CGP-254 | chr6 | 33390431 | 35423714 | 0.2496 | 19 |
| E4 | CGP-254 | chr6 | 35423714 | 35470229 | 0.6837 | 10 |
| E4 | CGP-254 | chr6 | 35470229 | 58710554 | -0.0649 | 338 |
| E4 | CGP-254 | chr6 | 61948509 | 93637039 | -0.0643 | 248 |
| E4 | CGP-254 | chr6 | 93637039 | 94316593 | -0.6053 | 24 |
| E4 | CGP-254 | chr6 | 94316593 | 128134957 | -0.2059 | 381 |
| E4 | CGP-254 | chr6 | 128134957 | 128514353 | -0.8583 | 33 |
| E4 | CGP-254 | chr6 | 128514353 | 132909408 | -0.5473 | 46 |
| E4 | CGP-254 | chr6 | 132909408 | 158969603 | -1.0266 | 383 |
| E4 | CGP-254 | chr6 | 158969603 | 170982158 | -0.2562 | 141 |
| E4 | CGP-254 | chr7 | 121242 | 6015199 | 0.1126 | 67 |
| E4 | CGP-254 | chr7 | 6015199 | 6429289 | 0.5206 | 24 |
| E4 | CGP-254 | chr7 | 6429289 | 8261909 | 0.1539 | 18 |
| E4 | CGP-254 | chr7 | 8261909 | 55656427 | -0.1014 | 616 |
| E4 | CGP-254 | chr7 | 55656427 | 57974477 | 0.1947 | 15 |
| E4 | CGP-254 | chr7 | 61118627 | 80550543 | 0.1374 | 162 |
| E4 | CGP-254 | chr7 | 80550543 | 96165478 | -0.2076 | 206 |
| E4 | CGP-254 | chr7 | 96165478 | 98479215 | 0.0682 | 18 |
| E4 | CGP-254 | chr7 | 98479215 | 98505150 | 0.412 | 11 |
| E4 | CGP-254 | chr7 | 98505150 | 98546686 | 0.0884 | 23 |
| E4 | CGP-254 | chr7 | 98546686 | 98554876 | -0.2817 | 8 |
| E4 | CGP-254 | chr7 | 98554876 | 98566524 | 0.166 | 8 |
| E4 | CGP-254 | chr7 | 98566524 | 98643135 | -0.2362 | 22 |
| E4 | CGP-254 | chr7 | 98643135 | 104721499 | 0.2174 | 138 |
| E4 | CGP-254 | chr7 | 104721499 | 105173033 | 0.4681 | 4 |
| E4 | CGP-254 | chr7 | 105173033 | 106505760 | -0.0549 | 10 |
| E4 | CGP-254 | chr7 | 106505760 | 106509266 | -0.9369 | 5 |
| E4 | CGP-254 | chr7 | 106509266 | 159081217 | -0.0882 | 790 |
| E4 | CGP-254 | chr8 | 85563 | 43599475 | -0.0322 | 557 |
| E4 | CGP-254 | chr8 | 46894817 | 67481967 | -0.0174 | 242 |
| E4 | CGP-254 | chr8 | 67481967 | 67495570 | 0.552 | 7 |
| E4 | CGP-254 | chr8 | 67495570 | 92932743 | -0.114 | 208 |
| E4 | CGP-254 | chr8 | 92932743 | 93424459 | -0.5172 | 23 |
| E4 | CGP-254 | chr8 | 93424459 | 144042539 | -0.0943 | 406 |
| E4 | CGP-254 | chr8 | 144042539 | 146224687 | 0.2515 | 40 |
| E4 | CGP-254 | chr9 | 85972 | 2036308 | -0.0359 | 16 |
| E4 | CGP-254 | chr9 | 2036308 | 2057564 | -0.6556 | 5 |
| E4 | CGP-254 | chr9 | 2057564 | 21970403 | -0.1593 | 308 |
| E4 | CGP-254 | chr9 | 21970403 | 21979558 | 3.1208 | 2 |
| E4 | CGP-254 | chr9 | 21979558 | 22051139 | -0.8707 | 5 |
| E4 | CGP-254 | chr9 | 22051139 | 47238906 | -0.098 | 190 |
| E4 | CGP-254 | chr9 | 65543126 | 98060940 | -0.0853 | 348 |
| E4 | CGP-254 | chr9 | 98060940 | 98226873 | -0.5398 | 10 |
| E4 | CGP-254 | chr9 | 98226873 | 125272953 | -0.053 | 268 |
| E4 | CGP-254 | chr9 | 125272953 | 127916061 | 0.1791 | 22 |
| E4 | CGP-254 | chr9 | 127916061 | 127946957 | 0.7348 | 5 |
| E4 | CGP-254 | chr9 | 127946957 | 141079652 | 0.1626 | 189 |
| E4 | CGP-254 | chr10 | 132498 | 38976500 | 0.0269 | 283 |
| E4 | CGP-254 | chr10 | 42701112 | 46448181 | -0.1679 | 56 |
| E4 | CGP-254 | chr10 | 46448181 | 48303330 | 0.433 | 12 |
| E4 | CGP-254 | chr10 | 48303330 | 62852007 | -0.1006 | 102 |
| E4 | CGP-254 | chr10 | 62852007 | 64016404 | -0.3996 | 29 |
| E4 | CGP-254 | chr10 | 64016404 | 74100669 | 0.0157 | 74 |
| E4 | CGP-254 | chr10 | 74100669 | 74963080 | 0.7596 | 6 |
| E4 | CGP-254 | chr10 | 74963080 | 135442100 | 0.0133 | 656 |
| E4 | CGP-254 | chr11 | 139102 | 3699263 | 0.0779 | 33 |
| E4 | CGP-254 | chr11 | 3699263 | 3801866 | 0.5447 | 31 |
| E4 | CGP-254 | chr11 | 3801866 | 50401441 | -0.0194 | 384 |
| E4 | CGP-254 | chr11 | 51404033 | 51530481 | 0.1642 | 2 |
| E4 | CGP-254 | chr11 | 55028536 | 88551560 | 0.0649 | 339 |
| E4 | CGP-254 | chr11 | 88551560 | 89892500 | 0.3977 | 9 |
| E4 | CGP-254 | chr11 | 89892500 | 105916113 | -0.0403 | 157 |
| E4 | CGP-254 | chr11 | 105916113 | 107049317 | -0.401 | 27 |
| E4 | CGP-254 | chr11 | 107049317 | 118319366 | -0.0108 | 152 |
| E4 | CGP-254 | chr11 | 118319366 | 119169762 | 0.2169 | 85 |
| E4 | CGP-254 | chr11 | 119169762 | 134873894 | -0.0643 | 143 |
| E4 | CGP-254 | chr12 | 77870 | 443118 | 0.1295 | 24 |
| E4 | CGP-254 | chr12 | 443118 | 494178 | 0.4837 | 10 |
| E4 | CGP-254 | chr12 | 494178 | 6679984 | -0.0589 | 66 |
| E4 | CGP-254 | chr12 | 6679984 | 6709263 | 0.3769 | 30 |
| E4 | CGP-254 | chr12 | 6709263 | 34611982 | -0.0905 | 377 |
| E4 | CGP-254 | chr12 | 37936847 | 51199958 | -0.0954 | 326 |
| E4 | CGP-254 | chr12 | 51199958 | 51920911 | 0.4261 | 18 |
| E4 | CGP-254 | chr12 | 51920911 | 53663621 | 0.0375 | 27 |
| E4 | CGP-254 | chr12 | 53663621 | 53681211 | 0.3791 | 19 |
| E4 | CGP-254 | chr12 | 53681211 | 55541154 | 0.1043 | 28 |
| E4 | CGP-254 | chr12 | 55541154 | 56538100 | 0.3841 | 35 |
| E4 | CGP-254 | chr12 | 56538100 | 57494150 | 0.1259 | 19 |
| E4 | CGP-254 | chr12 | 57494150 | 57498970 | 0.3556 | 11 |
| E4 | CGP-254 | chr12 | 57498970 | 57549064 | -0.0169 | 22 |
| E4 | CGP-254 | chr12 | 57549064 | 57911328 | 0.2192 | 23 |
| E4 | CGP-254 | chr12 | 57911328 | 77856397 | -0.0152 | 172 |
| E4 | CGP-254 | chr12 | 77856397 | 78755764 | -0.4174 | 61 |
| E4 | CGP-254 | chr12 | 78755764 | 89704131 | -0.0467 | 78 |
| E4 | CGP-254 | chr12 | 89704131 | 89778908 | -0.7817 | 5 |
| E4 | CGP-254 | chr12 | 89778908 | 110259830 | -0.0609 | 150 |
| E4 | CGP-254 | chr12 | 110259830 | 111868724 | 0.3316 | 16 |
| E4 | CGP-254 | chr12 | 111868724 | 112470525 | -0.0742 | 12 |
| E4 | CGP-254 | chr12 | 112470525 | 113246214 | 0.4531 | 27 |
| E4 | CGP-254 | chr12 | 113246214 | 133756620 | 0.0648 | 222 |
| E4 | CGP-254 | chr13 | 19104211 | 28225066 | -0.0469 | 96 |
| E4 | CGP-254 | chr13 | 28225066 | 28996012 | 0.2519 | 57 |
| E4 | CGP-254 | chr13 | 28996012 | 33262028 | -0.0299 | 105 |
| E4 | CGP-254 | chr13 | 33262028 | 34807078 | -0.2168 | 36 |
| E4 | CGP-254 | chr13 | 34807078 | 35968557 | 0.2419 | 9 |
| E4 | CGP-254 | chr13 | 35968557 | 41190436 | -0.0622 | 225 |
| E4 | CGP-254 | chr13 | 41190436 | 41237893 | 0.1918 | 128 |
| E4 | CGP-254 | chr13 | 41237893 | 48954276 | -0.0547 | 77 |
| E4 | CGP-254 | chr13 | 48954276 | 51474173 | 0.1618 | 31 |
| E4 | CGP-254 | chr13 | 51474173 | 78434681 | -0.0724 | 194 |
| E4 | CGP-254 | chr13 | 78434681 | 78638573 | -0.6403 | 12 |
| E4 | CGP-254 | chr13 | 78638573 | 80754259 | -0.0161 | 15 |
| E4 | CGP-254 | chr13 | 80754259 | 81156759 | -0.6417 | 7 |
| E4 | CGP-254 | chr13 | 81156759 | 86332937 | -0.0828 | 36 |
| E4 | CGP-254 | chr13 | 86332937 | 86403256 | -0.5143 | 10 |
| E4 | CGP-254 | chr13 | 86403256 | 115017146 | -0.0773 | 227 |
| E4 | CGP-254 | chr14 | 19110470 | 107211129 | 0.036 | 881 |
| E4 | CGP-254 | chr15 | 20098915 | 21067713 | 0.0345 | 12 |
| E4 | CGP-254 | chr15 | 21067713 | 22209860 | 0.7823 | 5 |
| E4 | CGP-254 | chr15 | 22209860 | 40951251 | 0.0814 | 224 |
| E4 | CGP-254 | chr15 | 40951251 | 41062194 | 0.7271 | 9 |
| E4 | CGP-254 | chr15 | 41062194 | 42057751 | 0.4355 | 68 |
| E4 | CGP-254 | chr15 | 42057751 | 75498071 | 0.1629 | 278 |
| E4 | CGP-254 | chr15 | 75498071 | 75704598 | 0.5504 | 18 |
| E4 | CGP-254 | chr15 | 75704598 | 88642225 | 0.2318 | 322 |
| E4 | CGP-254 | chr15 | 88642225 | 88652012 | 0.4812 | 28 |
| E4 | CGP-254 | chr15 | 88652012 | 88652796 | 0.0555 | 3 |
| E4 | CGP-254 | chr15 | 88652796 | 88658739 | 0.4255 | 21 |
| E4 | CGP-254 | chr15 | 88658739 | 88699610 | 0.1969 | 70 |
| E4 | CGP-254 | chr15 | 88699610 | 89571550 | -0.1118 | 10 |
| E4 | CGP-254 | chr15 | 89571550 | 90807145 | 0.1652 | 20 |
| E4 | CGP-254 | chr15 | 90807145 | 91551749 | 0.4327 | 31 |
| E4 | CGP-254 | chr15 | 91551749 | 97811656 | 0.1532 | 45 |
| E4 | CGP-254 | chr15 | 97811656 | 102435161 | -0.0282 | 57 |
| E4 | CGP-254 | chr16 | 129775 | 357193 | 0.5316 | 10 |
| E4 | CGP-254 | chr16 | 357193 | 8354316 | 0.2946 | 177 |
| E4 | CGP-254 | chr16 | 8354316 | 8989249 | -0.2117 | 8 |
| E4 | CGP-254 | chr16 | 8989249 | 23576258 | 0.2121 | 164 |
| E4 | CGP-254 | chr16 | 23576258 | 23640818 | 0.813 | 8 |
| E4 | CGP-254 | chr16 | 23640818 | 23650745 | 0.5024 | 11 |
| E4 | CGP-254 | chr16 | 23650745 | 35208242 | 0.2011 | 119 |
| E4 | CGP-254 | chr16 | 46443771 | 67067132 | 0.0754 | 217 |
| E4 | CGP-254 | chr16 | 67067132 | 69059185 | 0.4989 | 89 |
| E4 | CGP-254 | chr16 | 69059185 | 71689884 | 0.1633 | 24 |
| E4 | CGP-254 | chr16 | 71689884 | 71730542 | 0.3844 | 13 |
| E4 | CGP-254 | chr16 | 71730542 | 89806156 | 0.0321 | 203 |
| E4 | CGP-254 | chr16 | 89806156 | 90225734 | 0.4743 | 44 |
| E4 | CGP-254 | chr17 | 74407 | 1249084 | 0.1846 | 10 |
| E4 | CGP-254 | chr17 | 1249084 | 1264868 | 0.6334 | 18 |
| E4 | CGP-254 | chr17 | 1264868 | 5051202 | 0.2396 | 67 |
| E4 | CGP-254 | chr17 | 5051202 | 5115172 | 0.5622 | 11 |
| E4 | CGP-254 | chr17 | 5115172 | 7572357 | 0.1579 | 20 |
| E4 | CGP-254 | chr17 | 7572357 | 7844435 | 0.607 | 14 |
| E4 | CGP-254 | chr17 | 7844435 | 22188081 | 0.1689 | 203 |
| E4 | CGP-254 | chr17 | 25293739 | 40357227 | 0.2188 | 310 |
| E4 | CGP-254 | chr17 | 40357227 | 40398439 | 0.5913 | 15 |
| E4 | CGP-254 | chr17 | 40398439 | 40476908 | 0.2517 | 26 |
| E4 | CGP-254 | chr17 | 40476908 | 41276630 | 0.5634 | 77 |
| E4 | CGP-254 | chr17 | 41276630 | 43411931 | 0.2206 | 17 |
| E4 | CGP-254 | chr17 | 43411931 | 44854668 | -0.4606 | 8 |
| E4 | CGP-254 | chr17 | 44854668 | 56059710 | 0.1727 | 150 |
| E4 | CGP-254 | chr17 | 56059710 | 56426701 | -0.2882 | 14 |
| E4 | CGP-254 | chr17 | 56426701 | 81100038 | 0.1841 | 479 |
| E4 | CGP-254 | chr18 | 91321 | 15321481 | -0.046 | 110 |
| E4 | CGP-254 | chr18 | 18528883 | 78009413 | -0.166 | 635 |
| E4 | CGP-254 | chr19 | 275354 | 7118443 | 0.3541 | 146 |
| E4 | CGP-254 | chr19 | 7118443 | 7179567 | 0.7419 | 19 |
| E4 | CGP-254 | chr19 | 7179567 | 14210156 | 0.3718 | 122 |
| E4 | CGP-254 | chr19 | 14210156 | 14222491 | 0.6834 | 11 |
| E4 | CGP-254 | chr19 | 14222491 | 14224063 | 0.3621 | 6 |
| E4 | CGP-254 | chr19 | 14224063 | 14383039 | 0.1164 | 17 |
| E4 | CGP-254 | chr19 | 14383039 | 15271990 | 0.4115 | 9 |
| E4 | CGP-254 | chr19 | 15271990 | 15339819 | 0.6204 | 36 |
| E4 | CGP-254 | chr19 | 15339819 | 16554315 | 0.0465 | 35 |
| E4 | CGP-254 | chr19 | 16554315 | 24397434 | 0.4217 | 102 |
| E4 | CGP-254 | chr19 | 28283158 | 30306010 | -0.0297 | 17 |
| E4 | CGP-254 | chr19 | 30306010 | 31205725 | 0.4403 | 14 |
| E4 | CGP-254 | chr19 | 31205725 | 32277346 | -0.1485 | 25 |
| E4 | CGP-254 | chr19 | 32277346 | 41745374 | 0.2619 | 164 |
| E4 | CGP-254 | chr19 | 41745374 | 41764499 | 0.837 | 10 |
| E4 | CGP-254 | chr19 | 41764499 | 45857296 | 0.2289 | 83 |
| E4 | CGP-254 | chr19 | 45857296 | 45913444 | 0.5842 | 17 |
| E4 | CGP-254 | chr19 | 45913444 | 50865692 | 0.2236 | 70 |
| E4 | CGP-254 | chr19 | 50865692 | 50912262 | 0.6304 | 14 |
| E4 | CGP-254 | chr19 | 50912262 | 59042306 | 0.2576 | 88 |
| E4 | CGP-254 | chr20 | 133210 | 26237715 | -0.0386 | 197 |
| E4 | CGP-254 | chr20 | 29478404 | 62886763 | 0.0294 | 496 |
| E4 | CGP-254 | chr21 | 9464761 | 11106090 | -0.0743 | 10 |
| E4 | CGP-254 | chr21 | 14424112 | 42856429 | -0.0156 | 440 |
| E4 | CGP-254 | chr21 | 42856429 | 42874816 | 0.2663 | 31 |
| E4 | CGP-254 | chr21 | 42874816 | 48074388 | -0.0281 | 67 |
| E4 | CGP-254 | chr22 | 16131356 | 28540153 | 0.2202 | 128 |
| E4 | CGP-254 | chr22 | 28540153 | 29266311 | 0.6727 | 20 |
| E4 | CGP-254 | chr22 | 29266311 | 29672174 | 0.0127 | 8 |
| E4 | CGP-254 | chr22 | 29672174 | 29684325 | 0.5946 | 22 |
| E4 | CGP-254 | chr22 | 29684325 | 29691427 | 0.3687 | 20 |
| E4 | CGP-254 | chr22 | 29691427 | 29692876 | 0.7401 | 5 |
| E4 | CGP-254 | chr22 | 29692876 | 29695476 | 0.4497 | 6 |
| E4 | CGP-254 | chr22 | 29695476 | 41507208 | 0.1204 | 205 |
| E4 | CGP-254 | chr22 | 41507208 | 41543336 | 0.6279 | 12 |
| E4 | CGP-254 | chr22 | 41543336 | 41547025 | 0.171 | 4 |
| E4 | CGP-254 | chr22 | 41547025 | 41571053 | 0.4983 | 15 |
| E4 | CGP-254 | chr22 | 41571053 | 42021084 | 0.1066 | 14 |
| E4 | CGP-254 | chr22 | 42021084 | 42522794 | 0.6308 | 18 |
| E4 | CGP-254 | chr22 | 42522794 | 51186431 | 0.0998 | 72 |
| E4 | CGP-254 | chrX | 2709879 | 57873044 | 0.0324 | 677 |
| E4 | CGP-254 | chrX | 61931754 | 70482445 | -0.025 | 166 |
| E4 | CGP-254 | chrX | 70482445 | 70613628 | 0.3551 | 24 |
| E4 | CGP-254 | chrX | 70613628 | 70638845 | 0.7062 | 8 |
| E4 | CGP-254 | chrX | 70638845 | 70782447 | 0.2991 | 15 |
| E4 | CGP-254 | chrX | 70782447 | 154882514 | -0.0609 | 800 |
| E4 | CGP-254 | chrY | 2654634 | 9546570 | -3.4791 | 41 |
| E4 | CGP-254 | chrY | 13199534 | 19506576 | -3.1753 | 30 |
| E4 | CGP-254 | chrY | 21034002 | 24521735 | -3.3513 | 15 |
| E4 | CGP-254 | chrY | 28463074 | 28590731 | -2.7378 | 5 |
| E2 | CGP-252 | chr1 | 93709 | 27048167 | 0.1462 | 356 |
| E2 | CGP-252 | chr1 | 27048167 | 27058607 | -0.741 | 3 |
| E2 | CGP-252 | chr1 | 27058607 | 51400153 | 0.0688 | 274 |
| E2 | CGP-252 | chr1 | 51400153 | 51478162 | 2.0496 | 3 |
| E2 | CGP-252 | chr1 | 51478162 | 121286064 | -0.0827 | 657 |
| E2 | CGP-252 | chr1 | 142633228 | 161295827 | 0.0616 | 235 |
| E2 | CGP-252 | chr1 | 161295827 | 161374524 | 0.6991 | 8 |
| E2 | CGP-252 | chr1 | 161374524 | 249153501 | -0.008 | 788 |
| E2 | CGP-252 | chr2 | 85992 | 25993015 | -0.074 | 237 |
| E2 | CGP-252 | chr2 | 25993015 | 26076547 | 0.5266 | 8 |
| E2 | CGP-252 | chr2 | 26076547 | 47556925 | -0.0248 | 234 |
| E2 | CGP-252 | chr2 | 47556925 | 48032461 | 0.3223 | 49 |
| E2 | CGP-252 | chr2 | 48032461 | 61140101 | -0.0755 | 121 |
| E2 | CGP-252 | chr2 | 61140101 | 61148368 | 0.3727 | 5 |
| E2 | CGP-252 | chr2 | 61148368 | 61657523 | 0.0231 | 7 |
| E2 | CGP-252 | chr2 | 61657523 | 62072676 | 0.4191 | 27 |
| E2 | CGP-252 | chr2 | 62072676 | 89150233 | -0.0362 | 201 |
| E2 | CGP-252 | chr2 | 89150233 | 90458314 | -0.866 | 7 |
| E2 | CGP-252 | chr2 | 91668610 | 92252664 | 0.3406 | 5 |
| E2 | CGP-252 | chr2 | 95389406 | 243170925 | -0.075 | 1587 |
| E2 | CGP-252 | chr3 | 133006 | 9613926 | -0.089 | 70 |
| E2 | CGP-252 | chr3 | 9613926 | 10232101 | 0.4482 | 50 |
| E2 | CGP-252 | chr3 | 10232101 | 11710419 | -0.1059 | 11 |
| E2 | CGP-252 | chr3 | 11710419 | 12317935 | 0.3651 | 5 |
| E2 | CGP-252 | chr3 | 12317935 | 12626964 | -0.1829 | 5 |
| E2 | CGP-252 | chr3 | 12626964 | 12948690 | 0.2218 | 38 |
| E2 | CGP-252 | chr3 | 12948690 | 37036633 | -0.1499 | 187 |
| E2 | CGP-252 | chr3 | 37036633 | 37236265 | 0.2932 | 21 |
| E2 | CGP-252 | chr3 | 37236265 | 45466792 | -0.0872 | 81 |
| E2 | CGP-252 | chr3 | 45466792 | 47159731 | 0.2574 | 36 |
| E2 | CGP-252 | chr3 | 47159731 | 49242497 | 0.0691 | 35 |
| E2 | CGP-252 | chr3 | 49242497 | 50440477 | 0.4722 | 13 |
| E2 | CGP-252 | chr3 | 50440477 | 52539963 | -0.1154 | 33 |
| E2 | CGP-252 | chr3 | 52539963 | 53872691 | 0.2505 | 44 |
| E2 | CGP-252 | chr3 | 53872691 | 90264750 | -0.0592 | 332 |
| E2 | CGP-252 | chr3 | 93518057 | 197888969 | -0.0768 | 1124 |
| E2 | CGP-252 | chr4 | 79870 | 49574529 | -0.0196 | 432 |
| E2 | CGP-252 | chr4 | 52678380 | 190976528 | -0.0557 | 1159 |
| E2 | CGP-252 | chr5 | 114213 | 1281507 | -0.0077 | 38 |
| E2 | CGP-252 | chr5 | 1281507 | 1348533 | 0.4974 | 7 |
| E2 | CGP-252 | chr5 | 1348533 | 46176124 | -0.0684 | 456 |
| E2 | CGP-252 | chr5 | 49488216 | 170817093 | -0.0611 | 1228 |
| E2 | CGP-252 | chr5 | 170817093 | 171041520 | 0.2983 | 15 |
| E2 | CGP-252 | chr5 | 171041520 | 176519580 | 0.0044 | 45 |
| E2 | CGP-252 | chr5 | 176519580 | 176719997 | 0.269 | 50 |
| E2 | CGP-252 | chr5 | 176719997 | 179970263 | -0.1291 | 30 |
| E2 | CGP-252 | chr5 | 179970263 | 180835787 | 0.2385 | 36 |
| E2 | CGP-252 | chr6 | 143533 | 25989013 | 0.0119 | 217 |
| E2 | CGP-252 | chr6 | 25989013 | 26266687 | -0.6822 | 10 |
| E2 | CGP-252 | chr6 | 26266687 | 28617731 | -0.0177 | 17 |
| E2 | CGP-252 | chr6 | 33390431 | 58710554 | -0.0145 | 369 |
| E2 | CGP-252 | chr6 | 61948509 | 138198988 | -0.0892 | 901 |
| E2 | CGP-252 | chr6 | 138198988 | 138238793 | -0.7182 | 6 |
| E2 | CGP-252 | chr6 | 138238793 | 157458158 | -0.0617 | 170 |
| E2 | CGP-252 | chr6 | 157458158 | 157695093 | -0.4795 | 28 |
| E2 | CGP-252 | chr6 | 157695093 | 170982158 | -0.1711 | 150 |
| E2 | CGP-252 | chr7 | 121242 | 5862783 | 0.1106 | 65 |
| E2 | CGP-252 | chr7 | 5862783 | 6047097 | 0.6125 | 17 |
| E2 | CGP-252 | chr7 | 6047097 | 6904582 | 0.1071 | 17 |
| E2 | CGP-252 | chr7 | 6904582 | 27889915 | -0.1097 | 229 |
| E2 | CGP-252 | chr7 | 27889915 | 27901261 | 0.4662 | 12 |
| E2 | CGP-252 | chr7 | 27901261 | 27926116 | -0.0353 | 82 |
| E2 | CGP-252 | chr7 | 27926116 | 27933769 | 0.2465 | 23 |
| E2 | CGP-252 | chr7 | 27933769 | 33000260 | -0.1123 | 52 |
| E2 | CGP-252 | chr7 | 33000260 | 33488889 | 0.3544 | 16 |
| E2 | CGP-252 | chr7 | 33488889 | 55480393 | -0.1066 | 210 |
| E2 | CGP-252 | chr7 | 55480393 | 57974477 | 0.3521 | 17 |
| E2 | CGP-252 | chr7 | 61118627 | 74158483 | 0.257 | 105 |
| E2 | CGP-252 | chr7 | 74158483 | 76715401 | 0.6887 | 29 |
| E2 | CGP-252 | chr7 | 76715401 | 97491328 | -0.1037 | 243 |
| E2 | CGP-252 | chr7 | 97491328 | 98546686 | 0.2112 | 43 |
| E2 | CGP-252 | chr7 | 98546686 | 99369035 | -0.0623 | 70 |
| E2 | CGP-252 | chr7 | 99369035 | 102175189 | 0.2709 | 89 |
| E2 | CGP-252 | chr7 | 102175189 | 105616303 | -0.0452 | 24 |
| E2 | CGP-252 | chr7 | 105616303 | 106509524 | -0.5964 | 13 |
| E2 | CGP-252 | chr7 | 106509524 | 151979935 | -0.0781 | 717 |
| E2 | CGP-252 | chr7 | 151979935 | 152361645 | 0.3194 | 17 |
| E2 | CGP-252 | chr7 | 152361645 | 159081217 | -0.0688 | 55 |
| E2 | CGP-252 | chr8 | 85563 | 11405616 | -0.0896 | 114 |
| E2 | CGP-252 | chr8 | 11405616 | 12294150 | 0.6095 | 7 |
| E2 | CGP-252 | chr8 | 12294150 | 37964270 | -0.0243 | 303 |
| E2 | CGP-252 | chr8 | 37964270 | 38611897 | 0.2391 | 46 |
| E2 | CGP-252 | chr8 | 38611897 | 43599475 | -0.0801 | 87 |
| E2 | CGP-252 | chr8 | 46894817 | 146224687 | -0.0496 | 926 |
| E2 | CGP-252 | chr9 | 85972 | 47238906 | -0.0817 | 525 |
| E2 | CGP-252 | chr9 | 65543126 | 141079652 | -0.0319 | 843 |
| E2 | CGP-252 | chr10 | 132498 | 38976500 | 0.0059 | 283 |
| E2 | CGP-252 | chr10 | 42701112 | 135442100 | -0.0199 | 934 |
| E2 | CGP-252 | chr11 | 139102 | 3699263 | 0.125 | 33 |
| E2 | CGP-252 | chr11 | 3699263 | 3839040 | 0.4878 | 32 |
| E2 | CGP-252 | chr11 | 3839040 | 50401441 | -0.0256 | 383 |
| E2 | CGP-252 | chr11 | 51404033 | 51530481 | 0.4688 | 2 |
| E2 | CGP-252 | chr11 | 55028536 | 99453981 | -0.0067 | 435 |
| E2 | CGP-252 | chr11 | 99453981 | 116965989 | -0.7145 | 235 |
| E2 | CGP-252 | chr11 | 116965989 | 134873894 | 0.0281 | 240 |
| E2 | CGP-252 | chr12 | 77870 | 1964042 | 0.1214 | 49 |
| E2 | CGP-252 | chr12 | 1964042 | 6515260 | -0.2337 | 49 |
| E2 | CGP-252 | chr12 | 6515260 | 6691944 | 0.4617 | 13 |
| E2 | CGP-252 | chr12 | 6691944 | 6694554 | -0.1408 | 3 |
| E2 | CGP-252 | chr12 | 6694554 | 6709263 | 0.4161 | 16 |
| E2 | CGP-252 | chr12 | 6709263 | 34611982 | -0.0628 | 379 |
| E2 | CGP-252 | chr12 | 37936847 | 51045350 | -0.0531 | 321 |
| E2 | CGP-252 | chr12 | 51045350 | 51210811 | 0.5336 | 17 |
| E2 | CGP-252 | chr12 | 51210811 | 53663981 | -0.0219 | 34 |
| E2 | CGP-252 | chr12 | 53663981 | 53681211 | 0.3975 | 18 |
| E2 | CGP-252 | chr12 | 53681211 | 56149335 | 0.0479 | 32 |
| E2 | CGP-252 | chr12 | 56149335 | 57164668 | 0.3652 | 36 |
| E2 | CGP-252 | chr12 | 57164668 | 57491736 | -0.062 | 6 |
| E2 | CGP-252 | chr12 | 57491736 | 57498704 | 0.2795 | 18 |
| E2 | CGP-252 | chr12 | 57498704 | 57813298 | -0.0205 | 25 |
| E2 | CGP-252 | chr12 | 57813298 | 57911328 | 0.2387 | 21 |
| E2 | CGP-252 | chr12 | 57911328 | 112877322 | -0.0471 | 499 |
| E2 | CGP-252 | chr12 | 112877322 | 112980708 | 0.5967 | 20 |
| E2 | CGP-252 | chr12 | 112980708 | 133756620 | 0.0417 | 224 |
| E2 | CGP-252 | chr13 | 19104211 | 28566523 | -0.0043 | 100 |
| E2 | CGP-252 | chr13 | 28566523 | 28616931 | 0.4207 | 17 |
| E2 | CGP-252 | chr13 | 28616931 | 41215499 | -0.026 | 478 |
| E2 | CGP-252 | chr13 | 41215499 | 41216865 | 0.5374 | 4 |
| E2 | CGP-252 | chr13 | 41216865 | 41237893 | 0.1647 | 57 |
| E2 | CGP-252 | chr13 | 41237893 | 115017146 | -0.0972 | 609 |
| E2 | CGP-252 | chr14 | 19110470 | 106352684 | -0.0088 | 876 |
| E2 | CGP-252 | chr14 | 106352684 | 107211129 | -0.6863 | 6 |
| E2 | CGP-252 | chr15 | 20098915 | 40832600 | 0.0337 | 241 |
| E2 | CGP-252 | chr15 | 40832600 | 42057751 | 0.3869 | 78 |
| E2 | CGP-252 | chr15 | 42057751 | 88642489 | 0.105 | 619 |
| E2 | CGP-252 | chr15 | 88642489 | 88645796 | 0.492 | 7 |
| E2 | CGP-252 | chr15 | 88645796 | 88652012 | 0.3263 | 20 |
| E2 | CGP-252 | chr15 | 88652012 | 88652796 | -0.0701 | 3 |
| E2 | CGP-252 | chr15 | 88652796 | 88658200 | 0.3065 | 19 |
| E2 | CGP-252 | chr15 | 88658200 | 91250133 | 0.053 | 105 |
| E2 | CGP-252 | chr15 | 91250133 | 91551749 | 0.3461 | 28 |
| E2 | CGP-252 | chr15 | 91551749 | 102435161 | -0.0203 | 102 |
| E2 | CGP-252 | chr16 | 129775 | 23576258 | 0.1581 | 359 |
| E2 | CGP-252 | chr16 | 23576258 | 23640818 | 0.6931 | 8 |
| E2 | CGP-252 | chr16 | 23640818 | 31195430 | 0.2015 | 79 |
| E2 | CGP-252 | chr16 | 31195430 | 31486144 | -0.1331 | 25 |
| E2 | CGP-252 | chr16 | 31486144 | 35208242 | 0.5033 | 26 |
| E2 | CGP-252 | chr16 | 46443771 | 67093137 | 0.0085 | 219 |
| E2 | CGP-252 | chr16 | 67093137 | 68905705 | 0.366 | 85 |
| E2 | CGP-252 | chr16 | 68905705 | 71689884 | 0.0662 | 26 |
| E2 | CGP-252 | chr16 | 71689884 | 71879760 | 0.3561 | 19 |
| E2 | CGP-252 | chr16 | 71879760 | 89661529 | -0.0264 | 192 |
| E2 | CGP-252 | chr16 | 89661529 | 90225734 | 0.3961 | 49 |
| E2 | CGP-252 | chr17 | 74407 | 22188081 | 0.1626 | 343 |
| E2 | CGP-252 | chr17 | 25293739 | 40357227 | 0.1494 | 310 |
| E2 | CGP-252 | chr17 | 40357227 | 44964425 | 0.3694 | 144 |
| E2 | CGP-252 | chr17 | 44964425 | 56059710 | 0.0618 | 149 |
| E2 | CGP-252 | chr17 | 56059710 | 56426701 | -0.3516 | 14 |
| E2 | CGP-252 | chr17 | 56426701 | 78539523 | 0.1209 | 417 |
| E2 | CGP-252 | chr17 | 78539523 | 78910565 | -0.2846 | 37 |
| E2 | CGP-252 | chr17 | 78910565 | 81100038 | 0.1048 | 25 |
| E2 | CGP-252 | chr18 | 91321 | 15321481 | 0.0224 | 110 |
| E2 | CGP-252 | chr18 | 18528883 | 78009413 | -0.1054 | 635 |
| E2 | CGP-252 | chr19 | 275354 | 7118443 | 0.2446 | 146 |
| E2 | CGP-252 | chr19 | 7118443 | 7179567 | 0.6073 | 19 |
| E2 | CGP-252 | chr19 | 7179567 | 14204249 | 0.2421 | 118 |
| E2 | CGP-252 | chr19 | 14204249 | 14223262 | 0.5401 | 18 |
| E2 | CGP-252 | chr19 | 14223262 | 14224063 | 0.3092 | 3 |
| E2 | CGP-252 | chr19 | 14224063 | 14537202 | 0.0408 | 18 |
| E2 | CGP-252 | chr19 | 14537202 | 15272855 | 0.2797 | 10 |
| E2 | CGP-252 | chr19 | 15272855 | 15339819 | 0.5125 | 34 |
| E2 | CGP-252 | chr19 | 15339819 | 17897829 | -0.0041 | 44 |
| E2 | CGP-252 | chr19 | 17897829 | 19792695 | 0.4354 | 60 |
| E2 | CGP-252 | chr19 | 19792695 | 24397434 | 0.1544 | 33 |
| E2 | CGP-252 | chr19 | 28283158 | 30306010 | 0.1091 | 17 |
| E2 | CGP-252 | chr19 | 30306010 | 30352027 | 0.5399 | 8 |
| E2 | CGP-252 | chr19 | 30352027 | 31734347 | 0.0775 | 11 |
| E2 | CGP-252 | chr19 | 31734347 | 31790109 | -0.4258 | 13 |
| E2 | CGP-252 | chr19 | 31790109 | 41747226 | 0.1759 | 172 |
| E2 | CGP-252 | chr19 | 41747226 | 41764499 | 0.7426 | 9 |
| E2 | CGP-252 | chr19 | 41764499 | 45857296 | 0.1741 | 83 |
| E2 | CGP-252 | chr19 | 45857296 | 45873610 | 0.5152 | 16 |
| E2 | CGP-252 | chr19 | 45873610 | 59042306 | 0.1696 | 173 |
| E2 | CGP-252 | chr20 | 133210 | 26237715 | -0.0407 | 197 |
| E2 | CGP-252 | chr20 | 29478404 | 52196518 | -0.008 | 347 |
| E2 | CGP-252 | chr20 | 52196518 | 52350554 | -0.6625 | 6 |
| E2 | CGP-252 | chr20 | 52350554 | 57439694 | 0.0463 | 59 |
| E2 | CGP-252 | chr20 | 57439694 | 57522501 | -0.3653 | 15 |
| E2 | CGP-252 | chr20 | 57522501 | 62886763 | 0.0383 | 69 |
| E2 | CGP-252 | chr21 | 9464761 | 11106090 | 0.435 | 10 |
| E2 | CGP-252 | chr21 | 14424112 | 48074388 | -0.0012 | 538 |
| E2 | CGP-252 | chr22 | 16131356 | 29047485 | 0.14 | 132 |
| E2 | CGP-252 | chr22 | 29047485 | 29266311 | 0.5783 | 16 |
| E2 | CGP-252 | chr22 | 29266311 | 29672174 | -0.1153 | 8 |
| E2 | CGP-252 | chr22 | 29672174 | 30405074 | 0.3576 | 79 |
| E2 | CGP-252 | chr22 | 30405074 | 41495093 | 0.0116 | 178 |
| E2 | CGP-252 | chr22 | 41495093 | 41534961 | 0.5793 | 10 |
| E2 | CGP-252 | chr22 | 41534961 | 41571053 | 0.2637 | 22 |
| E2 | CGP-252 | chr22 | 41571053 | 42021084 | 0.0416 | 14 |
| E2 | CGP-252 | chr22 | 42021084 | 42058547 | 0.7336 | 13 |
| E2 | CGP-252 | chr22 | 42058547 | 51186431 | 0.0467 | 77 |
| E2 | CGP-252 | chrX | 2709879 | 57873044 | 0.0221 | 678 |
| E2 | CGP-252 | chrX | 61931754 | 70482445 | 0.006 | 165 |
| E2 | CGP-252 | chrX | 70482445 | 70699955 | 0.3961 | 45 |
| E2 | CGP-252 | chrX | 70699955 | 154882514 | -0.0386 | 802 |
| E2 | CGP-252 | chrY | 2654634 | 9546570 | -0.2237 | 44 |
| E2 | CGP-252 | chrY | 13199534 | 19586678 | -0.1337 | 37 |
| E2 | CGP-252 | chrY | 21034002 | 24521735 | -0.0794 | 17 |
| E2 | CGP-252 | chrY | 27495899 | 28590731 | -0.1747 | 6 |
| E1 | CGP-251 | chr1 | 93709 | 10480536 | 0.0912 | 104 |
| E1 | CGP-251 | chr1 | 10480536 | 11187949 | 0.3052 | 20 |
| E1 | CGP-251 | chr1 | 11187949 | 11193838 | -0.2029 | 6 |
| E1 | CGP-251 | chr1 | 11193838 | 11608866 | 0.2857 | 40 |
| E1 | CGP-251 | chr1 | 11608866 | 45144977 | -0.0055 | 378 |
| E1 | CGP-251 | chr1 | 45144977 | 46225432 | 0.4394 | 46 |
| E1 | CGP-251 | chr1 | 46225432 | 46807145 | -0.5577 | 5 |
| E1 | CGP-251 | chr1 | 46807145 | 51400153 | -0.032 | 33 |
| E1 | CGP-251 | chr1 | 51400153 | 51478162 | 1.2554 | 3 |
| E1 | CGP-251 | chr1 | 51478162 | 59210248 | -0.105 | 55 |
| E1 | CGP-251 | chr1 | 59210248 | 59287493 | -0.8238 | 4 |
| E1 | CGP-251 | chr1 | 59287493 | 120566456 | -0.1172 | 591 |
| E1 | CGP-251 | chr1 | 120566456 | 121286064 | -0.6546 | 7 |
| E1 | CGP-251 | chr1 | 142633228 | 148962481 | -0.1722 | 46 |
| E1 | CGP-251 | chr1 | 148962481 | 149485192 | -2.185 | 3 |
| E1 | CGP-251 | chr1 | 149485192 | 249153501 | -0.0357 | 983 |
| E1 | CGP-251 | chr2 | 85992 | 47439433 | 0.3088 | 478 |
| E1 | CGP-251 | chr2 | 47439433 | 47646858 | 0.8923 | 19 |
| E1 | CGP-251 | chr2 | 47646858 | 47970824 | 0.5364 | 15 |
| E1 | CGP-251 | chr2 | 47970824 | 48024486 | 0.9111 | 4 |
| E1 | CGP-251 | chr2 | 48024486 | 58233612 | 0.3736 | 90 |
| E1 | CGP-251 | chr2 | 58233612 | 58493089 | -0.0378 | 19 |
| E1 | CGP-251 | chr2 | 58493089 | 61120252 | 0.3627 | 21 |
| E1 | CGP-251 | chr2 | 61120252 | 61734497 | 0.7004 | 36 |
| E1 | CGP-251 | chr2 | 61734497 | 70278403 | 0.3331 | 66 |
| E1 | CGP-251 | chr2 | 70278403 | 70365637 | -0.3786 | 4 |
| E1 | CGP-251 | chr2 | 70365637 | 90458314 | 0.2769 | 144 |
| E1 | CGP-251 | chr2 | 91668610 | 92252664 | 0.3611 | 5 |
| E1 | CGP-251 | chr2 | 95389406 | 131541238 | 0.2653 | 376 |
| E1 | CGP-251 | chr2 | 131541238 | 133358779 | -0.268 | 13 |
| E1 | CGP-251 | chr2 | 133358779 | 135300625 | -1.0583 | 14 |
| E1 | CGP-251 | chr2 | 135300625 | 155703078 | -0.1726 | 269 |
| E1 | CGP-251 | chr2 | 155703078 | 159629986 | -0.8774 | 40 |
| E1 | CGP-251 | chr2 | 159629986 | 165303964 | 0.1412 | 40 |
| E1 | CGP-251 | chr2 | 165303964 | 170853045 | -0.8743 | 38 |
| E1 | CGP-251 | chr2 | 170853045 | 228904495 | -0.1175 | 629 |
| E1 | CGP-251 | chr2 | 228904495 | 230674312 | -0.9459 | 13 |
| E1 | CGP-251 | chr2 | 230674312 | 234548465 | -0.082 | 65 |
| E1 | CGP-251 | chr2 | 234548465 | 234845525 | -0.9291 | 32 |
| E1 | CGP-251 | chr2 | 234845525 | 236103661 | 0.2569 | 9 |
| E1 | CGP-251 | chr2 | 236103661 | 236705453 | -0.7104 | 4 |
| E1 | CGP-251 | chr2 | 236705453 | 238876508 | 0.1517 | 15 |
| E1 | CGP-251 | chr2 | 238876508 | 239958208 | -0.9838 | 7 |
| E1 | CGP-251 | chr2 | 239958208 | 240795136 | -0.1052 | 6 |
| E1 | CGP-251 | chr2 | 240795136 | 243170925 | 0.1139 | 17 |
| E1 | CGP-251 | chr3 | 133006 | 9917835 | -0.1513 | 72 |
| E1 | CGP-251 | chr3 | 9917835 | 10153087 | 0.3071 | 44 |
| E1 | CGP-251 | chr3 | 10153087 | 37036633 | -0.0922 | 251 |
| E1 | CGP-251 | chr3 | 37036633 | 37236265 | 0.2015 | 21 |
| E1 | CGP-251 | chr3 | 37236265 | 47065757 | -0.141 | 95 |
| E1 | CGP-251 | chr3 | 47065757 | 47159731 | 0.3483 | 22 |
| E1 | CGP-251 | chr3 | 47159731 | 49242497 | -0.0716 | 35 |
| E1 | CGP-251 | chr3 | 49242497 | 49588879 | 0.6943 | 7 |
| E1 | CGP-251 | chr3 | 49588879 | 52596631 | -0.1789 | 46 |
| E1 | CGP-251 | chr3 | 52596631 | 52713103 | 0.2396 | 28 |
| E1 | CGP-251 | chr3 | 52713103 | 90264750 | -0.1058 | 341 |
| E1 | CGP-251 | chr3 | 93518057 | 197888969 | -0.1321 | 1124 |
| E1 | CGP-251 | chr4 | 79870 | 49574529 | -0.0719 | 432 |
| E1 | CGP-251 | chr4 | 52678380 | 190976528 | -0.0929 | 1158 |
| E1 | CGP-251 | chr5 | 114213 | 46176124 | -0.1213 | 501 |
| E1 | CGP-251 | chr5 | 49488216 | 180697842 | -0.0961 | 1402 |
| E1 | CGP-251 | chr6 | 143533 | 25860754 | -0.065 | 216 |
| E1 | CGP-251 | chr6 | 25860754 | 26266687 | -0.8824 | 11 |
| E1 | CGP-251 | chr6 | 26266687 | 28617731 | -0.0713 | 17 |
| E1 | CGP-251 | chr6 | 33390431 | 35423714 | 0.0358 | 19 |
| E1 | CGP-251 | chr6 | 35423714 | 35470229 | 0.5568 | 10 |
| E1 | CGP-251 | chr6 | 35470229 | 58710554 | -0.0603 | 340 |
| E1 | CGP-251 | chr6 | 61948509 | 170982158 | -0.1474 | 1255 |
| E1 | CGP-251 | chr7 | 121242 | 6028316 | 0.0633 | 73 |
| E1 | CGP-251 | chr7 | 6028316 | 6429289 | 0.5156 | 18 |
| E1 | CGP-251 | chr7 | 6429289 | 7058588 | 0.1392 | 9 |
| E1 | CGP-251 | chr7 | 7058588 | 27889915 | -0.1737 | 228 |
| E1 | CGP-251 | chr7 | 27889915 | 27901261 | 0.3564 | 12 |
| E1 | CGP-251 | chr7 | 27901261 | 27924153 | -0.1741 | 76 |
| E1 | CGP-251 | chr7 | 27924153 | 27933769 | 0.1276 | 29 |
| E1 | CGP-251 | chr7 | 27933769 | 33011535 | -0.1544 | 54 |
| E1 | CGP-251 | chr7 | 33011535 | 33088097 | 0.2492 | 11 |
| E1 | CGP-251 | chr7 | 33088097 | 41575540 | -0.0633 | 61 |
| E1 | CGP-251 | chr7 | 41575540 | 41974980 | -0.4732 | 9 |
| E1 | CGP-251 | chr7 | 41974980 | 50436608 | -0.127 | 67 |
| E1 | CGP-251 | chr7 | 50436608 | 50504290 | -0.6306 | 8 |
| E1 | CGP-251 | chr7 | 50504290 | 57974477 | -0.1209 | 85 |
| E1 | CGP-251 | chr7 | 61118627 | 62112788 | -0.2446 | 3 |
| E1 | CGP-251 | chr7 | 62112788 | 74127299 | 0.2122 | 93 |
| E1 | CGP-251 | chr7 | 74127299 | 99369035 | -0.1061 | 394 |
| E1 | CGP-251 | chr7 | 99369035 | 102323482 | 0.2526 | 90 |
| E1 | CGP-251 | chr7 | 102323482 | 106505760 | -0.1014 | 30 |
| E1 | CGP-251 | chr7 | 106505760 | 106509524 | -0.9136 | 6 |
| E1 | CGP-251 | chr7 | 106509524 | 159081217 | -0.1237 | 789 |
| E1 | CGP-251 | chr8 | 85563 | 43599475 | -0.0707 | 557 |
| E1 | CGP-251 | chr8 | 46894817 | 48645034 | -0.2636 | 12 |
| E1 | CGP-251 | chr8 | 48645034 | 48737902 | 0.1474 | 23 |
| E1 | CGP-251 | chr8 | 48737902 | 67481967 | -0.0976 | 207 |
| E1 | CGP-251 | chr8 | 67481967 | 67501677 | 0.5 | 8 |
| E1 | CGP-251 | chr8 | 67501677 | 144042539 | -0.1373 | 636 |
| E1 | CGP-251 | chr8 | 144042539 | 145370814 | 0.1568 | 33 |
| E1 | CGP-251 | chr8 | 145370814 | 146224687 | -0.2716 | 7 |
| E1 | CGP-251 | chr9 | 85972 | 47238906 | -0.1568 | 526 |
| E1 | CGP-251 | chr9 | 65543126 | 141079652 | -0.0852 | 842 |
| E1 | CGP-251 | chr10 | 132498 | 38976500 | -0.0312 | 283 |
| E1 | CGP-251 | chr10 | 42701112 | 63620034 | -0.1398 | 174 |
| E1 | CGP-251 | chr10 | 63620034 | 64657784 | -0.3526 | 29 |
| E1 | CGP-251 | chr10 | 64657784 | 135442100 | -0.06 | 730 |
| E1 | CGP-251 | chr11 | 139102 | 3699263 | -0.0172 | 33 |
| E1 | CGP-251 | chr11 | 3699263 | 3945694 | 0.4095 | 33 |
| E1 | CGP-251 | chr11 | 3945694 | 50401441 | -0.0712 | 382 |
| E1 | CGP-251 | chr11 | 51404033 | 51530481 | -0.0981 | 2 |
| E1 | CGP-251 | chr11 | 55028536 | 83102894 | -0.0036 | 300 |
| E1 | CGP-251 | chr11 | 83102894 | 116558868 | -0.8588 | 368 |
| E1 | CGP-251 | chr11 | 116558868 | 134873894 | -0.0473 | 244 |
| E1 | CGP-251 | chr12 | 77870 | 447596 | -0.0087 | 25 |
| E1 | CGP-251 | chr12 | 447596 | 494178 | 0.3409 | 9 |
| E1 | CGP-251 | chr12 | 494178 | 3770918 | -0.0302 | 28 |
| E1 | CGP-251 | chr12 | 3770918 | 4701956 | -0.2822 | 23 |
| E1 | CGP-251 | chr12 | 4701956 | 6515260 | -0.0165 | 13 |
| E1 | CGP-251 | chr12 | 6515260 | 6709263 | 0.2963 | 32 |
| E1 | CGP-251 | chr12 | 6709263 | 12026243 | -0.1375 | 135 |
| E1 | CGP-251 | chr12 | 12026243 | 12027298 | 0.8655 | 3 |
| E1 | CGP-251 | chr12 | 12027298 | 12029963 | -0.0138 | 8 |
| E1 | CGP-251 | chr12 | 12029963 | 12031941 | -0.3669 | 8 |
| E1 | CGP-251 | chr12 | 12031941 | 12833064 | -0.0337 | 56 |
| E1 | CGP-251 | chr12 | 12833064 | 12913796 | -0.7227 | 4 |
| E1 | CGP-251 | chr12 | 12913796 | 34611982 | -0.0707 | 165 |
| E1 | CGP-251 | chr12 | 37936847 | 46083370 | -0.1363 | 111 |
| E1 | CGP-251 | chr12 | 46083370 | 46124453 | -1.1311 | 2 |
| E1 | CGP-251 | chr12 | 46124453 | 49561652 | -0.1357 | 197 |
| E1 | CGP-251 | chr12 | 49561652 | 51779542 | 0.2157 | 33 |
| E1 | CGP-251 | chr12 | 51779542 | 53663621 | -0.0545 | 28 |
| E1 | CGP-251 | chr12 | 53663621 | 53681211 | 0.2986 | 19 |
| E1 | CGP-251 | chr12 | 53681211 | 56149335 | 0.0247 | 32 |
| E1 | CGP-251 | chr12 | 56149335 | 57164668 | 0.276 | 36 |
| E1 | CGP-251 | chr12 | 57164668 | 57491736 | -0.1758 | 6 |
| E1 | CGP-251 | chr12 | 57491736 | 57495235 | 0.1382 | 10 |
| E1 | CGP-251 | chr12 | 57495235 | 57497195 | 0.5598 | 4 |
| E1 | CGP-251 | chr12 | 57497195 | 57498970 | 0.1112 | 5 |
| E1 | CGP-251 | chr12 | 57498970 | 57813298 | -0.0902 | 24 |
| E1 | CGP-251 | chr12 | 57813298 | 57899450 | 0.2064 | 18 |
| E1 | CGP-251 | chr12 | 57899450 | 78306976 | -0.0493 | 180 |
| E1 | CGP-251 | chr12 | 78306976 | 78642262 | -0.3658 | 55 |
| E1 | CGP-251 | chr12 | 78642262 | 89704131 | -0.0899 | 79 |
| E1 | CGP-251 | chr12 | 89704131 | 89778908 | -0.7422 | 5 |
| E1 | CGP-251 | chr12 | 89778908 | 112470525 | -0.0785 | 178 |
| E1 | CGP-251 | chr12 | 112470525 | 112980708 | 0.4502 | 25 |
| E1 | CGP-251 | chr12 | 112980708 | 133756620 | -0.0652 | 224 |
| E1 | CGP-251 | chr13 | 19104211 | 28566523 | -0.0905 | 100 |
| E1 | CGP-251 | chr13 | 28566523 | 28616931 | 0.3517 | 17 |
| E1 | CGP-251 | chr13 | 28616931 | 29526179 | 0.0031 | 52 |
| E1 | CGP-251 | chr13 | 29526179 | 33003066 | -0.7918 | 76 |
| E1 | CGP-251 | chr13 | 33003066 | 34807078 | 0.1832 | 49 |
| E1 | CGP-251 | chr13 | 34807078 | 35290785 | -0.6481 | 4 |
| E1 | CGP-251 | chr13 | 35290785 | 41192361 | 0.2205 | 235 |
| E1 | CGP-251 | chr13 | 41192361 | 41214115 | 0.4095 | 58 |
| E1 | CGP-251 | chr13 | 41214115 | 41216865 | 0.7136 | 8 |
| E1 | CGP-251 | chr13 | 41216865 | 41237893 | 0.4836 | 57 |
| E1 | CGP-251 | chr13 | 41237893 | 41546095 | 0.1293 | 10 |
| E1 | CGP-251 | chr13 | 41546095 | 52808968 | -0.8769 | 108 |
| E1 | CGP-251 | chr13 | 52808968 | 115017146 | -0.152 | 491 |
| E1 | CGP-251 | chr14 | 19110470 | 20439194 | -0.5838 | 18 |
| E1 | CGP-251 | chr14 | 20439194 | 107211129 | -0.0568 | 864 |
| E1 | CGP-251 | chr15 | 20098915 | 20947654 | -0.0407 | 11 |
| E1 | CGP-251 | chr15 | 20947654 | 21250037 | 0.9008 | 3 |
| E1 | CGP-251 | chr15 | 21250037 | 40951251 | -0.0299 | 228 |
| E1 | CGP-251 | chr15 | 40951251 | 42057751 | 0.3113 | 77 |
| E1 | CGP-251 | chr15 | 42057751 | 99146646 | 0.0446 | 856 |
| E1 | CGP-251 | chr15 | 99146646 | 102435161 | -0.2417 | 47 |
| E1 | CGP-251 | chr16 | 129775 | 23576258 | 0.0958 | 359 |
| E1 | CGP-251 | chr16 | 23576258 | 23640818 | 0.6405 | 8 |
| E1 | CGP-251 | chr16 | 23640818 | 35208242 | 0.0846 | 130 |
| E1 | CGP-251 | chr16 | 46443771 | 66593883 | -0.0612 | 212 |
| E1 | CGP-251 | chr16 | 66593883 | 69173426 | 0.3145 | 95 |
| E1 | CGP-251 | chr16 | 69173426 | 72779951 | 0.0848 | 49 |
| E1 | CGP-251 | chr16 | 72779951 | 73036669 | -0.2917 | 48 |
| E1 | CGP-251 | chr16 | 73036669 | 89805758 | -0.0346 | 141 |
| E1 | CGP-251 | chr16 | 89805758 | 90225734 | 0.3243 | 45 |
| E1 | CGP-251 | chr17 | 74407 | 1249084 | -0.0527 | 10 |
| E1 | CGP-251 | chr17 | 1249084 | 1264868 | 0.4076 | 18 |
| E1 | CGP-251 | chr17 | 1264868 | 7572357 | 0.052 | 98 |
| E1 | CGP-251 | chr17 | 7572357 | 7844435 | 0.4871 | 14 |
| E1 | CGP-251 | chr17 | 7844435 | 11941262 | 0.0534 | 39 |
| E1 | CGP-251 | chr17 | 11941262 | 12025648 | -0.6305 | 13 |
| E1 | CGP-251 | chr17 | 12025648 | 21815958 | 0.0876 | 148 |
| E1 | CGP-251 | chr17 | 21815958 | 22188081 | -0.3479 | 3 |
| E1 | CGP-251 | chr17 | 25293739 | 30288582 | 0.0244 | 113 |
| E1 | CGP-251 | chr17 | 30288582 | 30532467 | 0.3518 | 32 |
| E1 | CGP-251 | chr17 | 30532467 | 37623241 | 0.0296 | 72 |
| E1 | CGP-251 | chr17 | 37623241 | 37678659 | 0.5745 | 14 |
| E1 | CGP-251 | chr17 | 37678659 | 37813748 | -0.1476 | 7 |
| E1 | CGP-251 | chr17 | 37813748 | 37912654 | 0.2336 | 30 |
| E1 | CGP-251 | chr17 | 37912654 | 40354115 | -0.0642 | 39 |
| E1 | CGP-251 | chr17 | 40354115 | 44124844 | 0.3192 | 142 |
| E1 | CGP-251 | chr17 | 44124844 | 81100038 | 0.0232 | 646 |
| E1 | CGP-251 | chr18 | 91321 | 15321481 | -0.068 | 110 |
| E1 | CGP-251 | chr18 | 18528883 | 78009413 | -0.1417 | 635 |
| E1 | CGP-251 | chr19 | 275354 | 15339819 | 0.2345 | 366 |
| E1 | CGP-251 | chr19 | 15339819 | 16831076 | -0.1087 | 37 |
| E1 | CGP-251 | chr19 | 16831076 | 17779031 | 0.2062 | 6 |
| E1 | CGP-251 | chr19 | 17779031 | 18134628 | 0.4767 | 27 |
| E1 | CGP-251 | chr19 | 18134628 | 24397434 | 0.195 | 67 |
| E1 | CGP-251 | chr19 | 28283158 | 30306010 | -0.0977 | 17 |
| E1 | CGP-251 | chr19 | 30306010 | 30352027 | 0.4787 | 8 |
| E1 | CGP-251 | chr19 | 30352027 | 31536321 | -0.0083 | 9 |
| E1 | CGP-251 | chr19 | 31536321 | 32422910 | -0.3234 | 23 |
| E1 | CGP-251 | chr19 | 32422910 | 41745374 | 0.1014 | 163 |
| E1 | CGP-251 | chr19 | 41745374 | 41764499 | 0.6403 | 10 |
| E1 | CGP-251 | chr19 | 41764499 | 42745350 | 0.1503 | 26 |
| E1 | CGP-251 | chr19 | 42745350 | 42796703 | -0.1114 | 20 |
| E1 | CGP-251 | chr19 | 42796703 | 45857296 | 0.1339 | 37 |
| E1 | CGP-251 | chr19 | 45857296 | 46346786 | 0.3971 | 20 |
| E1 | CGP-251 | chr19 | 46346786 | 50171252 | 0.0433 | 59 |
| E1 | CGP-251 | chr19 | 50171252 | 50920399 | 0.3461 | 32 |
| E1 | CGP-251 | chr19 | 50920399 | 59042306 | 0.0815 | 78 |
| E1 | CGP-251 | chr20 | 133210 | 26237715 | -0.1196 | 197 |
| E1 | CGP-251 | chr20 | 29478404 | 62886763 | -0.0904 | 496 |
| E1 | CGP-251 | chr21 | 9464761 | 11106090 | 0.1639 | 10 |
| E1 | CGP-251 | chr21 | 14424112 | 42849839 | -0.1018 | 438 |
| E1 | CGP-251 | chr21 | 42849839 | 43003135 | 0.075 | 54 |
| E1 | CGP-251 | chr21 | 43003135 | 48074388 | -0.1618 | 46 |
| E1 | CGP-251 | chr22 | 16131356 | 29047485 | 0.0819 | 132 |
| E1 | CGP-251 | chr22 | 29047485 | 29398790 | 0.5344 | 17 |
| E1 | CGP-251 | chr22 | 29398790 | 29672174 | -0.1956 | 7 |
| E1 | CGP-251 | chr22 | 29672174 | 29675389 | 0.1908 | 4 |
| E1 | CGP-251 | chr22 | 29675389 | 29682031 | 0.5559 | 12 |
| E1 | CGP-251 | chr22 | 29682031 | 29684325 | 0.3046 | 6 |
| E1 | CGP-251 | chr22 | 29684325 | 29687638 | 0.0955 | 12 |
| E1 | CGP-251 | chr22 | 29687638 | 29695476 | 0.3979 | 19 |
| E1 | CGP-251 | chr22 | 29695476 | 30024628 | -0.2147 | 6 |
| E1 | CGP-251 | chr22 | 30024628 | 30068624 | 0.341 | 11 |
| E1 | CGP-251 | chr22 | 30068624 | 41495093 | -0.0157 | 187 |
| E1 | CGP-251 | chr22 | 41495093 | 41534961 | 0.5035 | 10 |
| E1 | CGP-251 | chr22 | 41534961 | 41571053 | 0.2649 | 22 |
| E1 | CGP-251 | chr22 | 41571053 | 41574430 | -0.0932 | 7 |
| E1 | CGP-251 | chr22 | 41574430 | 42021084 | 0.203 | 7 |
| E1 | CGP-251 | chr22 | 42021084 | 42214221 | 0.5184 | 15 |
| E1 | CGP-251 | chr22 | 42214221 | 51186431 | -0.0082 | 75 |
| E1 | CGP-251 | chrX | 2709879 | 48890001 | -0.0693 | 532 |
| E1 | CGP-251 | chrX | 48890001 | 48894673 | 0.5407 | 7 |
| E1 | CGP-251 | chrX | 48894673 | 53181167 | 0.0808 | 47 |
| E1 | CGP-251 | chrX | 53181167 | 53235390 | -0.2171 | 18 |
| E1 | CGP-251 | chrX | 53235390 | 57873044 | 0.0572 | 74 |
| E1 | CGP-251 | chrX | 61931754 | 70482445 | -0.0633 | 166 |
| E1 | CGP-251 | chrX | 70482445 | 70617845 | 0.2818 | 26 |
| E1 | CGP-251 | chrX | 70617845 | 70638845 | 0.6503 | 6 |
| E1 | CGP-251 | chrX | 70638845 | 154882514 | -0.092 | 816 |
| E1 | CGP-251 | chrY | 2654634 | 9546570 | -0.1967 | 45 |
| E1 | CGP-251 | chrY | 13199534 | 19586678 | -0.2045 | 37 |
| E1 | CGP-251 | chrY | 21034002 | 24521735 | -0.1607 | 17 |
| E1 | CGP-251 | chrY | 27495899 | 28590731 | -0.067 | 6 |
